# Ancient Himalayan wolf (*Canis lupus chanco*) lineage in Upper Mustang of the Annapurna Conservation Area, Nepal

**DOI:** 10.3897/zookeys.582.5966

**Published:** 2016-04-21

**Authors:** Madhu Chetri, Yadvendradev V. Jhala, Shant R. Jnawali, Naresh Subedi, Maheshwar Dhakal, Bibek Yumnam

**Affiliations:** 1 Hedmark University of Applied Sciences, Norway; 2 Wildlife Institute of India, Chandrabani, Dehradun 248001, India; 3 WWF Nepal Hariyo Ban Program, Kathmandu, Nepal; 4 National Trust for Nature Conservation, Khumaltar, Lalitpur, Kathmandu, Nepal; 5 Department of National Parks and Wildlife Conservation, Babarmahal, Kathmandu, Nepal

**Keywords:** Himalayan wolf, wolf-dog clade, Canis lupus chanco, Trans-Himalaya, Annapurna Conservation Area, Nepal

## Abstract

The taxonomic status of the wolf (*Canis lupus*) in Nepal’s Trans-Himalaya is poorly understood. Recent genetic studies have revealed the existence of three lineages of wolves in the Indian sub-continent. Of these, the Himalayan wolf, *Canis lupus chanco*, has been reported to be the most ancient lineage historically distributed within the Nepal Himalaya. These wolves residing in the Trans-Himalayan region have been suggested to be smaller and very different from the European wolf. During October 2011, six fecal samples suspected to have originated from wolves were collected from Upper Mustang in the Annapurna Conservation Area of Nepal. DNA extraction and amplification of the mitochondrial (mt) control region (CR) locus yielded sequences from five out of six samples. One sample matched domestic dog sequences in GenBank, while the remaining four samples were aligned within the monophyletic and ancient Himalayan wolf clade. These four sequences which matched each other, were new and represented a novel Himalayan wolf haplotype. This result confirms that the endangered ancient Himalayan wolf is extant in Nepal. Detailed genomic study covering Nepal’s entire Himalayan landscape is recommended in order to understand their distribution, taxonomy and, genetic relatedness with other wolves potentially sharing the same landscape.

## Introduction

The presence of wolf (*Canis lupus*) in the Trans-Himalayan regions of Nepal has been reported for centuries (Hodgson 1847). The species is protected under the National Parks and Wildlife Conservation Act, 1973 of the Government of Nepal and listed as Critically Endangered in the National Red List (Jnawali et al. 2011). Although receiving federal protection status, wolves in this region have suffered heavy mortality, mainly due to retributive and preventive killing by livestock herders. Their imperiled status becomes all the more imminent for conservation in light of their evolutionary distinct origins from all other wolf lineages (Sharma et al. 2004, Aggarwal et al. 2007). Recent mitochondrial (mt) DNA analyses of wolves and dogs, from the Indian subcontinent, revealed the presence of three distinct wolf lineages in the region, which are basal and divergent to the globally distributed wolf-dog clade (Sharma et al. 2004). Further, the study also revealed that the Himalayan lineage of wolves (*Canis lupus chanco*) branches at an earlier point in the tree, and may have split as early as 0.8 to 1.5 million years ago (Sharma et al. 2004). Although recent studies reveal that the ancient Himalayan wolf lineage has been present in the Nepal Himalaya (Sharma et al. 2004), its current existence in Nepal is not certain. This is because documentation of the extant lineage was based on living wolf samples collected from Himachal Pradesh in India and DNA samples from Nepal were sourced entirely from museum specimens (Sharma et al. 2004).

**Figure 1.**
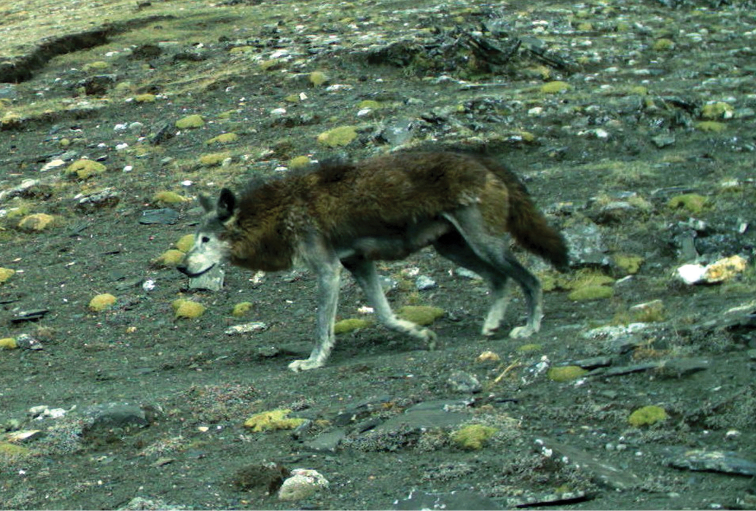
A Himalayan wolf photographed in Upper Mustang of Annapurna Conservation Area, Nepal (29.17356°N, 84.13422°E; datum WGS84, elevation 5,050 m) during May 2014.

During a recent survey in the Trans-Himalayan region of Upper Mustang, Annapurna Conservation Area, Nepal, wolves were encountered several times and their physical features were observed carefully (Figure 1). Characteristic features observed included distinct white coloration around the throat, chest, belly and inner part of the legs; woolliness of body fur; stumpy legs; unusual elongation of the muzzle, a muzzle arrayed with closely-spaced black speckles which extend below the eye on to the upper cheeks and ears; and smaller size compared to the European wolf (Gray 1863, Pocock 1941, Olsen and Olsen 1977). Based on these distinctive characteristics and skull morphology, Hodgson (1847) had classified this wolf as a separate species and called it *Canis laniger*. Subsequently, Pocock (1941) grouped *Canis laniger* with the Tibetan wolf subspecies (*Canis lupus chanco*). This taxonomic confusion regarding the identification and recognition of wolves from the Trans-Himalayan region of India and parts of Tibet has persisted for the last 165 years (Shrotriya et al. 2012). Aggarwal et al. (2007) claimed that the wolf ranging in the Trans-Himalayan landscape is a separate species or a subspecies of *Canis lupus*, although the recognition of separate species or subspecies is pending more evidence from nuclear markers (Habib et al. 2013). Based on mtDNA sequence data, *Canis lupus chanco* was observed to be paraphyletic and consist of two divergent and parapatric lineages extant in the region (Sharma et al. 2004). The Tibetan Plateau lineage of *Canis lupus chanco* occurs in western and central Kashmir, Tibet, China, Mongolia and Russia, and falls under the widespread wolf-dog clade. On the other hand, the basal monophyletic Himalayan wolf mtDNA lineage of *Canis lupus chanco* is distinct from haplotypes in the wolf-dog clade, and is likely distributed from eastern Kashmir into eastern Nepal and Tibet.

In the present paper, we describe for the first time the extant mitochondrial lineage of wolves that inhabit the Trans-Himalayan region in Upper Mustang of Annapurna Conservation Area, Nepal, based on DNA extracted from fecal samples collected in the wild. We identified a novel mtDNA CR haplotype that clustered within the monophyletic Himalayan wolf clade of *Canis lupus chanco*.

## Materials and methods

### Field Sampling and Labwork

**Figure 2.**
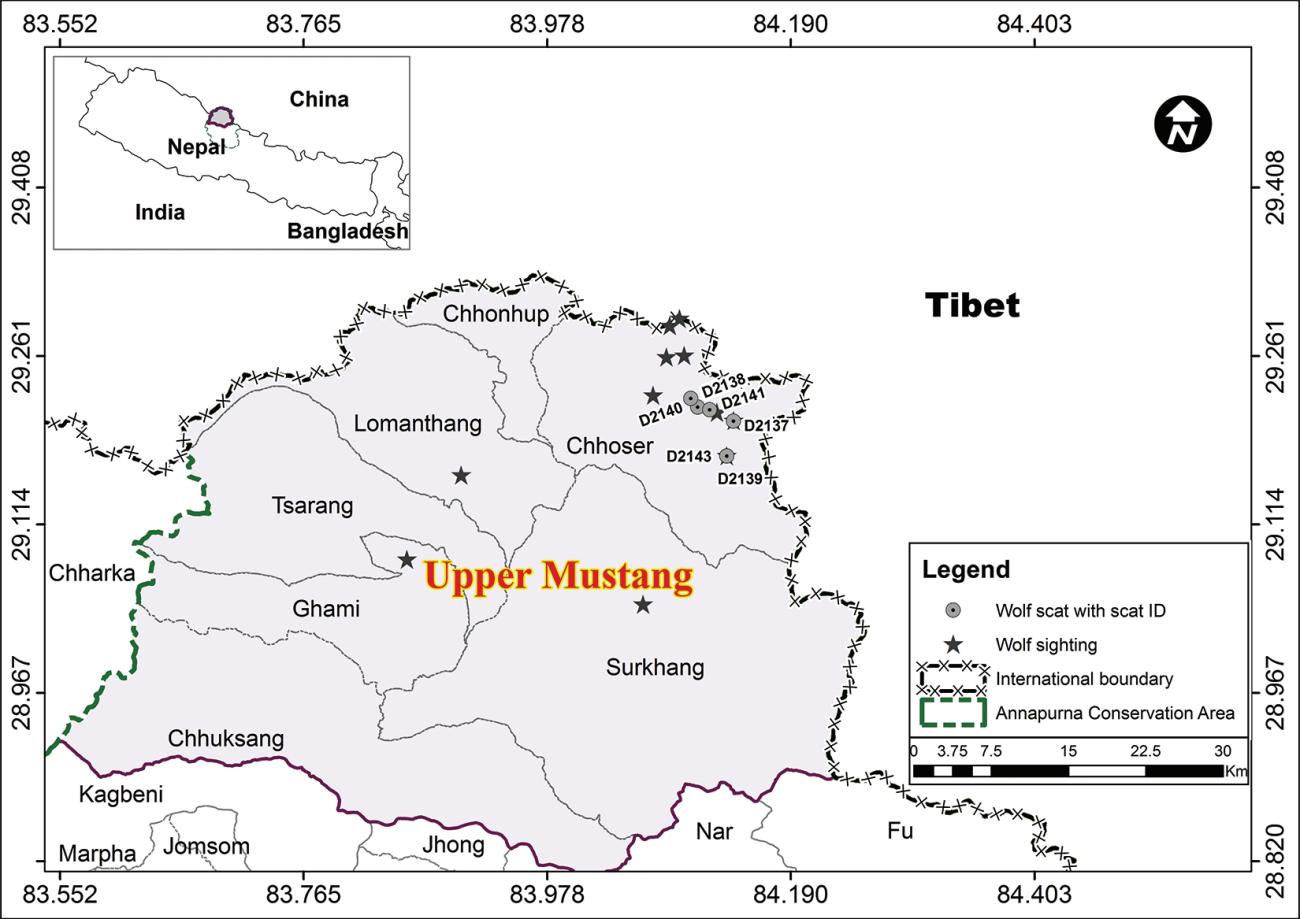
Fecal sample and direct sighting locations of the Himalayan wolf in Upper Mustang of Annapurna Conservation Area, Nepal.

During October 2011, six fecal samples suspected to have originated from wolves were collected from Upper Mustang in the Annapurna Conservation Area of Nepal at an elevation ranging from 4,750 to 5,050 m asl (Figure 2). A small portion of the collected fecal samples were preserved in polypropylene vials using silica desiccant (Janêcka et al. 2008, Lovari et al. 2009). Fecal DNA was extracted using standard protocol (QIAamp DNA stool kit, Qiagen Ag., Germany) and subsequent polymerase chain reaction (PCR) and DNA sequencing protocols followed methods outlined in Sharma et al. (2004). We PCR amplified the mtDNA control region (CR) locus using two sets of primer pairs, viz. - (i) ThrL15926 and DL-H16340 (Vila et al. 1999) targeted ~ 440 base pairs (bp); (ii) IWD 220 F and IWD 220 R targeted a smaller region (~ 200bp), especially designed for amplification of “ancient” canid samples (Sharma et al. 2004).

### Sequence analysis

MtDNA CR sequences were aligned and edited using Bioedit 5.0 (Hall 1999). Phylogenetic analyses were carried out using Bayesian and maximum likelihood procedures in MrBayes v3.2.2 (Ronquist et al. 2012) and MEGA 6 (Tamura et al. 2013) respectively. The Bayesian run length consisted of a total of 6 million Markov Chain Monte Carlo (MCMC) replicates, of which the first 1.5 million runs comprised the burn-in phase. Run convergence was assessed from the average standard deviation in split frequencies (< 0.01). Gaps were treated as missing data and not used for analysis in both MrBayes and MEGA. Trees were analyzed using the HKY (Hasegawa, Kishino and Yano, 1985), general time reversible (GTR, Tavare 1986), F81 (Felsenstein 1981) and mixed models of molecular evolution implemented in Mr Bayes. We analyzed the harmonic mean outputs of MrBayes runs using Bayes Factors (Kass and Raftery 1995) to obtain estimates of probabilities for the best model of molecular substitution for our data. The GTR substitution model with a gamma distributed rate variation and having a proportion of invariable sites (GTR + invgamma) was found to be the most likely model with the highest probability (P = 0.873) compared to all other models tested in this study (Suppl. material 1). The GTR + invgamma model was also implemented for maximum likelihood phylogeny construction in MEGA. Gaps were removed from analysis using the conservative ‘Complete Deletion’ option in MEGA. Confidence in estimated relationships was assessed using 1,000 bootstrap simulations. A maximum parsimony analysis was also conducted in MEGA for comparison. We compared our samples with corresponding sequences of other gray wolf lineages from the Indian-subcontinent and other regions, available at GenBank (see Suppl. material 2). The tree was rooted using sequence data from the maned wolf (*Chrysocyon brachyurus*) as outgroup, based on previously published phylogeny of canids (Lindblad-Toh et al. 2005). To estimate divergences and rates, we used MEGA to calculate mean Tamura-Nei genetic distances (with gamma shape parameter = 0.3) as used in previous studies on the same sequenced region in Himalayan wolves (Sharma et al. 2004). To examine genetic structuring among haplogroups, a median joining network tree (Bandelt et al. 1999) of CR haplotypes was constructed using the program network 4.613 (http://www.fluxus-engineering.com, Accessed 20 June 2015). Network calculations were carried out by assigning equal weights to all variable sites and with default values for the epsilon parameter (epsilon=0) in order to minimize alternative median networks. Gaps were treated as missing data and nucleotide alignment blocks containing indels were removed before analysis.

## Results and discussion

**Figure 3.**
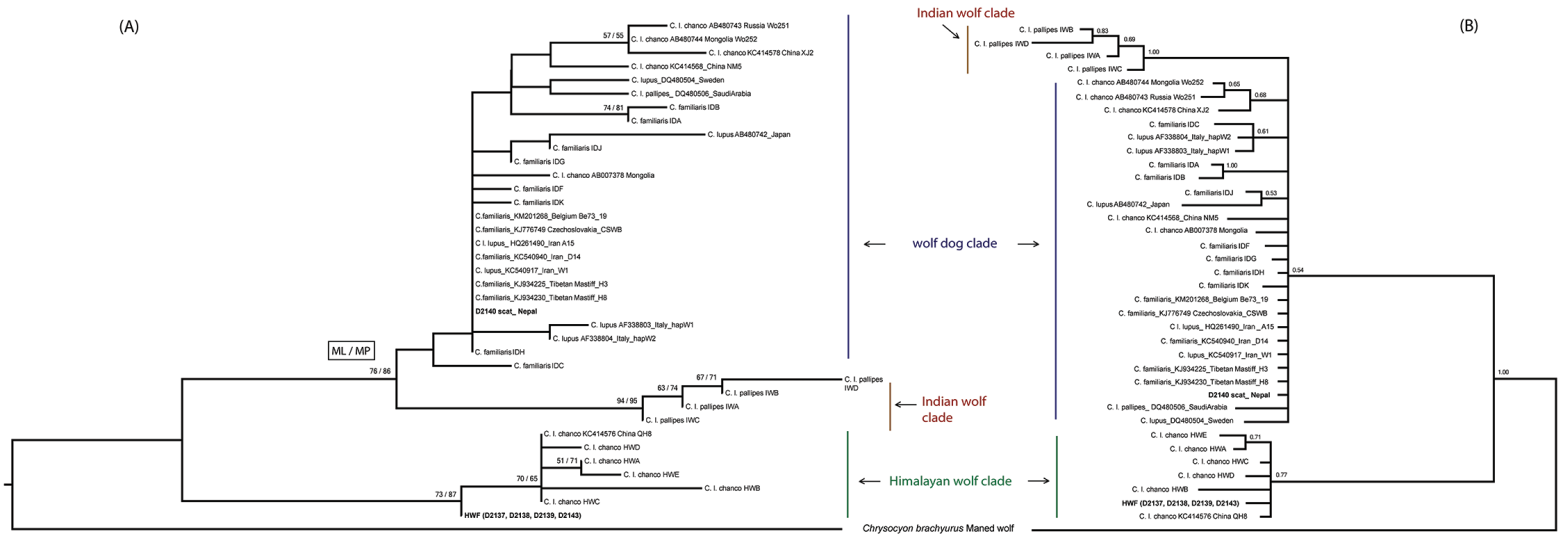
Phylogenetic trees constructed using 229 bp of aligned CR sequence data. The values at nodes correspond to **A** bootstrap support > 50% in maximum likelihood (ML) and maximum parsimony (MP) analyses; and **B** Bayesian posterior probability > 0.50. Scat samples sequenced in this study are highlighted in bold. Four samples (D2137, D2138, D2139 and D2143) represented a novel haplotype HWF within the Himalayan wolf clade, while a fifth sample (D2140) matched with existing domestic dog haplotypes.

We successfully obtained mtDNA control region sequences (~ 220 bp) from five (Table 1) out of a total of six fecal samples by using the shorter “ancient” DNA primer sets (see Suppl. material 3 for list of sequences). None of the six samples could be amplified using the larger (~ 440bp) primer pair. Such a result with scat samples is not unexpected, due to the already fragmented scat DNA extracts which render it difficult to amplify gene fragments larger than c. 200 to 300 bp (Janečka et al. 2008, Vynne et al. 2012). The species identity of one scat sample could not be established due to PCR failure. Out of the five successfully amplified fecal samples, the mtDNA sequences of four scats matched each other and were aligned within the monophyletic clade represented by the Himalayan wolf (Figure 3). The relationship was strongly supported with > 70% out of 1,000 bootstrap replicates in both maximum likelihood and maximum parsimony procedures (Figure 3A), and with > 0.75 posterior probability in Bayesian analysis (Figure 3B). Identical tree topologies were obtained in both the maximum likelihood and maximum parsimony analyses. Although there were minor differences in the resolution of few haplotypes in the maximum likelihood and Bayesian trees, the overall relationship was consistent with the Himalayan wolf haplotypes forming a basal clade to all other wolf lineages. The tree topology suggests that these four matched samples are derived individuals of the ancient Himalayan lineage of *Canis lupus chanco* and not the Tibetan wolf lineage of *Canis lupus chanco* which falls within the widespread wolf-dog clade. Additionally, the median-joining haplotype network analysis indicated that these scat sequences formed part of the Himalayan wolf lineage (Suppl. material 4), further lending support to the results of phylogenetic analyses.

**Table 1.** Specimen ID, location and area name with GenBank account number of the sequenced samples.

Specimen ID	Species	Clade	Locality (datum WGS84); Altitude (m)	Area name	Haplotype	GenBank Acc #	Date	Collector
D2137	*Canis lupus chanco*	Himalayan wolf	N29°12'14.3", E084°8'24.8"; 5020	Yarsa	HW-F	KT321360	11.10.2011	Madhu Chetri
D2138	*Canis lupus chanco*	Himalayan wolf	N29°12'58.4", E084°6'32.1"; 4740	Yarsa	HW-F	KT321360	11.10.2011	Madhu Chetri
D2139	*Canis lupus chanco*	Himalayan wolf	N29°10'24.2", E084°8'3.5"; 5050	Dharkeko pass	HW-F	KT321360	12.10.2011	Madhu Chetri
D2143	*Canis lupus chanco*	Himalayan wolf	N29°10'24.2", E084°8'3.5"; 5050	Dharkeko pass	HW-F	KT321360	12.10.2011	Madhu Chetri
D2140	*Canis familiaris*	Domestic dog	N29°13'25.5", E084°6'10.6"; 4740	Dhalung	ID-H	KT321361	14.10.2011	Madhu Chetri

The sequences of these four scat samples which clustered within the monophyletic Himalayan wolf clade are new and not identical to haplotypes identified previously in GenBank. We therefore designated this novel haplotype HWF, in line with the five existing HW (A to E) haplotypes identified previously by Sharma et al. (2004). Mean Tamura-Nei genetic distance between HWF and the rest of the Himalayan wolf haplotypes, corrected for intra-clade variation, was very low (1.2 ± 0.9 %), compared to significant divergence between members of the wolf-dog lineage of *Canis lupus chanco* (10.5 ± 5.5 %), and other wolf (9.2 ± 4.8 %) and dog lineages (10.1 ± 5.5 %). HWF differed from the nearest haplotype, HWC by two base substitutions and by three base substitutions from HWA (Suppl. material 3 and 4). Although not detected in our sampled sequences, both HWC and HWA haplotypes were previously reported in museum samples from Nepal (Sharma et al. 2004).

The sequence of a fifth scat sample fell within the domestic dog clade (*Canis familiaris*). BLAST analyses in GenBank (http://blast.ncbi.nlm.nih.gov/Blast.cgi) indicated 100% sequence match to many well represented domestic dog breeds from different regions of the world (see the Blast report pdf file in Suppl. material 5). Notably, these included the Indian domestic dog haplotype, ID-H, previously detected in Himalayan Bhotia sheepdogs (Sharma et al. 2004), Tibetan mastiffs from China, domestic dogs from Europe and the Middle East, and also a wolf individual from Iran carrying domestic dog introgressed mtDNA (Aghbolaghi et al. 2014). Given the proximity of sampled sites and niche overlap between wolves and dogs in the area, hybridization between the two species cannot altogether be ruled out. However, our sampling and analytical methods were inadequate for this purpose, and high resolution genome wide investigations using bi-parentally inherited markers are required for such hybridization studies.

The results of molecular analysis support our initial assumption, based on morphological observations, that the wolves found in Upper Mustang region of the Annapurna Conservation Area, Nepal, include individuals that belong to the genetically distinct and ancient Himalayan wolf clade (Figures 1 and 3). Although, it is plausible that both the Himalayan and Tibetan wolf (wolf-dog clade) lineages of *Canis lupus chanco* share the same landscapes (Sharma et al. 2004), we did not detect any individuals belonging to the latter clade. The remoteness of the terrain compounded by low population density of wolves in the area, made it difficult to locate and collect scats. However, despite our limited sampling we were able to detect four scats that originated from individuals aligned to the Himalayan wolf-dog clade of *Canis lupus chanco*. Given the close proximity of the sampled locations and absence of microsatellite genotypic information in our data, we are unable to confirm whether the sequences of these four scats originated from the same or from different individuals. Future noninvasive fecal sampling studies should cover the entire Himalayan landscape of Nepal so as to understand the distribution of gray wolf lineages in the region. Such surveys will also provide information on their population status and conservation threats.

As part of the ongoing long term ecological research on wolves, both formal and informal interviews with herders, livestock owners, nomads and village elite were conducted in order to understand the status of human-wolf conflict, local attitudes and perceptions. Formal interview involves semi-structured questionnaire survey (n=354) which covers all the potential areas of wolf distribution in Mustang and Manang Districts of Annapurna Conservation Area. Informal interview (n=61) was mainly through discussion when herders were encountered while herding their livestock or while visiting their herding camps/corrals. Our preliminary assessment revealed that local communities persecuted wolves mainly in retaliation for livestock depredation. In some parts of the conservation area, livestock depredation from wolves was found to be a cause of concern for local livelihoods. These genetically distinct Himalayan wolves deserve special conservation attention, at the same time that the conservation of this species in a context of human-wildlife conflict is challenging. A species action plan needs be formulated that develops mechanisms to minimize conflict, and strategies for motivating local communities towards wolf conservation.

## Supplementary materials

Supplementary material 1 Best model selection for Bayesian analysis Data type: DOCX file Explanation note: Estimating the best model of molecular substitution inferred using Log Bayes Factors (LBF) from Bayesian posterior distributions in Mr Bayes. Rate variation for tested models - gamma distributed rate variation across sites (gamma); gamma distributed with proportion of invariable sites (invgamma); rate variation with proportion of invariable sites (propinv); equal rate variation across sites (equal). This dataset is made available under the Open Database License (http://opendatacommons.org/licenses/odbl/1.0/). The Open Database License (ODbL) is a license agreement intended to allow users to freely share, modify, and use this Dataset while maintaining this same freedom for others, provided that the original source and author(s) are credited.Madhu Chetri, Yadvendradev V. Jhala, Shant R. Jnawali, Naresh Subedi, Maheshwar Dhakal, Bibek Yumnam

Supplementary material 2 Genebank sequences analysed in this study Data type: DOCX file Explanation note: GenBank accession numbers for sequences analyzed in this study. This dataset is made available under the Open Database License (http://opendatacommons.org/licenses/odbl/1.0/). The Open Database License (ODbL) is a license agreement intended to allow users to freely share, modify, and use this Dataset while maintaining this same freedom for others, provided that the original source and author(s) are credited.Madhu Chetri, Yadvendradev V. Jhala, Shant R. Jnawali, Naresh Subedi, Maheshwar Dhakal, Bibek Yumnam

Supplementary material 3 Aligned CR sequences of selected samples of wolves and dogs from GenBank, with the obtained scat samples Data type: DOCX file Explanation note: Aligned CR sequences of selected samples of wolves, dogs from GenBank, with scat samples obtained in this study. Numbers refer to mtDNA nucleotide positions referenced with respect to the complete mtDNA genome of gray wolf (GenBank Accession No. KF857179). Scat sequences D2137, D2138, D2139 and D2143 match each other differs from known Himalayan wolf haplotypes by at least two substitutions, while D2140 completely matches domestic dog and a wolf sequence in GenBank. This dataset is made available under the Open Database License (http://opendatacommons.org/licenses/odbl/1.0/). The Open Database License (ODbL) is a license agreement intended to allow users to freely share, modify, and use this Dataset while maintaining this same freedom for others, provided that the original source and author(s) are credited.Madhu Chetri, Yadvendradev V. Jhala, Shant R. Jnawali, Naresh Subedi, Maheshwar Dhakal, Bibek Yumnam

Supplementary material 4 Median-joining networks of Himalayan wolf and related wolf and dog clades Data type: TIF file Explanation note: Median-joining networks of Himalayan wolf and related wolf and dog clades. Golden jackal and Ethiopian wolf haplotypes are shown for comparison. Circle size and branches are proportional to sampled haplotype frequency and number of nucleotide mutation steps among haplotypes, respectively. Branch numbers refer to mutation steps separating individual haplotypes. Scat samples sequenced in this study are represented by arrows falling within the Himalayan wolf (HWF) and Indian feral dog (IDH) haplotypes. Nomenclature for the African wolf follows Koepfli et al. (2015). This dataset is made available under the Open Database License (http://opendatacommons.org/licenses/odbl/1.0/). The Open Database License (ODbL) is a license agreement intended to allow users to freely share, modify, and use this Dataset while maintaining this same freedom for others, provided that the original source and author(s) are credited.Madhu Chetri, Yadvendradev V. Jhala, Shant R. Jnawali, Naresh Subedi, Maheshwar Dhakal, Bibek Yumnam

Supplementary material 5 Blast report of sample D2140 Data type: PDF file Explanation note: Blast search result for D2140 scat sequence. This dataset is made available under the Open Database License (http://opendatacommons.org/licenses/odbl/1.0/). The Open Database License (ODbL) is a license agreement intended to allow users to freely share, modify, and use this Dataset while maintaining this same freedom for others, provided that the original source and author(s) are credited.Madhu Chetri, Yadvendradev V. Jhala, Shant R. Jnawali, Naresh Subedi, Maheshwar Dhakal, Bibek Yumnam
