# Supplementary material for: Ancient Himalayan wolf (Canis lupus chanco) lineage in Upper Mustang of the Annapurna Conservation Area, Nepal
Source: Zookeys. 2016 Apr 21;(582):143–56. doi: 10.3897/zookeys.582.5966 (PMC4857050; doi:10.3897/zookeys.582.5966)
Supplement: Supplementary material 1 — Best model selection for Bayesian analysis [file zookeys-582-143-s001.doc]

**Supplementary material 1.** **Best model selection.** Estimating the best model of molecular substitution inferred using Log Bayes Factors (LBF) from Bayesian posterior distributions in Mr Bayes. Rate variation for tested models - gamma distributed rate variation across sites (gamma); gamma distributed with proportion of invariable sites (invgamma); rate variation with proportion of invariable sites (propinv); equal rate variation across sites (equal).

| **Model** | **Harmonic Mean** | **LBF** | **Model probability** |
| --- | --- | --- | --- |
| GTR + invgamma | -3080.46 | 0 | 0.873 |
| GTR + propinv | -3081.537 | -2.154 | 0.101 |
| GTR + gamma | -3095.491 | -30.062 | 0 |
| GTR + equal | -3219.174 | -277.428 | 0 |
| F81 + invgamma | -3445.05 | -729.18 | 0 |
| F81 + propinv | -3411.528 | -662.136 | 0 |
| F81 + gamma | -3443.529 | -726.138 | 0 |
| F81 + equal | -3513.691 | -866.462 | 0 |
| HKY + invgamma | -3123.014 | -85.108 | 0 |
| HKY + propinv | -3082.225 | -3.53 | 0.026 |
| HKY + gamma | -3092.677 | -24.434 | 0 |
| HKY + equal | -3205.015 | -249.11 | 0 |
| mixed + invgamma | -3096.089 | -31.258 | 0 |
| mixed + propinv | -3086.602 | -12.284 | 0 |
| mixed + gamma | -3089.702 | -18.484 | 0 |
| mixed + equal | -3205.862 | -250.804 | 0 |
