# Supplementary material for: Ancient Himalayan wolf (Canis lupus chanco) lineage in Upper Mustang of the Annapurna Conservation Area, Nepal
Source: Zookeys. 2016 Apr 21;(582):143–56. doi: 10.3897/zookeys.582.5966 (PMC4857050; doi:10.3897/zookeys.582.5966)
Supplement: Supplementary material 2 — Genebank sequences analysed in this study [file zookeys-582-143-s002.doc]

**Supplementary material 2.** Genbank accession numbers for sequences analyzed in this study.

| **Taxon** | **Clade** | **Genbank Accession numbers** |
| --- | --- | --- |
| *Canis lupus chanco* | Himalayan wolf | HWA (AY333738), HWB (AY333739), HWC (AY333740), HWD (AY333741), HWE (AY333742) |
| *Canis lupus chanco* | Himalayan wolf | QH8 (KC414576) |
| *Canis lupus chanco* | wolf-dog clade | China-XJ2 (KC414578), China-NM5 (KC414568), Mongolia-wo252 (AB480744), Mongolia (AB007378), Russia-wo251 (AB480743) |
| *Canis lupus pallipes* | Indian wolf clade | IWA (AY333743), IWB (AY333744), IWC (AY333745), IWD (AY333746) |
| *Canis lupus pallipes* | wolf-dog clade | Saudi Arabia (DQ480506) |
| *Canis lupus familiaris* | Indian dog clades | IDA (AY333727), IDB (AY333728), IDC (AY333729), IDD (AY333730), IDE (AY333731), IDF (AY333732), IDG (AY333733), IDH (AY333734), IDI (AY333735), IDJ (AY333736), IDK (AY333737) |
| *Canis lupus familiaris* | Domestic dogs | Belgium (KM201268), Czechoslovakia (KJ776749), IranA15 (HQ261490), Iran D14 (KC540940), Tibetan Mastiff-H3 (KJ934225), Tibetan Mastif-H8 (KJ934230) |
| *Canis lupus* | Gray wolf | Canada (DQ480508), Sweden (DQ480504), Japan (AB480742), China (KF857179), Iran (KC540917) |
| *Canis lupus* | Gray wolf | Italy-W1 (AF338303), Italy W2(AF338304), Italy-W3 (AF338305), Italy-W4 (AF338306), Italy W5 (AF338307), Italy-W6 (AF338308), Italy-W7 (AF338309), Italy-W8 (AF338310), Italy-W9 (AF338311), Italy-W10 (AF338312) |
| *Canis lupus lupaster* | African wolf | Ethiopia (HQ845259), Mali (JQ088684), Senegal (JQ088683) |
| *Canis aureus* | Golden Jackal | Serbia (HQ845260), India (AY289997) |
| *Canis simiensis* | Ethiopian Wolf | HQ845261, CS02 (AY562099), CS03(AY562100) |
| *Chrysocyon brachyurus* | Maned wolf | KJ508409 |
