# Supplementary material for: Ancient Himalayan wolf (Canis lupus chanco) lineage in Upper Mustang of the Annapurna Conservation Area, Nepal
Source: Zookeys. 2016 Apr 21;(582):143–56. doi: 10.3897/zookeys.582.5966 (PMC4857050; doi:10.3897/zookeys.582.5966)
Supplement: Supplementary material 3 — Aligned CR sequences of selected samples of wolves and dogs from GenBank, with the obtained scat samples [file zookeys-582-143-s003.doc]

**Supplementary material 3.** Aligned CR sequences of selected samples of wolves, dogs from Genbank, with scat samples obtained in this study. Numbers refer to mtDNA nucleotide positions referenced with respect to the complete mtDNA genome of gray wolf (Genbank Accession No. KF857179). Scat sequences D2137, D2138, D2139 and D2143 match each other differs from known Himalayan wolf haplotypes by at least two substitutions, while D2140 completely matches domestic dog and a wolf sequence in Genbank.

15,490 15,500 15,510 15,520 15,530 15,540 15,550 15,560 15,570 15,580 15,590 15,595

....|....|....|....|....|....|....|....|....|....|....|....|....|....|....|....|....|....|....|....|....|....|....|

**chanco_HWC TTGGATCACCTCTACTGTGCTATGTCAGTATCTCCAG--AGATTC----TTCCCTCCCTATGTACGTCGTGCATTAATGGCTTGCCCCATGCATATAAGCATGTACATAATATTA**

**chanco_HWE .....................................--......----...............................T..................................**

**chanco_HWD ......................................--......---....................................................N.............**

**chanco_HWB .....................................--......----......................................T...........................**

**chanco_HWA .....................................--......----..................................................................**

**D2137_contigFrcR ...A......C..........................--......----..................................................................**

**D2138_contig_FrcR ...A......C..........................--......----..................................................................**

**D2139_contigFrcR ...A......C..........................--......----..................................................................**

**D2143_contigFrcR ...A......C..........................--......----..................................................................**

**chanco_KC414578 China** **...A......C..........................GT.A.CC.TTCT.C..TC.........................T..................................**

**chanco_KC414576 China** **.....................................--......----..................................................................**

**chanco_KC414568 China** **...T......C..........................GT.AGCC.TTCT.C..TC.....................G...T..................................**

**chanco_AB480744 Mongol** **...A......C..........................GT.A.CC.TTCT.C..TC.....................G...T..................................**

**chanco_AB007378_Mongol** **...A......C...................C......GT.A.CC.TTCTCC..TC.........................T..................................**

**chanco_AB480743_Russia ...A......C..........................GT.A.CC.TTCT.C..TC.....................G...T..................................**

**pallipes_IWA ...A......C..........................--.A.CC.---T....TC.........................T..................................**

**pallipes_IWB**  **...A......C..........................--.A.CC.---T....TC............................................................**

**pallipes_IWC**  **...A......C..........................--.A.CC.---T....TC.........................T..................................**

**pallipes_IWD**  **...A......C..........................--.A..C.---TC...TC............................................................**

**familiaris_IDA**  **...A......C....................T.....GT.A.CC.TTCT.C..TC.........................T..................................**

**familiaris_IDB**  **...A......C....................T.....GT.A.CC.TTCT.C..TC.........................T..................................**

**familiaris_IDC**  **...A......C..........................GT.A.CC.TTCTCC..TC.........................T..................................**

**familiaris_IDD ...A......C..........................GT.A.CC.TTCT.C..TC.........................T..................................**

**familiaris_IDE ...A......C..........................GT.A.CC.TTCTCC..TC.........................T..................................**

**familiaris_IDF ...A......C..........................GT.A.CC.TTCTCC..TC.........................T..................................**

**familiaris_IDG ...A......C..........................GT.A.CC.TTCTCC..TC.........................T..................................**

**familiaris_IDH ...A......C..........................GT.A.CC.TTCTCC..TC.........................T..................................**

**D2140_contigFrcR ...A......C..........................GT.A.CC.TTCTCC..TC.........................T..................................**

**TibetanMastiff_H3 ...A......C..........................GT.A.CC.TTCTCC..TC.........................T..................................**

**TibetanMastiff_H8 ...A......C..........................GT.A.CC.TTCTCC..TC.........................T..................................**

**familiaris_Iran_D14 ...A......C..........................GT.A.CC.TTCTCC..TC.........................T..................................**

**familiaris_Iran_A15 ...A......C..........................GT.A.CC.TTCTCC..TC.........................T..................................**

**wolf_Iran_W1 ...A......C..........................GT.A.CC.TTCTCC..TC.........................T..................................**

**familiaris_Czech_CSWB ...A......C..........................GT.A.CC.TTCTCC..TC.........................T..................................**

**familiaris_Belgium ...A......C..........................GT.A.CC.TTCTCC..TC.........................T..................................**

**familiaris_IDI ...A......C..........................GT.A.CC.TTCT.C..TC.........................T..................................**

**familiaris_IDJ ...A......C..........................GT.A.CC.TTCTCC..TC.........................T..................................**

**familiaris_IDK ...A......C..........................GT.A.CC.TTCTCC..TC.........................T..................................**

15,600 15,610 15,620 15,630 15,640 15,650 15,660 15,670 15,680 15,690 15,700 15,707

....|....|....|....|....|....|....|....|....|....|....|....|....|....|....|....|....|....|....|....|....|....|..

**chanco_HWC CATTCTTACATAGGACATATCAACTCAACTCCACAATCCACTGAT-TACCAACAGTAATCGAATGCATATCACTTAGTCCAATAAGGGCTTAATCACCATGCCTCGAGAAAC**

**chanco_HWE .............................................-...............G..................................................**

**chanco_HWD ........................................T....-..................................................................**

**chanco_HWB .............................................-.....G.G.....T....................................................**

**chanco_HWA .............................................-...............G..................................................**

**D2137_contigFrcR .............................................--.................................................................**

**D2138_contig_FrcR .............................................-..................................................................**

**D2139_contigFrcR .............................................-..................................................................**

**D2143_contigFrcR .............................................-..................................................................**

**chanco_KC414578 China** **T..C................T.......TCT..T...T..T....C..T..G........A...................................................**

**chanco_KC414576 China .............................................-..................................................................**

**chanco_KC414568 China T..C................T.......TCT..T...T.......C..T...............................................................**

**chanco_AB480744 Mongol T..C................T.......TCT..T...T.......C..T..G........A...................................................**

**chanco_AB007378_Mongol T..C................T.......TCT..T.G.T.......C..T...............................................................**

**chanco_AB480743_Russia T...................T.......TCT..T...T.......C..T..G........A...................................................**

**pallipes_IWA T..C................T.......TCT......T...C...TC.............AG...........C......................................**

**pallipes_IWB T..C................T.......TCT......T...C...TC.............AG...........C......................................**

**pallipes_IWC T..C................T.......TCT......T...C...TC.............A............C......................................**

**pallipes_IWD T..C................T.......TCT......T...C...TC.............AG...........C...............................A......**

**familiaris_IDA T..C...............CT.......TCT..T...T.......C..T.........C.....................................................**

**familiaris_IDB T..C...............CT.......TCT..T...T.......C.GT.........C.....................................................**

**familiaris_IDC T..C................T.......TCT......T..T...CC..T...............................................................**

**familiaris_IDD T...........................TCT..T...T..T....C.GT..G........A...................................................**

**familiaris_IDE T..C................T.......TCT....G.T..T....C..T...........A............C......................................**

**familiaris_IDF T..C................T.......TCT..T...T.......C..T..........G....................................................**

**familiaris_IDG T..C................T.......TCT..T...T.......C..T...........A...................................................**

**familiaris_IDH T..C................T.......TCT..T...T.......C..T...............................................................**

**D2140_contigFrcR T..C................T.......TCT..T...T.......C..T...............................................................**

**TibetanMastiff_H3 T..C................T.......TCT..T...T.......C..T...............................................................**

**TibetanMastiff_H8 T..C................T.......TCT..T...T.......C..T...............................................................**

**familiaris_Iran_D14 T..C................T.......TCT..T...T.......C..T...............................................................**

**familiaris_Iran_A15 T..C................T.......TCT..T...T.......C..T...............................................................**

**wolf_Iran_W1 T..C................T.......TCT..T...T.......C..T...............................................................**

**familiaris_Czech_CSWB T..C................T.......TCT..T...T.......C..T...............................................................**

**familiaris_Belgium T..C................T.......TCT..T...T.......C..T...............................................................**

**familiaris_IDI T...........................TCT..TG..T..T....C.GT..G........A...................................................**

**familiaris_IDJ T..C................T.......TCT..T.G.T.......C..T...........A...................................................**

**familiaris_IDK T..C................T.......TCT..T...T.......C.TT...............................................................**
