# Supplementary figures and images for: Ancient Himalayan wolf (Canis lupus chanco) lineage in Upper Mustang of the Annapurna Conservation Area, Nepal
Source: Zookeys. 2016 Apr 21;(582):143–56. doi: 10.3897/zookeys.582.5966 (PMC4857050; doi:10.3897/zookeys.582.5966)

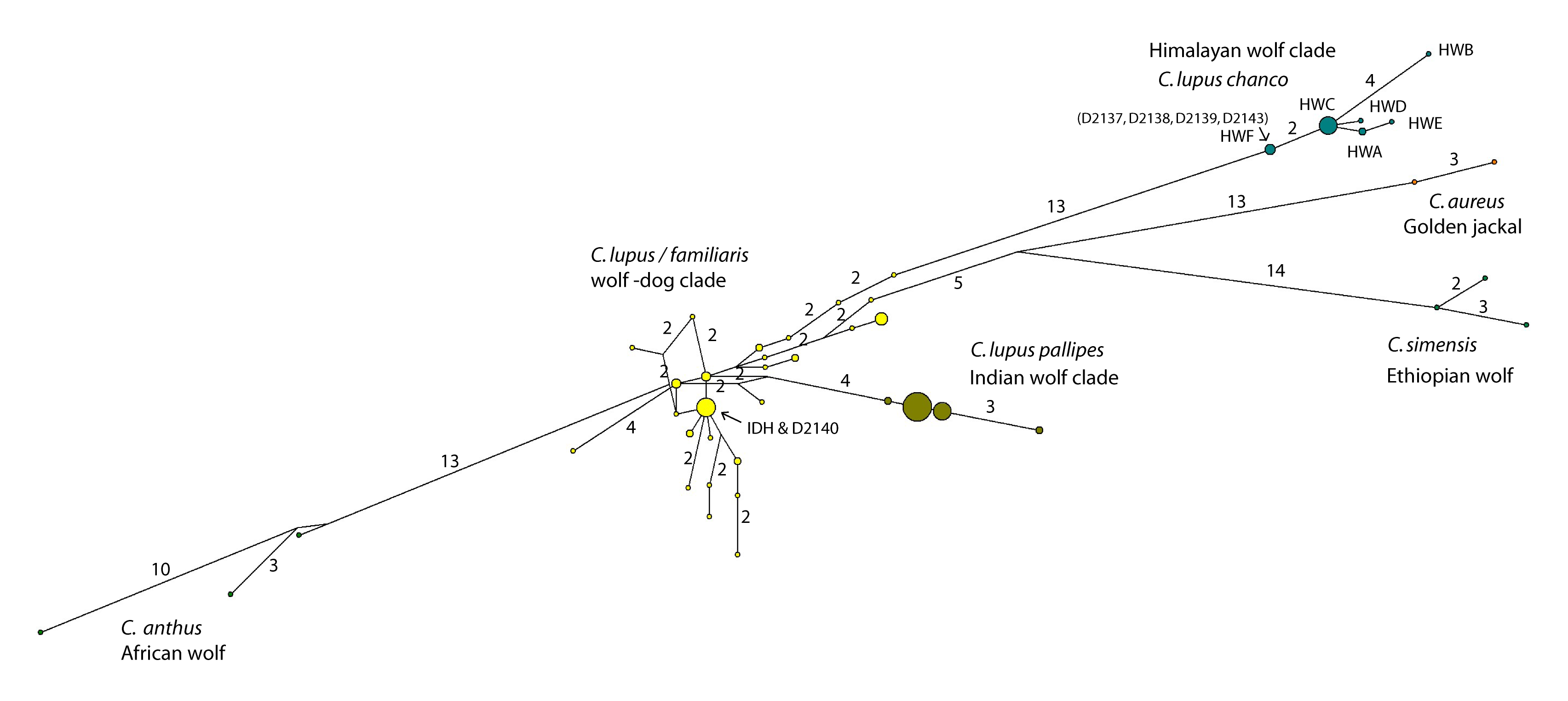

Supplement: Supplementary material 4 — Median-joining networks of Himalayan wolf and related wolf and dog clades [file zookeys-582-143-s004.tif]
