# Supplementary material for: Ancient Himalayan wolf (Canis lupus chanco) lineage in Upper Mustang of the Annapurna Conservation Area, Nepal
Source: Zookeys. 2016 Apr 21;(582):143–56. doi: 10.3897/zookeys.582.5966 (PMC4857050; doi:10.3897/zookeys.582.5966)
Supplement: Supplementary material 5 — Blast report of sample D2140 [file zookeys-582-143-s005.pdf]

Supplementary Material 5 - BLAST search result for D2140 scat sequence

BLASTN 2.2.32+
Reference: Zheng Zhang, Scott Schwartz, Lukas Wagner, and
Webb Miller (2000), "A greedy algorithm for aligning DNA
sequences", J Comput Biol 2000; 7(1-2):203-14.

Reference for database indexing: Aleksandr Morgulis, George
Coulouris, Yan Raytselis, Thomas L. Madden, Richa Agarwala,
Alejandro A. Schaffer (2008), "Database Indexing for
Production MegaBLAST Searches", Bioinformatics 24:1757-1764.

RID: UDRJX1BE01N

Database: Nucleotide collection (nt)
31,070,346 sequences; 100,143,135,861 total letters
Query= D2140_contig_Nepal

Length=227

Sequences producing significant alignments:		Score (Bits)	E Value
gb|KJ776749.1|	Canis lupus familiaris haplotype CSWB D-loop, ...	420	6e-114
gb|KP665928.1|	Canis lupus familiaris isolate 23 D-loop, comp...	420	6e-114
gb|KP665927.1|	Canis lupus familiaris isolate 22 D-loop, comp...	420	6e-114
gb|KP665926.1|	Canis lupus familiaris isolate 21 D-loop, comp...	420	6e-114
gb|KP665925.1|	Canis lupus familiaris isolate 20 D-loop, comp...	420	6e-114
gb|KM061588.1|	Canis lupus familiaris isolate Cf_ws3 mitochon...	420	6e-114
gb|KM061584.1|	Canis lupus familiaris isolate Cf_uno mitochon...	420	6e-114
gb|KM061577.1|	Canis lupus familiaris isolate Cf_tdz5 mitocho...	420	6e-114
gb|KM061575.1|	Canis lupus familiaris isolate Cf_tdz19 mitoch...	420	6e-114
gb|KM061570.1|	Canis lupus familiaris isolate Cf_tdz13 mitoch...	420	6e-114
gb|KM061563.1|	Canis lupus familiaris isolate Cf_stp79 mitoch...	420	6e-114
gb|KM061559.1|	Canis lupus familiaris isolate Cf_stp65 mitoch...	420	6e-114
gb|KM061549.1|	Canis lupus familiaris isolate Cf_psy4 mitocho...	420	6e-114
gb|KM061532.1|	Canis lupus familiaris isolate Cf_pl25 mitocho...	420	6e-114
gb|KM061530.1|	Canis lupus familiaris isolate Cf_pl16 mitocho...	420	6e-114
gb|KM061509.1|	Canis lupus familiaris isolate Cf_L56 mitochon...	420	6e-114
gb|KM061499.1|	Canis lupus familiaris isolate Kos_9 mitochond...	420	6e-114
gb|KM061487.1|	Canis lupus familiaris isolate Cf_jam7 mitocho...	420	6e-114
gb|KM061482.1|	Canis lupus familiaris isolate Cf_jam10 mitoch...	420	6e-114
gb|KM061479.1|	Canis lupus familiaris isolate Cf_cau6 mitocho...	420	6e-114
gb|KJ139084.1|	Canis lupus familiaris isolate 1477_ON1 D-loop...	420	6e-114
gb|KC540940.1|	Canis lupus familiaris haplotype Dog_D14 contr...	420	6e-114
gb|KC540917.1|	Canis lupus haplotype Wolf_W1 control region, ...	420	6e-114
gb|KM201268.1|	Canis lupus familiaris haplotype Be73/19 D-loo...	420	6e-114
gb|KJ934230.1|	Canis lupus familiaris haplotype H8 D-loop, pa...	420	6e-114
gb|KJ934225.1|	Canis lupus familiaris haplotype H3 D-loop, pa...	420	6e-114

```
gb|KJ522809.1|  Canis lupus familiaris mitochondrion, complete...  420     6e-114
gb|KJ637070.1|  Canis lupus familiaris haplotype Be22_1 mitoch...  420     6e-114
gb|KJ637068.1|  Canis lupus familiaris haplotype Be19_9 mitoch...  420     6e-114
gb|KJ637067.1|  Canis lupus familiaris haplotype Be19_8 mitoch...  420     6e-114
gb|KJ637065.1|  Canis lupus familiaris haplotype Be19_6 mitoch...  420     6e-114
gb|KJ637064.1|  Canis lupus familiaris haplotype Be19_5 mitoch...  420     6e-114
gb|KJ637063.1|  Canis lupus familiaris haplotype Be19_4 mitoch...  420     6e-114
gb|KJ637062.1|  Canis lupus familiaris haplotype Be19_3 mitoch...  420     6e-114
gb|KJ637061.1|  Canis lupus familiaris haplotype Be19_2 mitoch...  420     6e-114
gb|KJ637060.1|  Canis lupus familiaris haplotype Be19_14 mitoc...  420     6e-114
gb|KJ637058.1|  Canis lupus familiaris haplotype Be19_13/15 mi...  420     6e-114
gb|KJ637056.1|  Canis lupus familiaris haplotype Be19_12 mitoc...  420     6e-114
gb|KJ637055.1|  Canis lupus familiaris haplotype Be19_10/11 mi...  420     6e-114
gb|KJ637054.1|  Canis lupus familiaris haplotype Be19_10 mitoc...  420     6e-114
gb|KJ637053.1|  Canis lupus familiaris haplotype Be19_1 mitoch...  420     6e-114
gb|KF002337.1|  Canis lupus familiaris isolate Y22 mitochondri...  420     6e-114
gb|KF002336.1|  Canis lupus familiaris isolate Y20 mitochondri...  420     6e-114
gb|KF002306.1|  Canis lupus familiaris isolate m705 mitochondr...  420     6e-114
gb|KF002291.1|  Canis lupus familiaris isolate L8 mitochondrio...  420     6e-114
gb|KF002273.1|  Canis lupus familiaris isolate H64 mitochondri...  420     6e-114
gb|KF002268.1|  Canis lupus familiaris isolate H51 mitochondri...  420     6e-114
gb|KF002266.1|  Canis lupus familiaris isolate H44 mitochondri...  420     6e-114
gb|KF002263.1|  Canis lupus familiaris isolate H3 mitochondrio...  420     6e-114
gb|KF002262.1|  Canis lupus familiaris isolate H22 mitochondri...  420     6e-114
gb|KJ472767.1|  Canis lupus familiaris mitochondrion, complete...  420     6e-114
gb|KF857179.1|  Canis lupus mitochondrion, complete genome         420     6e-114
gb|KF757308.1|  Canis lupus familiaris isolate PQ11 D-loop, pa...  420     6e-114
gb|HQ261490.1|  Canis lupus familiaris haplotype A15 control r...  420     6e-114
gb|HQ452429.1|  Canis lupus familiaris haplotype A189 control ...  420     6e-114
gb|KC985190.1|  Canis lupus familiaris D-loop, partial sequenc...  420     6e-114
gb|JX173682.1|  Canis lupus familiaris isolate CAN1 D-loop, pa...  420     6e-114
gb|JN182095.1|  Canis lupus familiaris isolate greyhound3 cyto...  420     6e-114
gb|JN182116.1|  Canis lupus familiaris isolate husky3 cytochro...  420     6e-114
emb|HE687017.1|  Canis lupus familiaris mitochondrial partial ...  420     6e-114
gb|HQ452468.1|  Canis lupus familiaris haplotype A171 control ...  420     6e-114
gb|HQ452422.1|  Canis lupus familiaris haplotype A175 control ...  420     6e-114
gb|JF342891.1|  Canis lupus familiaris haplotype HV1 A11 mitoc...  420     6e-114
gb|JF342882.1|  Canis lupus familiaris haplotype HV1 A11 mitoc...  420     6e-114
gb|JF342858.1|  Canis lupus familiaris haplotype HV1 A11 mitoc...  420     6e-114
gb|JF342857.1|  Canis lupus familiaris haplotype HV1 A11 mitoc...  420     6e-114
gb|JF342835.1|  Canis lupus familiaris haplotype HV1 A11 mitoc...  420     6e-114
gb|JF342833.1|  Canis lupus familiaris haplotype HV1 A11 mitoc...  420     6e-114
gb|JF342827.1|  Canis lupus familiaris haplotype HV1 A11 mitoc...  420     6e-114
gb|JF342820.1|  Canis lupus familiaris haplotype HV1 A11 mitoc...  420     6e-114
gb|JF342814.1|  Canis lupus familiaris haplotype HV1 A169* mit...  420     6e-114
gb|JF342808.1|  Canis lupus familiaris haplotype HV1 A11 mitoc...  420     6e-114
dbj|AB605594.1|  Canis lupus familiaris mitochondrial gene for...  420     6e-114
dbj|AB605582.1|  Canis lupus familiaris mitochondrial gene for...  420     6e-114
dbj|AB605581.1|  Canis lupus familiaris mitochondrial gene for...  420     6e-114
dbj|AB605575.1|  Canis lupus familiaris mitochondrial gene for...  420     6e-114
dbj|AB605570.1|  Canis lupus familiaris mitochondrial gene for...  420     6e-114
dbj|AB605568.1|  Canis lupus familiaris mitochondrial gene for...  420     6e-114
dbj|AB605558.1|  Canis lupus familiaris mitochondrial gene for...  420     6e-114
```

```
dbj|AB605516.1|  Canis lupus familiaris mitochondrial gene for...  420     6e-114
dbj|AB605492.1|  Canis lupus familiaris mitochondrial gene for...  420     6e-114
gb|HQ452457.1|  Canis lupus familiaris haplotype A226 control ...  420     6e-114
dbj|AB622564.1|  Canis lupus familiaris DNA, hypervariable reg...  420     6e-114
dbj|AB622563.1|  Canis lupus familiaris DNA, hypervariable reg...  420     6e-114
dbj|AB622562.1|  Canis lupus familiaris DNA, hypervariable reg...  420     6e-114
dbj|AB622561.1|  Canis lupus familiaris DNA, hypervariable reg...  420     6e-114
gb|HQ997560.1|  Canis lupus familiaris isolate 231 D-loop, par...  420     6e-114
gb|HQ997531.1|  Canis lupus familiaris isolate 201 D-loop, par...  420     6e-114
gb|HQ997493.1|  Canis lupus familiaris isolate 121 D-loop, par...  420     6e-114
gb|HQ997454.1|  Canis lupus familiaris isolate 052 D-loop, par...  420     6e-114
gb|HQ997424.1|  Canis lupus familiaris isolate 004 D-loop, par...  420     6e-114
gb|HM560932.1|  Canis lupus familiaris haplotype Be61 D-loop, ...  420     6e-114
gb|HQ845274.1|  Canis lupus familiaris isolate K_38 breed Dwar...  420     6e-114
gb|HQ845266.1|  Canis lupus familiaris isolate K_30 breed Chih...  420     6e-114
gb|HQ644192.1|  Canis lupus familiaris isolate 87G ATP synthas...  420     6e-114
gb|HQ644148.1|  Canis lupus familiaris isolate h21M ATP syntha...  420     6e-114
gb|HM561541.1|  Canis lupus familiaris isolate K9_18 breed Fox...  420     6e-114
gb|HM561539.1|  Canis lupus familiaris isolate K9_16 breed Sou...  420     6e-114
gb|AF531667.2|  Canis familiaris isolate A14 control region, p...  420     6e-114
gb|HM560892.1|  Canis lupus familiaris haplotype Be21 D-loop, ...  416     7e-113

ALIGNMENTS
>gb|KJ776749.1| Canis lupus familiaris haplotype CSWB D-loop, partial sequence;
mitochondrial
Length=398

 Score = 420 bits (227),  Expect = 6e-114
 Identities = 227/227 (100%), Gaps = 0/227 (0%)
 Strand=Plus/Minus

Query  1    TTGAATCACCCCTACTGTGCTATGTCAGTATCTCCAGGTAAACCCTTCTCCCCTCCCCTA  60
            ||||||||||||||||||||||||||||||||||||||||||||||||||||||||||||
Sbjct  277  TTGAATCACCCCTACTGTGCTATGTCAGTATCTCCAGGTAAACCCTTCTCCCCTCCCCTA  218

Query  61   TGTACGTCGTGCATTAATGGTTTGCCCCATGCATATAAGCATGTACATAATATTATATCC  120
            ||||||||||||||||||||||||||||||||||||||||||||||||||||||||||||
Sbjct  217  TGTACGTCGTGCATTAATGGTTTGCCCCATGCATATAAGCATGTACATAATATTATATCC  158

Query  121  TTACATAGGACATATTAACTCAATCTCATAATTCACTGATCTATCAACAGTAATCGAATG  180
            ||||||||||||||||||||||||||||||||||||||||||||||||||||||||||||
Sbjct  157  TTACATAGGACATATTAACTCAATCTCATAATTCACTGATCTATCAACAGTAATCGAATG  98

Query  181  CATATCACTTAGTCCAATAAGGGCTTAATCACCATGCCTCGAGAAAC  227
            |||||||||||||||||||||||||||||||||||||||||||||||
Sbjct  97   CATATCACTTAGTCCAATAAGGGCTTAATCACCATGCCTCGAGAAAC  51



>gb|KP665928.1| Canis lupus familiaris isolate 23 D-loop, complete sequence;
mitochondrial
Length=1270

 Score = 420 bits (227),  Expect = 6e-114
```

```
 Identities = 227/227 (100%), Gaps = 0/227 (0%)
 Strand=Plus/Plus

Query  1    TTGAATCACCCCTACTGTGCTATGTCAGTATCTCCAGGTAAACCCTTCTCCCCTCCCCTA  60
            ||||||||||||||||||||||||||||||||||||||||||||||||||||||||||||
Sbjct  20   TTGAATCACCCCTACTGTGCTATGTCAGTATCTCCAGGTAAACCCTTCTCCCCTCCCCTA  79

Query  61   TGTACGTCGTGCATTAATGGTTTGCCCCATGCATATAAGCATGTACATAATATTATATCC  120
            ||||||||||||||||||||||||||||||||||||||||||||||||||||||||||||
Sbjct  80   TGTACGTCGTGCATTAATGGTTTGCCCCATGCATATAAGCATGTACATAATATTATATCC  139

Query  121  TTACATAGGACATATTAACTCAATCTCATAATTCACTGATCTATCAACAGTAATCGAATG  180
            ||||||||||||||||||||||||||||||||||||||||||||||||||||||||||||
Sbjct  140  TTACATAGGACATATTAACTCAATCTCATAATTCACTGATCTATCAACAGTAATCGAATG  199

Query  181  CATATCACTTAGTCCAATAAGGGCTTAATCACCATGCCTCGAGAAAC  227
            |||||||||||||||||||||||||||||||||||||||||||||||
Sbjct  200  CATATCACTTAGTCCAATAAGGGCTTAATCACCATGCCTCGAGAAAC  246


>gb|KP665927.1| Canis lupus familiaris isolate 22 D-loop, complete sequence;
mitochondrial
Length=1270

 Score = 420 bits (227),  Expect = 6e-114
 Identities = 227/227 (100%), Gaps = 0/227 (0%)
 Strand=Plus/Plus

Query  1    TTGAATCACCCCTACTGTGCTATGTCAGTATCTCCAGGTAAACCCTTCTCCCCTCCCCTA  60
            ||||||||||||||||||||||||||||||||||||||||||||||||||||||||||||
Sbjct  20   TTGAATCACCCCTACTGTGCTATGTCAGTATCTCCAGGTAAACCCTTCTCCCCTCCCCTA  79

Query  61   TGTACGTCGTGCATTAATGGTTTGCCCCATGCATATAAGCATGTACATAATATTATATCC  120
            ||||||||||||||||||||||||||||||||||||||||||||||||||||||||||||
Sbjct  80   TGTACGTCGTGCATTAATGGTTTGCCCCATGCATATAAGCATGTACATAATATTATATCC  139

Query  121  TTACATAGGACATATTAACTCAATCTCATAATTCACTGATCTATCAACAGTAATCGAATG  180
            ||||||||||||||||||||||||||||||||||||||||||||||||||||||||||||
Sbjct  140  TTACATAGGACATATTAACTCAATCTCATAATTCACTGATCTATCAACAGTAATCGAATG  199

Query  181  CATATCACTTAGTCCAATAAGGGCTTAATCACCATGCCTCGAGAAAC  227
            |||||||||||||||||||||||||||||||||||||||||||||||
Sbjct  200  CATATCACTTAGTCCAATAAGGGCTTAATCACCATGCCTCGAGAAAC  246


>gb|KP665926.1| Canis lupus familiaris isolate 21 D-loop, complete sequence;
mitochondrial
Length=1270

 Score = 420 bits (227),  Expect = 6e-114
 Identities = 227/227 (100%), Gaps = 0/227 (0%)
 Strand=Plus/Plus
```

```
Query  1    TTGAATCACCCCTACTGTGCTATGTCAGTATCTCCAGGTAAACCCTTCTCCCCTCCCCTA  60
            ||||||||||||||||||||||||||||||||||||||||||||||||||||||||||||
Sbjct  20   TTGAATCACCCCTACTGTGCTATGTCAGTATCTCCAGGTAAACCCTTCTCCCCTCCCCTA  79

Query  61   TGTACGTCGTGCATTAATGGTTTGCCCCATGCATATAAGCATGTACATAATATTATATCC  120
            ||||||||||||||||||||||||||||||||||||||||||||||||||||||||||||
Sbjct  80   TGTACGTCGTGCATTAATGGTTTGCCCCATGCATATAAGCATGTACATAATATTATATCC  139

Query  121  TTACATAGGACATATTAACTCAATCTCATAATTCACTGATCTATCAACAGTAATCGAATG  180
            ||||||||||||||||||||||||||||||||||||||||||||||||||||||||||||
Sbjct  140  TTACATAGGACATATTAACTCAATCTCATAATTCACTGATCTATCAACAGTAATCGAATG  199

Query  181  CATATCACTTAGTCCAATAAGGGCTTAATCACCATGCCTCGAGAAAC  227
            |||||||||||||||||||||||||||||||||||||||||||||||
Sbjct  200  CATATCACTTAGTCCAATAAGGGCTTAATCACCATGCCTCGAGAAAC  246


>gb|KP665925.1| Canis lupus familiaris isolate 20 D-loop, complete sequence;
mitochondrial
Length=1270

 Score = 420 bits (227),  Expect = 6e-114
 Identities = 227/227 (100%), Gaps = 0/227 (0%)
 Strand=Plus/Plus

Query  1    TTGAATCACCCCTACTGTGCTATGTCAGTATCTCCAGGTAAACCCTTCTCCCCTCCCCTA  60
            ||||||||||||||||||||||||||||||||||||||||||||||||||||||||||||
Sbjct  20   TTGAATCACCCCTACTGTGCTATGTCAGTATCTCCAGGTAAACCCTTCTCCCCTCCCCTA  79

Query  61   TGTACGTCGTGCATTAATGGTTTGCCCCATGCATATAAGCATGTACATAATATTATATCC  120
            ||||||||||||||||||||||||||||||||||||||||||||||||||||||||||||
Sbjct  80   TGTACGTCGTGCATTAATGGTTTGCCCCATGCATATAAGCATGTACATAATATTATATCC  139

Query  121  TTACATAGGACATATTAACTCAATCTCATAATTCACTGATCTATCAACAGTAATCGAATG  180
            ||||||||||||||||||||||||||||||||||||||||||||||||||||||||||||
Sbjct  140  TTACATAGGACATATTAACTCAATCTCATAATTCACTGATCTATCAACAGTAATCGAATG  199

Query  181  CATATCACTTAGTCCAATAAGGGCTTAATCACCATGCCTCGAGAAAC  227
            |||||||||||||||||||||||||||||||||||||||||||||||
Sbjct  200  CATATCACTTAGTCCAATAAGGGCTTAATCACCATGCCTCGAGAAAC  246


>gb|KM061588.1| Canis lupus familiaris isolate Cf_ws3 mitochondrion, complete
genome
Length=16710

 Score = 420 bits (227),  Expect = 6e-114
 Identities = 227/227 (100%), Gaps = 0/227 (0%)
 Strand=Plus/Plus

Query  1      TTGAATCACCCCTACTGTGCTATGTCAGTATCTCCAGGTAAACCCTTCTCCCCTCCCCTA  60
              ||||||||||||||||||||||||||||||||||||||||||||||||||||||||||||
Sbjct  15480  TTGAATCACCCCTACTGTGCTATGTCAGTATCTCCAGGTAAACCCTTCTCCCCTCCCCTA  15539
```

```
Query  61     TGTACGTCGTGCATTAATGGTTTGCCCCATGCATATAAGCATGTACATAATATTATATCC  120
              ||||||||||||||||||||||||||||||||||||||||||||||||||||||||||||
Sbjct  15540  TGTACGTCGTGCATTAATGGTTTGCCCCATGCATATAAGCATGTACATAATATTATATCC  15599

Query  121    TTACATAGGACATATTAACTCAATCTCATAATTCACTGATCTATCAACAGTAATCGAATG  180
              ||||||||||||||||||||||||||||||||||||||||||||||||||||||||||||
Sbjct  15600  TTACATAGGACATATTAACTCAATCTCATAATTCACTGATCTATCAACAGTAATCGAATG  15659

Query  181    CATATCACTTAGTCCAATAAGGGCTTAATCACCATGCCTCGAGAAAC  227
              |||||||||||||||||||||||||||||||||||||||||||||||
Sbjct  15660  CATATCACTTAGTCCAATAAGGGCTTAATCACCATGCCTCGAGAAAC  15706


>gb|KM061584.1| Canis lupus familiaris isolate Cf_uno mitochondrion, complete
genome
Length=16730

 Score = 420 bits (227),  Expect = 6e-114
 Identities = 227/227 (100%), Gaps = 0/227 (0%)
 Strand=Plus/Plus

Query  1      TTGAATCACCCCTACTGTGCTATGTCAGTATCTCCAGGTAAACCCTTCTCCCCTCCCCTA  60
              ||||||||||||||||||||||||||||||||||||||||||||||||||||||||||||
Sbjct  15480  TTGAATCACCCCTACTGTGCTATGTCAGTATCTCCAGGTAAACCCTTCTCCCCTCCCCTA  15539

Query  61     TGTACGTCGTGCATTAATGGTTTGCCCCATGCATATAAGCATGTACATAATATTATATCC  120
              ||||||||||||||||||||||||||||||||||||||||||||||||||||||||||||
Sbjct  15540  TGTACGTCGTGCATTAATGGTTTGCCCCATGCATATAAGCATGTACATAATATTATATCC  15599

Query  121    TTACATAGGACATATTAACTCAATCTCATAATTCACTGATCTATCAACAGTAATCGAATG  180
              ||||||||||||||||||||||||||||||||||||||||||||||||||||||||||||
Sbjct  15600  TTACATAGGACATATTAACTCAATCTCATAATTCACTGATCTATCAACAGTAATCGAATG  15659

Query  181    CATATCACTTAGTCCAATAAGGGCTTAATCACCATGCCTCGAGAAAC  227
              |||||||||||||||||||||||||||||||||||||||||||||||
Sbjct  15660  CATATCACTTAGTCCAATAAGGGCTTAATCACCATGCCTCGAGAAAC  15706


>gb|KM061577.1| Canis lupus familiaris isolate Cf_tdz5 mitochondrion, complete
genome
Length=16730

 Score = 420 bits (227),  Expect = 6e-114
 Identities = 227/227 (100%), Gaps = 0/227 (0%)
 Strand=Plus/Plus

Query  1      TTGAATCACCCCTACTGTGCTATGTCAGTATCTCCAGGTAAACCCTTCTCCCCTCCCCTA  60
              ||||||||||||||||||||||||||||||||||||||||||||||||||||||||||||
Sbjct  15480  TTGAATCACCCCTACTGTGCTATGTCAGTATCTCCAGGTAAACCCTTCTCCCCTCCCCTA  15539

Query  61     TGTACGTCGTGCATTAATGGTTTGCCCCATGCATATAAGCATGTACATAATATTATATCC  120
              ||||||||||||||||||||||||||||||||||||||||||||||||||||||||||||
```

```
Sbjct  15540  TGTACGTCGTGCATTAATGGTTTGCCCCATGCATATAAGCATGTACATAATATTATATCC  15599

Query  121    TTACATAGGACATATTAACTCAATCTCATAATTCACTGATCTATCAACAGTAATCGAATG  180
              ||||||||||||||||||||||||||||||||||||||||||||||||||||||||||||
Sbjct  15600  TTACATAGGACATATTAACTCAATCTCATAATTCACTGATCTATCAACAGTAATCGAATG  15659

Query  181    CATATCACTTAGTCCAATAAGGGCTTAATCACCATGCCTCGAGAAAC  227
              |||||||||||||||||||||||||||||||||||||||||||||||
Sbjct  15660  CATATCACTTAGTCCAATAAGGGCTTAATCACCATGCCTCGAGAAAC  15706


>gb|KM061575.1| Canis lupus familiaris isolate Cf_tdz19 mitochondrion, complete
genome
Length=16730

 Score = 420 bits (227),  Expect = 6e-114
 Identities = 227/227 (100%), Gaps = 0/227 (0%)
 Strand=Plus/Plus

Query  1      TTGAATCACCCCTACTGTGCTATGTCAGTATCTCCAGGTAAACCCTTCTCCCCTCCCCTA  60
              ||||||||||||||||||||||||||||||||||||||||||||||||||||||||||||
Sbjct  15480  TTGAATCACCCCTACTGTGCTATGTCAGTATCTCCAGGTAAACCCTTCTCCCCTCCCCTA  15539

Query  61     TGTACGTCGTGCATTAATGGTTTGCCCCATGCATATAAGCATGTACATAATATTATATCC  120
              ||||||||||||||||||||||||||||||||||||||||||||||||||||||||||||
Sbjct  15540  TGTACGTCGTGCATTAATGGTTTGCCCCATGCATATAAGCATGTACATAATATTATATCC  15599

Query  121    TTACATAGGACATATTAACTCAATCTCATAATTCACTGATCTATCAACAGTAATCGAATG  180
              ||||||||||||||||||||||||||||||||||||||||||||||||||||||||||||
Sbjct  15600  TTACATAGGACATATTAACTCAATCTCATAATTCACTGATCTATCAACAGTAATCGAATG  15659

Query  181    CATATCACTTAGTCCAATAAGGGCTTAATCACCATGCCTCGAGAAAC  227
              |||||||||||||||||||||||||||||||||||||||||||||||
Sbjct  15660  CATATCACTTAGTCCAATAAGGGCTTAATCACCATGCCTCGAGAAAC  15706


>gb|KM061570.1| Canis lupus familiaris isolate Cf_tdz13 mitochondrion, complete
genome
Length=16730

 Score = 420 bits (227),  Expect = 6e-114
 Identities = 227/227 (100%), Gaps = 0/227 (0%)
 Strand=Plus/Plus

Query  1      TTGAATCACCCCTACTGTGCTATGTCAGTATCTCCAGGTAAACCCTTCTCCCCTCCCCTA  60
              ||||||||||||||||||||||||||||||||||||||||||||||||||||||||||||
Sbjct  15480  TTGAATCACCCCTACTGTGCTATGTCAGTATCTCCAGGTAAACCCTTCTCCCCTCCCCTA  15539

Query  61     TGTACGTCGTGCATTAATGGTTTGCCCCATGCATATAAGCATGTACATAATATTATATCC  120
              ||||||||||||||||||||||||||||||||||||||||||||||||||||||||||||
Sbjct  15540  TGTACGTCGTGCATTAATGGTTTGCCCCATGCATATAAGCATGTACATAATATTATATCC  15599

Query  121    TTACATAGGACATATTAACTCAATCTCATAATTCACTGATCTATCAACAGTAATCGAATG  180
```

```
             ||||||||||||||||||||||||||||||||||||||||||||||||||||||||||||
Sbjct  15600  TTACATAGGACATATTAACTCAATCTCATAATTCACTGATCTATCAACAGTAATCGAATG  15659

Query  181    CATATCACTTAGTCCAATAAGGGCTTAATCACCATGCCTCGAGAAAC  227
              |||||||||||||||||||||||||||||||||||||||||||||||
Sbjct  15660  CATATCACTTAGTCCAATAAGGGCTTAATCACCATGCCTCGAGAAAC  15706


>gb|KM061563.1| Canis lupus familiaris isolate Cf_stp79 mitochondrion, complete
genome
Length=16730

 Score = 420 bits (227),  Expect = 6e-114
 Identities = 227/227 (100%), Gaps = 0/227 (0%)
 Strand=Plus/Plus

Query  1      TTGAATCACCCCTACTGTGCTATGTCAGTATCTCCAGGTAAACCCTTCTCCCCTCCCCTA  60
              ||||||||||||||||||||||||||||||||||||||||||||||||||||||||||||
Sbjct  15480  TTGAATCACCCCTACTGTGCTATGTCAGTATCTCCAGGTAAACCCTTCTCCCCTCCCCTA  15539

Query  61     TGTACGTCGTGCATTAATGGTTTGCCCCATGCATATAAGCATGTACATAATATTATATCC  120
              ||||||||||||||||||||||||||||||||||||||||||||||||||||||||||||
Sbjct  15540  TGTACGTCGTGCATTAATGGTTTGCCCCATGCATATAAGCATGTACATAATATTATATCC  15599

Query  121    TTACATAGGACATATTAACTCAATCTCATAATTCACTGATCTATCAACAGTAATCGAATG  180
              ||||||||||||||||||||||||||||||||||||||||||||||||||||||||||||
Sbjct  15600  TTACATAGGACATATTAACTCAATCTCATAATTCACTGATCTATCAACAGTAATCGAATG  15659

Query  181    CATATCACTTAGTCCAATAAGGGCTTAATCACCATGCCTCGAGAAAC  227
              |||||||||||||||||||||||||||||||||||||||||||||||
Sbjct  15660  CATATCACTTAGTCCAATAAGGGCTTAATCACCATGCCTCGAGAAAC  15706


>gb|KM061559.1| Canis lupus familiaris isolate Cf_stp65 mitochondrion, complete
genome
Length=16730

 Score = 420 bits (227),  Expect = 6e-114
 Identities = 227/227 (100%), Gaps = 0/227 (0%)
 Strand=Plus/Plus

Query  1      TTGAATCACCCCTACTGTGCTATGTCAGTATCTCCAGGTAAACCCTTCTCCCCTCCCCTA  60
              ||||||||||||||||||||||||||||||||||||||||||||||||||||||||||||
Sbjct  15480  TTGAATCACCCCTACTGTGCTATGTCAGTATCTCCAGGTAAACCCTTCTCCCCTCCCCTA  15539

Query  61     TGTACGTCGTGCATTAATGGTTTGCCCCATGCATATAAGCATGTACATAATATTATATCC  120
              ||||||||||||||||||||||||||||||||||||||||||||||||||||||||||||
Sbjct  15540  TGTACGTCGTGCATTAATGGTTTGCCCCATGCATATAAGCATGTACATAATATTATATCC  15599

Query  121    TTACATAGGACATATTAACTCAATCTCATAATTCACTGATCTATCAACAGTAATCGAATG  180
              ||||||||||||||||||||||||||||||||||||||||||||||||||||||||||||
Sbjct  15600  TTACATAGGACATATTAACTCAATCTCATAATTCACTGATCTATCAACAGTAATCGAATG  15659
```

```
Query  181    CATATCACTTAGTCCAATAAGGGCTTAATCACCATGCCTCGAGAAAC  227
              |||||||||||||||||||||||||||||||||||||||||||||||
Sbjct  15660  CATATCACTTAGTCCAATAAGGGCTTAATCACCATGCCTCGAGAAAC  15706


>gb|KM061549.1| Canis lupus familiaris isolate Cf_psy4 mitochondrion, complete
genome
Length=16730

 Score = 420 bits (227),  Expect = 6e-114
 Identities = 227/227 (100%), Gaps = 0/227 (0%)
 Strand=Plus/Plus

Query  1      TTGAATCACCCCTACTGTGCTATGTCAGTATCTCCAGGTAAACCCTTCTCCCCTCCCCTA  60
              ||||||||||||||||||||||||||||||||||||||||||||||||||||||||||||
Sbjct  15480  TTGAATCACCCCTACTGTGCTATGTCAGTATCTCCAGGTAAACCCTTCTCCCCTCCCCTA  15539

Query  61     TGTACGTCGTGCATTAATGGTTTGCCCCATGCATATAAGCATGTACATAATATTATATCC  120
              ||||||||||||||||||||||||||||||||||||||||||||||||||||||||||||
Sbjct  15540  TGTACGTCGTGCATTAATGGTTTGCCCCATGCATATAAGCATGTACATAATATTATATCC  15599

Query  121    TTACATAGGACATATTAACTCAATCTCATAATTCACTGATCTATCAACAGTAATCGAATG  180
              ||||||||||||||||||||||||||||||||||||||||||||||||||||||||||||
Sbjct  15600  TTACATAGGACATATTAACTCAATCTCATAATTCACTGATCTATCAACAGTAATCGAATG  15659

Query  181    CATATCACTTAGTCCAATAAGGGCTTAATCACCATGCCTCGAGAAAC  227
              |||||||||||||||||||||||||||||||||||||||||||||||
Sbjct  15660  CATATCACTTAGTCCAATAAGGGCTTAATCACCATGCCTCGAGAAAC  15706


>gb|KM061532.1| Canis lupus familiaris isolate Cf_pl25 mitochondrion, complete
genome
Length=16730

 Score = 420 bits (227),  Expect = 6e-114
 Identities = 227/227 (100%), Gaps = 0/227 (0%)
 Strand=Plus/Plus

Query  1      TTGAATCACCCCTACTGTGCTATGTCAGTATCTCCAGGTAAACCCTTCTCCCCTCCCCTA  60
              ||||||||||||||||||||||||||||||||||||||||||||||||||||||||||||
Sbjct  15480  TTGAATCACCCCTACTGTGCTATGTCAGTATCTCCAGGTAAACCCTTCTCCCCTCCCCTA  15539

Query  61     TGTACGTCGTGCATTAATGGTTTGCCCCATGCATATAAGCATGTACATAATATTATATCC  120
              ||||||||||||||||||||||||||||||||||||||||||||||||||||||||||||
Sbjct  15540  TGTACGTCGTGCATTAATGGTTTGCCCCATGCATATAAGCATGTACATAATATTATATCC  15599

Query  121    TTACATAGGACATATTAACTCAATCTCATAATTCACTGATCTATCAACAGTAATCGAATG  180
              ||||||||||||||||||||||||||||||||||||||||||||||||||||||||||||
Sbjct  15600  TTACATAGGACATATTAACTCAATCTCATAATTCACTGATCTATCAACAGTAATCGAATG  15659

Query  181    CATATCACTTAGTCCAATAAGGGCTTAATCACCATGCCTCGAGAAAC  227
              |||||||||||||||||||||||||||||||||||||||||||||||
Sbjct  15660  CATATCACTTAGTCCAATAAGGGCTTAATCACCATGCCTCGAGAAAC  15706
```

```
>gb|KM061530.1| Canis lupus familiaris isolate Cf_pl16 mitochondrion, complete
genome
Length=16730

 Score = 420 bits (227),  Expect = 6e-114
 Identities = 227/227 (100%), Gaps = 0/227 (0%)
 Strand=Plus/Plus

Query  1      TTGAATCACCCCTACTGTGCTATGTCAGTATCTCCAGGTAAACCCTTCTCCCCTCCCCTA  60
              ||||||||||||||||||||||||||||||||||||||||||||||||||||||||||||
Sbjct  15480  TTGAATCACCCCTACTGTGCTATGTCAGTATCTCCAGGTAAACCCTTCTCCCCTCCCCTA  15539

Query  61     TGTACGTCGTGCATTAATGGTTTGCCCCATGCATATAAGCATGTACATAATATTATATCC  120
              ||||||||||||||||||||||||||||||||||||||||||||||||||||||||||||
Sbjct  15540  TGTACGTCGTGCATTAATGGTTTGCCCCATGCATATAAGCATGTACATAATATTATATCC  15599

Query  121    TTACATAGGACATATTAACTCAATCTCATAATTCACTGATCTATCAACAGTAATCGAATG  180
              ||||||||||||||||||||||||||||||||||||||||||||||||||||||||||||
Sbjct  15600  TTACATAGGACATATTAACTCAATCTCATAATTCACTGATCTATCAACAGTAATCGAATG  15659

Query  181    CATATCACTTAGTCCAATAAGGGCTTAATCACCATGCCTCGAGAAAC  227
              |||||||||||||||||||||||||||||||||||||||||||||||
Sbjct  15660  CATATCACTTAGTCCAATAAGGGCTTAATCACCATGCCTCGAGAAAC  15706


>gb|KM061509.1| Canis lupus familiaris isolate Cf_L56 mitochondrion, complete
genome
Length=16730

 Score = 420 bits (227),  Expect = 6e-114
 Identities = 227/227 (100%), Gaps = 0/227 (0%)
 Strand=Plus/Plus

Query  1      TTGAATCACCCCTACTGTGCTATGTCAGTATCTCCAGGTAAACCCTTCTCCCCTCCCCTA  60
              ||||||||||||||||||||||||||||||||||||||||||||||||||||||||||||
Sbjct  15480  TTGAATCACCCCTACTGTGCTATGTCAGTATCTCCAGGTAAACCCTTCTCCCCTCCCCTA  15539

Query  61     TGTACGTCGTGCATTAATGGTTTGCCCCATGCATATAAGCATGTACATAATATTATATCC  120
              ||||||||||||||||||||||||||||||||||||||||||||||||||||||||||||
Sbjct  15540  TGTACGTCGTGCATTAATGGTTTGCCCCATGCATATAAGCATGTACATAATATTATATCC  15599

Query  121    TTACATAGGACATATTAACTCAATCTCATAATTCACTGATCTATCAACAGTAATCGAATG  180
              ||||||||||||||||||||||||||||||||||||||||||||||||||||||||||||
Sbjct  15600  TTACATAGGACATATTAACTCAATCTCATAATTCACTGATCTATCAACAGTAATCGAATG  15659

Query  181    CATATCACTTAGTCCAATAAGGGCTTAATCACCATGCCTCGAGAAAC  227
              |||||||||||||||||||||||||||||||||||||||||||||||
Sbjct  15660  CATATCACTTAGTCCAATAAGGGCTTAATCACCATGCCTCGAGAAAC  15706


>gb|KM061499.1| Canis lupus familiaris isolate Kos_9 mitochondrion, complete
```

```
genome
Length=16730

 Score = 420 bits (227),  Expect = 6e-114
 Identities = 227/227 (100%), Gaps = 0/227 (0%)
 Strand=Plus/Plus

Query  1      TTGAATCACCCCTACTGTGCTATGTCAGTATCTCCAGGTAAACCCTTCTCCCCTCCCCTA  60
              ||||||||||||||||||||||||||||||||||||||||||||||||||||||||||||
Sbjct  15480  TTGAATCACCCCTACTGTGCTATGTCAGTATCTCCAGGTAAACCCTTCTCCCCTCCCCTA  15539

Query  61     TGTACGTCGTGCATTAATGGTTTGCCCCATGCATATAAGCATGTACATAATATTATATCC  120
              ||||||||||||||||||||||||||||||||||||||||||||||||||||||||||||
Sbjct  15540  TGTACGTCGTGCATTAATGGTTTGCCCCATGCATATAAGCATGTACATAATATTATATCC  15599

Query  121    TTACATAGGACATATTAACTCAATCTCATAATTCACTGATCTATCAACAGTAATCGAATG  180
              ||||||||||||||||||||||||||||||||||||||||||||||||||||||||||||
Sbjct  15600  TTACATAGGACATATTAACTCAATCTCATAATTCACTGATCTATCAACAGTAATCGAATG  15659

Query  181    CATATCACTTAGTCCAATAAGGGCTTAATCACCATGCCTCGAGAAAC  227
              |||||||||||||||||||||||||||||||||||||||||||||||
Sbjct  15660  CATATCACTTAGTCCAATAAGGGCTTAATCACCATGCCTCGAGAAAC  15706


>gb|KM061487.1| Canis lupus familiaris isolate Cf_jam7 mitochondrion, complete
genome
Length=16730

 Score = 420 bits (227),  Expect = 6e-114
 Identities = 227/227 (100%), Gaps = 0/227 (0%)
 Strand=Plus/Plus

Query  1      TTGAATCACCCCTACTGTGCTATGTCAGTATCTCCAGGTAAACCCTTCTCCCCTCCCCTA  60
              ||||||||||||||||||||||||||||||||||||||||||||||||||||||||||||
Sbjct  15480  TTGAATCACCCCTACTGTGCTATGTCAGTATCTCCAGGTAAACCCTTCTCCCCTCCCCTA  15539

Query  61     TGTACGTCGTGCATTAATGGTTTGCCCCATGCATATAAGCATGTACATAATATTATATCC  120
              ||||||||||||||||||||||||||||||||||||||||||||||||||||||||||||
Sbjct  15540  TGTACGTCGTGCATTAATGGTTTGCCCCATGCATATAAGCATGTACATAATATTATATCC  15599

Query  121    TTACATAGGACATATTAACTCAATCTCATAATTCACTGATCTATCAACAGTAATCGAATG  180
              ||||||||||||||||||||||||||||||||||||||||||||||||||||||||||||
Sbjct  15600  TTACATAGGACATATTAACTCAATCTCATAATTCACTGATCTATCAACAGTAATCGAATG  15659

Query  181    CATATCACTTAGTCCAATAAGGGCTTAATCACCATGCCTCGAGAAAC  227
              |||||||||||||||||||||||||||||||||||||||||||||||
Sbjct  15660  CATATCACTTAGTCCAATAAGGGCTTAATCACCATGCCTCGAGAAAC  15706


>gb|KM061482.1| Canis lupus familiaris isolate Cf_jam10 mitochondrion, complete
genome
Length=16730
```

```
 Score = 420 bits (227),  Expect = 6e-114
 Identities = 227/227 (100%), Gaps = 0/227 (0%)
 Strand=Plus/Plus

Query  1      TTGAATCACCCCTACTGTGCTATGTCAGTATCTCCAGGTAAACCCTTCTCCCCTCCCCTA  60
              ||||||||||||||||||||||||||||||||||||||||||||||||||||||||||||
Sbjct  15480  TTGAATCACCCCTACTGTGCTATGTCAGTATCTCCAGGTAAACCCTTCTCCCCTCCCCTA  15539

Query  61     TGTACGTCGTGCATTAATGGTTTGCCCCATGCATATAAGCATGTACATAATATTATATCC  120
              ||||||||||||||||||||||||||||||||||||||||||||||||||||||||||||
Sbjct  15540  TGTACGTCGTGCATTAATGGTTTGCCCCATGCATATAAGCATGTACATAATATTATATCC  15599

Query  121    TTACATAGGACATATTAACTCAATCTCATAATTCACTGATCTATCAACAGTAATCGAATG  180
              ||||||||||||||||||||||||||||||||||||||||||||||||||||||||||||
Sbjct  15600  TTACATAGGACATATTAACTCAATCTCATAATTCACTGATCTATCAACAGTAATCGAATG  15659

Query  181    CATATCACTTAGTCCAATAAGGGCTTAATCACCATGCCTCGAGAAAC  227
              |||||||||||||||||||||||||||||||||||||||||||||||
Sbjct  15660  CATATCACTTAGTCCAATAAGGGCTTAATCACCATGCCTCGAGAAAC  15706


>gb|KM061479.1| Canis lupus familiaris isolate Cf_cau6 mitochondrion, complete
genome
Length=16730

 Score = 420 bits (227),  Expect = 6e-114
 Identities = 227/227 (100%), Gaps = 0/227 (0%)
 Strand=Plus/Plus

Query  1      TTGAATCACCCCTACTGTGCTATGTCAGTATCTCCAGGTAAACCCTTCTCCCCTCCCCTA  60
              ||||||||||||||||||||||||||||||||||||||||||||||||||||||||||||
Sbjct  15480  TTGAATCACCCCTACTGTGCTATGTCAGTATCTCCAGGTAAACCCTTCTCCCCTCCCCTA  15539

Query  61     TGTACGTCGTGCATTAATGGTTTGCCCCATGCATATAAGCATGTACATAATATTATATCC  120
              ||||||||||||||||||||||||||||||||||||||||||||||||||||||||||||
Sbjct  15540  TGTACGTCGTGCATTAATGGTTTGCCCCATGCATATAAGCATGTACATAATATTATATCC  15599

Query  121    TTACATAGGACATATTAACTCAATCTCATAATTCACTGATCTATCAACAGTAATCGAATG  180
              ||||||||||||||||||||||||||||||||||||||||||||||||||||||||||||
Sbjct  15600  TTACATAGGACATATTAACTCAATCTCATAATTCACTGATCTATCAACAGTAATCGAATG  15659

Query  181    CATATCACTTAGTCCAATAAGGGCTTAATCACCATGCCTCGAGAAAC  227
              |||||||||||||||||||||||||||||||||||||||||||||||
Sbjct  15660  CATATCACTTAGTCCAATAAGGGCTTAATCACCATGCCTCGAGAAAC  15706


>gb|KJ139084.1| Canis lupus familiaris isolate 1477_ON1 D-loop, partial sequence;
mitochondrial
 gb|KJ139085.1| Canis lupus familiaris isolate 1737_ON4 D-loop, partial sequence;
mitochondrial
 gb|KJ139086.1| Canis lupus familiaris isolate 1739_ON3 D-loop, partial sequence;
mitochondrial
Length=582
```

```
 Score = 420 bits (227),  Expect = 6e-114
 Identities = 227/227 (100%), Gaps = 0/227 (0%)
 Strand=Plus/Plus

Query  1    TTGAATCACCCCTACTGTGCTATGTCAGTATCTCCAGGTAAACCCTTCTCCCCTCCCCTA  60
            ||||||||||||||||||||||||||||||||||||||||||||||||||||||||||||
Sbjct  20   TTGAATCACCCCTACTGTGCTATGTCAGTATCTCCAGGTAAACCCTTCTCCCCTCCCCTA  79

Query  61   TGTACGTCGTGCATTAATGGTTTGCCCCATGCATATAAGCATGTACATAATATTATATCC  120
            ||||||||||||||||||||||||||||||||||||||||||||||||||||||||||||
Sbjct  80   TGTACGTCGTGCATTAATGGTTTGCCCCATGCATATAAGCATGTACATAATATTATATCC  139

Query  121  TTACATAGGACATATTAACTCAATCTCATAATTCACTGATCTATCAACAGTAATCGAATG  180
            ||||||||||||||||||||||||||||||||||||||||||||||||||||||||||||
Sbjct  140  TTACATAGGACATATTAACTCAATCTCATAATTCACTGATCTATCAACAGTAATCGAATG  199

Query  181  CATATCACTTAGTCCAATAAGGGCTTAATCACCATGCCTCGAGAAAC  227
            |||||||||||||||||||||||||||||||||||||||||||||||
Sbjct  200  CATATCACTTAGTCCAATAAGGGCTTAATCACCATGCCTCGAGAAAC  246


>gb|KC540940.1| Canis lupus familiaris haplotype Dog_D14 control region, partial
sequence; mitochondrial
Length=536

 Score = 420 bits (227),  Expect = 6e-114
 Identities = 227/227 (100%), Gaps = 0/227 (0%)
 Strand=Plus/Plus

Query  1    TTGAATCACCCCTACTGTGCTATGTCAGTATCTCCAGGTAAACCCTTCTCCCCTCCCCTA  60
            ||||||||||||||||||||||||||||||||||||||||||||||||||||||||||||
Sbjct  20   TTGAATCACCCCTACTGTGCTATGTCAGTATCTCCAGGTAAACCCTTCTCCCCTCCCCTA  79

Query  61   TGTACGTCGTGCATTAATGGTTTGCCCCATGCATATAAGCATGTACATAATATTATATCC  120
            ||||||||||||||||||||||||||||||||||||||||||||||||||||||||||||
Sbjct  80   TGTACGTCGTGCATTAATGGTTTGCCCCATGCATATAAGCATGTACATAATATTATATCC  139

Query  121  TTACATAGGACATATTAACTCAATCTCATAATTCACTGATCTATCAACAGTAATCGAATG  180
            ||||||||||||||||||||||||||||||||||||||||||||||||||||||||||||
Sbjct  140  TTACATAGGACATATTAACTCAATCTCATAATTCACTGATCTATCAACAGTAATCGAATG  199

Query  181  CATATCACTTAGTCCAATAAGGGCTTAATCACCATGCCTCGAGAAAC  227
            |||||||||||||||||||||||||||||||||||||||||||||||
Sbjct  200  CATATCACTTAGTCCAATAAGGGCTTAATCACCATGCCTCGAGAAAC  246


>gb|KC540917.1| Canis lupus haplotype Wolf_W1 control region, partial sequence;
mitochondrial
 gb|KC540933.1| Canis lupus familiaris haplotype Dog_D6 control region, partial
sequence; mitochondrial
Length=536
```

```
 Score = 420 bits (227),  Expect = 6e-114
 Identities = 227/227 (100%), Gaps = 0/227 (0%)
 Strand=Plus/Plus

Query  1    TTGAATCACCCCTACTGTGCTATGTCAGTATCTCCAGGTAAACCCTTCTCCCCTCCCCTA  60
            ||||||||||||||||||||||||||||||||||||||||||||||||||||||||||||
Sbjct  20   TTGAATCACCCCTACTGTGCTATGTCAGTATCTCCAGGTAAACCCTTCTCCCCTCCCCTA  79

Query  61   TGTACGTCGTGCATTAATGGTTTGCCCCATGCATATAAGCATGTACATAATATTATATCC  120
            ||||||||||||||||||||||||||||||||||||||||||||||||||||||||||||
Sbjct  80   TGTACGTCGTGCATTAATGGTTTGCCCCATGCATATAAGCATGTACATAATATTATATCC  139

Query  121  TTACATAGGACATATTAACTCAATCTCATAATTCACTGATCTATCAACAGTAATCGAATG  180
            ||||||||||||||||||||||||||||||||||||||||||||||||||||||||||||
Sbjct  140  TTACATAGGACATATTAACTCAATCTCATAATTCACTGATCTATCAACAGTAATCGAATG  199

Query  181  CATATCACTTAGTCCAATAAGGGCTTAATCACCATGCCTCGAGAAAC  227
            |||||||||||||||||||||||||||||||||||||||||||||||
Sbjct  200  CATATCACTTAGTCCAATAAGGGCTTAATCACCATGCCTCGAGAAAC  246


>gb|KM201268.1| Canis lupus familiaris haplotype Be73/19 D-loop sequence; mitochondrial
Length=1070

 Score = 420 bits (227),  Expect = 6e-114
 Identities = 227/227 (100%), Gaps = 0/227 (0%)
 Strand=Plus/Plus

Query  1    TTGAATCACCCCTACTGTGCTATGTCAGTATCTCCAGGTAAACCCTTCTCCCCTCCCCTA  60
            ||||||||||||||||||||||||||||||||||||||||||||||||||||||||||||
Sbjct  20   TTGAATCACCCCTACTGTGCTATGTCAGTATCTCCAGGTAAACCCTTCTCCCCTCCCCTA  79

Query  61   TGTACGTCGTGCATTAATGGTTTGCCCCATGCATATAAGCATGTACATAATATTATATCC  120
            ||||||||||||||||||||||||||||||||||||||||||||||||||||||||||||
Sbjct  80   TGTACGTCGTGCATTAATGGTTTGCCCCATGCATATAAGCATGTACATAATATTATATCC  139

Query  121  TTACATAGGACATATTAACTCAATCTCATAATTCACTGATCTATCAACAGTAATCGAATG  180
            ||||||||||||||||||||||||||||||||||||||||||||||||||||||||||||
Sbjct  140  TTACATAGGACATATTAACTCAATCTCATAATTCACTGATCTATCAACAGTAATCGAATG  199

Query  181  CATATCACTTAGTCCAATAAGGGCTTAATCACCATGCCTCGAGAAAC  227
            |||||||||||||||||||||||||||||||||||||||||||||||
Sbjct  200  CATATCACTTAGTCCAATAAGGGCTTAATCACCATGCCTCGAGAAAC  246


>gb|KJ934230.1| Canis lupus familiaris haplotype H8 D-loop, partial sequence;
mitochondrial
Length=650

 Score = 420 bits (227),  Expect = 6e-114
 Identities = 227/227 (100%), Gaps = 0/227 (0%)
 Strand=Plus/Plus
```

```
Query  1    TTGAATCACCCCTACTGTGCTATGTCAGTATCTCCAGGTAAACCCTTCTCCCCTCCCCTA  60
            ||||||||||||||||||||||||||||||||||||||||||||||||||||||||||||
Sbjct  20   TTGAATCACCCCTACTGTGCTATGTCAGTATCTCCAGGTAAACCCTTCTCCCCTCCCCTA  79

Query  61   TGTACGTCGTGCATTAATGGTTTGCCCCATGCATATAAGCATGTACATAATATTATATCC  120
            ||||||||||||||||||||||||||||||||||||||||||||||||||||||||||||
Sbjct  80   TGTACGTCGTGCATTAATGGTTTGCCCCATGCATATAAGCATGTACATAATATTATATCC  139

Query  121  TTACATAGGACATATTAACTCAATCTCATAATTCACTGATCTATCAACAGTAATCGAATG  180
            ||||||||||||||||||||||||||||||||||||||||||||||||||||||||||||
Sbjct  140  TTACATAGGACATATTAACTCAATCTCATAATTCACTGATCTATCAACAGTAATCGAATG  199

Query  181  CATATCACTTAGTCCAATAAGGGCTTAATCACCATGCCTCGAGAAAC  227
            |||||||||||||||||||||||||||||||||||||||||||||||
Sbjct  200  CATATCACTTAGTCCAATAAGGGCTTAATCACCATGCCTCGAGAAAC  246


>gb|KJ934225.1| Canis lupus familiaris haplotype H3 D-loop, partial sequence;
mitochondrial
Length=650

 Score = 420 bits (227),  Expect = 6e-114
 Identities = 227/227 (100%), Gaps = 0/227 (0%)
 Strand=Plus/Plus

Query  1    TTGAATCACCCCTACTGTGCTATGTCAGTATCTCCAGGTAAACCCTTCTCCCCTCCCCTA  60
            ||||||||||||||||||||||||||||||||||||||||||||||||||||||||||||
Sbjct  20   TTGAATCACCCCTACTGTGCTATGTCAGTATCTCCAGGTAAACCCTTCTCCCCTCCCCTA  79

Query  61   TGTACGTCGTGCATTAATGGTTTGCCCCATGCATATAAGCATGTACATAATATTATATCC  120
            ||||||||||||||||||||||||||||||||||||||||||||||||||||||||||||
Sbjct  80   TGTACGTCGTGCATTAATGGTTTGCCCCATGCATATAAGCATGTACATAATATTATATCC  139

Query  121  TTACATAGGACATATTAACTCAATCTCATAATTCACTGATCTATCAACAGTAATCGAATG  180
            ||||||||||||||||||||||||||||||||||||||||||||||||||||||||||||
Sbjct  140  TTACATAGGACATATTAACTCAATCTCATAATTCACTGATCTATCAACAGTAATCGAATG  199

Query  181  CATATCACTTAGTCCAATAAGGGCTTAATCACCATGCCTCGAGAAAC  227
            |||||||||||||||||||||||||||||||||||||||||||||||
Sbjct  200  CATATCACTTAGTCCAATAAGGGCTTAATCACCATGCCTCGAGAAAC  246


>gb|KJ522809.1| Canis lupus familiaris mitochondrion, complete genome
Length=16730

 Score = 420 bits (227),  Expect = 6e-114
 Identities = 227/227 (100%), Gaps = 0/227 (0%)
 Strand=Plus/Plus

Query  1      TTGAATCACCCCTACTGTGCTATGTCAGTATCTCCAGGTAAACCCTTCTCCCCTCCCCTA  60
              ||||||||||||||||||||||||||||||||||||||||||||||||||||||||||||
Sbjct  15480  TTGAATCACCCCTACTGTGCTATGTCAGTATCTCCAGGTAAACCCTTCTCCCCTCCCCTA  15539
```

```
Query  61     TGTACGTCGTGCATTAATGGTTTGCCCCATGCATATAAGCATGTACATAATATTATATCC  120
              ||||||||||||||||||||||||||||||||||||||||||||||||||||||||||||
Sbjct  15540  TGTACGTCGTGCATTAATGGTTTGCCCCATGCATATAAGCATGTACATAATATTATATCC  15599

Query  121    TTACATAGGACATATTAACTCAATCTCATAATTCACTGATCTATCAACAGTAATCGAATG  180
              ||||||||||||||||||||||||||||||||||||||||||||||||||||||||||||
Sbjct  15600  TTACATAGGACATATTAACTCAATCTCATAATTCACTGATCTATCAACAGTAATCGAATG  15659

Query  181    CATATCACTTAGTCCAATAAGGGCTTAATCACCATGCCTCGAGAAAC  227
              |||||||||||||||||||||||||||||||||||||||||||||||
Sbjct  15660  CATATCACTTAGTCCAATAAGGGCTTAATCACCATGCCTCGAGAAAC  15706


>gb|KJ637070.1| Canis lupus familiaris haplotype Be22_1 mitochondrion, partial
genome
Length=16530

 Score = 420 bits (227),  Expect = 6e-114
 Identities = 227/227 (100%), Gaps = 0/227 (0%)
 Strand=Plus/Plus

Query  1      TTGAATCACCCCTACTGTGCTATGTCAGTATCTCCAGGTAAACCCTTCTCCCCTCCCCTA  60
              ||||||||||||||||||||||||||||||||||||||||||||||||||||||||||||
Sbjct  15480  TTGAATCACCCCTACTGTGCTATGTCAGTATCTCCAGGTAAACCCTTCTCCCCTCCCCTA  15539

Query  61     TGTACGTCGTGCATTAATGGTTTGCCCCATGCATATAAGCATGTACATAATATTATATCC  120
              ||||||||||||||||||||||||||||||||||||||||||||||||||||||||||||
Sbjct  15540  TGTACGTCGTGCATTAATGGTTTGCCCCATGCATATAAGCATGTACATAATATTATATCC  15599

Query  121    TTACATAGGACATATTAACTCAATCTCATAATTCACTGATCTATCAACAGTAATCGAATG  180
              ||||||||||||||||||||||||||||||||||||||||||||||||||||||||||||
Sbjct  15600  TTACATAGGACATATTAACTCAATCTCATAATTCACTGATCTATCAACAGTAATCGAATG  15659

Query  181    CATATCACTTAGTCCAATAAGGGCTTAATCACCATGCCTCGAGAAAC  227
              |||||||||||||||||||||||||||||||||||||||||||||||
Sbjct  15660  CATATCACTTAGTCCAATAAGGGCTTAATCACCATGCCTCGAGAAAC  15706


>gb|KJ637068.1| Canis lupus familiaris haplotype Be19_9 mitochondrion, partial
genome
Length=16530

 Score = 420 bits (227),  Expect = 6e-114
 Identities = 227/227 (100%), Gaps = 0/227 (0%)
 Strand=Plus/Plus

Query  1      TTGAATCACCCCTACTGTGCTATGTCAGTATCTCCAGGTAAACCCTTCTCCCCTCCCCTA  60
              ||||||||||||||||||||||||||||||||||||||||||||||||||||||||||||
Sbjct  15480  TTGAATCACCCCTACTGTGCTATGTCAGTATCTCCAGGTAAACCCTTCTCCCCTCCCCTA  15539

Query  61     TGTACGTCGTGCATTAATGGTTTGCCCCATGCATATAAGCATGTACATAATATTATATCC  120
              ||||||||||||||||||||||||||||||||||||||||||||||||||||||||||||
Sbjct  15540  TGTACGTCGTGCATTAATGGTTTGCCCCATGCATATAAGCATGTACATAATATTATATCC  15599
```

```
Query  121    TTACATAGGACATATTAACTCAATCTCATAATTCACTGATCTATCAACAGTAATCGAATG  180
              ||||||||||||||||||||||||||||||||||||||||||||||||||||||||||||
Sbjct  15600  TTACATAGGACATATTAACTCAATCTCATAATTCACTGATCTATCAACAGTAATCGAATG  15659

Query  181    CATATCACTTAGTCCAATAAGGGCTTAATCACCATGCCTCGAGAAAC  227
              |||||||||||||||||||||||||||||||||||||||||||||||
Sbjct  15660  CATATCACTTAGTCCAATAAGGGCTTAATCACCATGCCTCGAGAAAC  15706


>gb|KJ637067.1| Canis lupus familiaris haplotype Be19_8 mitochondrion, partial
genome
Length=16530

 Score = 420 bits (227),  Expect = 6e-114
 Identities = 227/227 (100%), Gaps = 0/227 (0%)
 Strand=Plus/Plus

Query  1      TTGAATCACCCCTACTGTGCTATGTCAGTATCTCCAGGTAAACCCTTCTCCCCTCCCCTA  60
              ||||||||||||||||||||||||||||||||||||||||||||||||||||||||||||
Sbjct  15480  TTGAATCACCCCTACTGTGCTATGTCAGTATCTCCAGGTAAACCCTTCTCCCCTCCCCTA  15539

Query  61     TGTACGTCGTGCATTAATGGTTTGCCCCATGCATATAAGCATGTACATAATATTATATCC  120
              ||||||||||||||||||||||||||||||||||||||||||||||||||||||||||||
Sbjct  15540  TGTACGTCGTGCATTAATGGTTTGCCCCATGCATATAAGCATGTACATAATATTATATCC  15599

Query  121    TTACATAGGACATATTAACTCAATCTCATAATTCACTGATCTATCAACAGTAATCGAATG  180
              ||||||||||||||||||||||||||||||||||||||||||||||||||||||||||||
Sbjct  15600  TTACATAGGACATATTAACTCAATCTCATAATTCACTGATCTATCAACAGTAATCGAATG  15659

Query  181    CATATCACTTAGTCCAATAAGGGCTTAATCACCATGCCTCGAGAAAC  227
              |||||||||||||||||||||||||||||||||||||||||||||||
Sbjct  15660  CATATCACTTAGTCCAATAAGGGCTTAATCACCATGCCTCGAGAAAC  15706


>gb|KJ637065.1| Canis lupus familiaris haplotype Be19_6 mitochondrion, partial
genome
Length=16530

 Score = 420 bits (227),  Expect = 6e-114
 Identities = 227/227 (100%), Gaps = 0/227 (0%)
 Strand=Plus/Plus

Query  1      TTGAATCACCCCTACTGTGCTATGTCAGTATCTCCAGGTAAACCCTTCTCCCCTCCCCTA  60
              ||||||||||||||||||||||||||||||||||||||||||||||||||||||||||||
Sbjct  15480  TTGAATCACCCCTACTGTGCTATGTCAGTATCTCCAGGTAAACCCTTCTCCCCTCCCCTA  15539

Query  61     TGTACGTCGTGCATTAATGGTTTGCCCCATGCATATAAGCATGTACATAATATTATATCC  120
              ||||||||||||||||||||||||||||||||||||||||||||||||||||||||||||
Sbjct  15540  TGTACGTCGTGCATTAATGGTTTGCCCCATGCATATAAGCATGTACATAATATTATATCC  15599

Query  121    TTACATAGGACATATTAACTCAATCTCATAATTCACTGATCTATCAACAGTAATCGAATG  180
              ||||||||||||||||||||||||||||||||||||||||||||||||||||||||||||
```

```
Sbjct  15600  TTACATAGGACATATTAACTCAATCTCATAATTCACTGATCTATCAACAGTAATCGAATG  15659

Query  181    CATATCACTTAGTCCAATAAGGGCTTAATCACCATGCCTCGAGAAAC  227
              |||||||||||||||||||||||||||||||||||||||||||||||
Sbjct  15660  CATATCACTTAGTCCAATAAGGGCTTAATCACCATGCCTCGAGAAAC  15706


>gb|KJ637064.1| Canis lupus familiaris haplotype Be19_5 mitochondrion, partial
genome
Length=16530

 Score = 420 bits (227),  Expect = 6e-114
 Identities = 227/227 (100%), Gaps = 0/227 (0%)
 Strand=Plus/Plus

Query  1      TTGAATCACCCCTACTGTGCTATGTCAGTATCTCCAGGTAAACCCTTCTCCCCTCCCCTA  60
              ||||||||||||||||||||||||||||||||||||||||||||||||||||||||||||
Sbjct  15480  TTGAATCACCCCTACTGTGCTATGTCAGTATCTCCAGGTAAACCCTTCTCCCCTCCCCTA  15539

Query  61     TGTACGTCGTGCATTAATGGTTTGCCCCATGCATATAAGCATGTACATAATATTATATCC  120
              ||||||||||||||||||||||||||||||||||||||||||||||||||||||||||||
Sbjct  15540  TGTACGTCGTGCATTAATGGTTTGCCCCATGCATATAAGCATGTACATAATATTATATCC  15599

Query  121    TTACATAGGACATATTAACTCAATCTCATAATTCACTGATCTATCAACAGTAATCGAATG  180
              ||||||||||||||||||||||||||||||||||||||||||||||||||||||||||||
Sbjct  15600  TTACATAGGACATATTAACTCAATCTCATAATTCACTGATCTATCAACAGTAATCGAATG  15659

Query  181    CATATCACTTAGTCCAATAAGGGCTTAATCACCATGCCTCGAGAAAC  227
              |||||||||||||||||||||||||||||||||||||||||||||||
Sbjct  15660  CATATCACTTAGTCCAATAAGGGCTTAATCACCATGCCTCGAGAAAC  15706


>gb|KJ637063.1| Canis lupus familiaris haplotype Be19_4 mitochondrion, partial
genome
Length=16530

 Score = 420 bits (227),  Expect = 6e-114
 Identities = 227/227 (100%), Gaps = 0/227 (0%)
 Strand=Plus/Plus

Query  1      TTGAATCACCCCTACTGTGCTATGTCAGTATCTCCAGGTAAACCCTTCTCCCCTCCCCTA  60
              ||||||||||||||||||||||||||||||||||||||||||||||||||||||||||||
Sbjct  15480  TTGAATCACCCCTACTGTGCTATGTCAGTATCTCCAGGTAAACCCTTCTCCCCTCCCCTA  15539

Query  61     TGTACGTCGTGCATTAATGGTTTGCCCCATGCATATAAGCATGTACATAATATTATATCC  120
              ||||||||||||||||||||||||||||||||||||||||||||||||||||||||||||
Sbjct  15540  TGTACGTCGTGCATTAATGGTTTGCCCCATGCATATAAGCATGTACATAATATTATATCC  15599

Query  121    TTACATAGGACATATTAACTCAATCTCATAATTCACTGATCTATCAACAGTAATCGAATG  180
              ||||||||||||||||||||||||||||||||||||||||||||||||||||||||||||
Sbjct  15600  TTACATAGGACATATTAACTCAATCTCATAATTCACTGATCTATCAACAGTAATCGAATG  15659

Query  181    CATATCACTTAGTCCAATAAGGGCTTAATCACCATGCCTCGAGAAAC  227
```

```
               |||||||||||||||||||||||||||||||||||||||||||||||
Sbjct  15660  CATATCACTTAGTCCAATAAGGGCTTAATCACCATGCCTCGAGAAAC  15706


>gb|KJ637062.1| Canis lupus familiaris haplotype Be19_3 mitochondrion, partial
genome
Length=16529

 Score = 420 bits (227),  Expect = 6e-114
 Identities = 227/227 (100%), Gaps = 0/227 (0%)
 Strand=Plus/Plus

Query  1      TTGAATCACCCCTACTGTGCTATGTCAGTATCTCCAGGTAAACCCTTCTCCCCTCCCCTA  60
              ||||||||||||||||||||||||||||||||||||||||||||||||||||||||||||
Sbjct  15479  TTGAATCACCCCTACTGTGCTATGTCAGTATCTCCAGGTAAACCCTTCTCCCCTCCCCTA  15538

Query  61     TGTACGTCGTGCATTAATGGTTTGCCCCATGCATATAAGCATGTACATAATATTATATCC  120
              ||||||||||||||||||||||||||||||||||||||||||||||||||||||||||||
Sbjct  15539  TGTACGTCGTGCATTAATGGTTTGCCCCATGCATATAAGCATGTACATAATATTATATCC  15598

Query  121    TTACATAGGACATATTAACTCAATCTCATAATTCACTGATCTATCAACAGTAATCGAATG  180
              ||||||||||||||||||||||||||||||||||||||||||||||||||||||||||||
Sbjct  15599  TTACATAGGACATATTAACTCAATCTCATAATTCACTGATCTATCAACAGTAATCGAATG  15658

Query  181    CATATCACTTAGTCCAATAAGGGCTTAATCACCATGCCTCGAGAAAC  227
              |||||||||||||||||||||||||||||||||||||||||||||||
Sbjct  15659  CATATCACTTAGTCCAATAAGGGCTTAATCACCATGCCTCGAGAAAC  15705


>gb|KJ637061.1| Canis lupus familiaris haplotype Be19_2 mitochondrion, partial
genome
Length=16530

 Score = 420 bits (227),  Expect = 6e-114
 Identities = 227/227 (100%), Gaps = 0/227 (0%)
 Strand=Plus/Plus

Query  1      TTGAATCACCCCTACTGTGCTATGTCAGTATCTCCAGGTAAACCCTTCTCCCCTCCCCTA  60
              ||||||||||||||||||||||||||||||||||||||||||||||||||||||||||||
Sbjct  15480  TTGAATCACCCCTACTGTGCTATGTCAGTATCTCCAGGTAAACCCTTCTCCCCTCCCCTA  15539

Query  61     TGTACGTCGTGCATTAATGGTTTGCCCCATGCATATAAGCATGTACATAATATTATATCC  120
              ||||||||||||||||||||||||||||||||||||||||||||||||||||||||||||
Sbjct  15540  TGTACGTCGTGCATTAATGGTTTGCCCCATGCATATAAGCATGTACATAATATTATATCC  15599

Query  121    TTACATAGGACATATTAACTCAATCTCATAATTCACTGATCTATCAACAGTAATCGAATG  180
              ||||||||||||||||||||||||||||||||||||||||||||||||||||||||||||
Sbjct  15600  TTACATAGGACATATTAACTCAATCTCATAATTCACTGATCTATCAACAGTAATCGAATG  15659

Query  181    CATATCACTTAGTCCAATAAGGGCTTAATCACCATGCCTCGAGAAAC  227
              |||||||||||||||||||||||||||||||||||||||||||||||
Sbjct  15660  CATATCACTTAGTCCAATAAGGGCTTAATCACCATGCCTCGAGAAAC  15706
```

```
>gb|KJ637060.1| Canis lupus familiaris haplotype Be19_14 mitochondrion, partial
genome
Length=16530

 Score = 420 bits (227),  Expect = 6e-114
 Identities = 227/227 (100%), Gaps = 0/227 (0%)
 Strand=Plus/Plus

Query  1      TTGAATCACCCCTACTGTGCTATGTCAGTATCTCCAGGTAAACCCTTCTCCCCTCCCCTA  60
              ||||||||||||||||||||||||||||||||||||||||||||||||||||||||||||
Sbjct  15480  TTGAATCACCCCTACTGTGCTATGTCAGTATCTCCAGGTAAACCCTTCTCCCCTCCCCTA  15539

Query  61     TGTACGTCGTGCATTAATGGTTTGCCCCATGCATATAAGCATGTACATAATATTATATCC  120
              ||||||||||||||||||||||||||||||||||||||||||||||||||||||||||||
Sbjct  15540  TGTACGTCGTGCATTAATGGTTTGCCCCATGCATATAAGCATGTACATAATATTATATCC  15599

Query  121    TTACATAGGACATATTAACTCAATCTCATAATTCACTGATCTATCAACAGTAATCGAATG  180
              ||||||||||||||||||||||||||||||||||||||||||||||||||||||||||||
Sbjct  15600  TTACATAGGACATATTAACTCAATCTCATAATTCACTGATCTATCAACAGTAATCGAATG  15659

Query  181    CATATCACTTAGTCCAATAAGGGCTTAATCACCATGCCTCGAGAAAC  227
              |||||||||||||||||||||||||||||||||||||||||||||||
Sbjct  15660  CATATCACTTAGTCCAATAAGGGCTTAATCACCATGCCTCGAGAAAC  15706


>gb|KJ637058.1| Canis lupus familiaris haplotype Be19_13/15 mitochondrion, partial
genome
Length=16530

 Score = 420 bits (227),  Expect = 6e-114
 Identities = 227/227 (100%), Gaps = 0/227 (0%)
 Strand=Plus/Plus

Query  1      TTGAATCACCCCTACTGTGCTATGTCAGTATCTCCAGGTAAACCCTTCTCCCCTCCCCTA  60
              ||||||||||||||||||||||||||||||||||||||||||||||||||||||||||||
Sbjct  15480  TTGAATCACCCCTACTGTGCTATGTCAGTATCTCCAGGTAAACCCTTCTCCCCTCCCCTA  15539

Query  61     TGTACGTCGTGCATTAATGGTTTGCCCCATGCATATAAGCATGTACATAATATTATATCC  120
              ||||||||||||||||||||||||||||||||||||||||||||||||||||||||||||
Sbjct  15540  TGTACGTCGTGCATTAATGGTTTGCCCCATGCATATAAGCATGTACATAATATTATATCC  15599

Query  121    TTACATAGGACATATTAACTCAATCTCATAATTCACTGATCTATCAACAGTAATCGAATG  180
              ||||||||||||||||||||||||||||||||||||||||||||||||||||||||||||
Sbjct  15600  TTACATAGGACATATTAACTCAATCTCATAATTCACTGATCTATCAACAGTAATCGAATG  15659

Query  181    CATATCACTTAGTCCAATAAGGGCTTAATCACCATGCCTCGAGAAAC  227
              |||||||||||||||||||||||||||||||||||||||||||||||
Sbjct  15660  CATATCACTTAGTCCAATAAGGGCTTAATCACCATGCCTCGAGAAAC  15706


>gb|KJ637056.1| Canis lupus familiaris haplotype Be19_12 mitochondrion, partial
genome
```

```
Length=16530

 Score = 420 bits (227),  Expect = 6e-114
 Identities = 227/227 (100%), Gaps = 0/227 (0%)
 Strand=Plus/Plus

Query  1      TTGAATCACCCCTACTGTGCTATGTCAGTATCTCCAGGTAAACCCTTCTCCCCTCCCCTA  60
              ||||||||||||||||||||||||||||||||||||||||||||||||||||||||||||
Sbjct  15480  TTGAATCACCCCTACTGTGCTATGTCAGTATCTCCAGGTAAACCCTTCTCCCCTCCCCTA  15539

Query  61     TGTACGTCGTGCATTAATGGTTTGCCCCATGCATATAAGCATGTACATAATATTATATCC  120
              ||||||||||||||||||||||||||||||||||||||||||||||||||||||||||||
Sbjct  15540  TGTACGTCGTGCATTAATGGTTTGCCCCATGCATATAAGCATGTACATAATATTATATCC  15599

Query  121    TTACATAGGACATATTAACTCAATCTCATAATTCACTGATCTATCAACAGTAATCGAATG  180
              ||||||||||||||||||||||||||||||||||||||||||||||||||||||||||||
Sbjct  15600  TTACATAGGACATATTAACTCAATCTCATAATTCACTGATCTATCAACAGTAATCGAATG  15659

Query  181    CATATCACTTAGTCCAATAAGGGCTTAATCACCATGCCTCGAGAAAC  227
              |||||||||||||||||||||||||||||||||||||||||||||||
Sbjct  15660  CATATCACTTAGTCCAATAAGGGCTTAATCACCATGCCTCGAGAAAC  15706


>gb|KJ637055.1| Canis lupus familiaris haplotype Be19_10/11 mitochondrion, partial
genome
Length=16530

 Score = 420 bits (227),  Expect = 6e-114
 Identities = 227/227 (100%), Gaps = 0/227 (0%)
 Strand=Plus/Plus

Query  1      TTGAATCACCCCTACTGTGCTATGTCAGTATCTCCAGGTAAACCCTTCTCCCCTCCCCTA  60
              ||||||||||||||||||||||||||||||||||||||||||||||||||||||||||||
Sbjct  15480  TTGAATCACCCCTACTGTGCTATGTCAGTATCTCCAGGTAAACCCTTCTCCCCTCCCCTA  15539

Query  61     TGTACGTCGTGCATTAATGGTTTGCCCCATGCATATAAGCATGTACATAATATTATATCC  120
              ||||||||||||||||||||||||||||||||||||||||||||||||||||||||||||
Sbjct  15540  TGTACGTCGTGCATTAATGGTTTGCCCCATGCATATAAGCATGTACATAATATTATATCC  15599

Query  121    TTACATAGGACATATTAACTCAATCTCATAATTCACTGATCTATCAACAGTAATCGAATG  180
              ||||||||||||||||||||||||||||||||||||||||||||||||||||||||||||
Sbjct  15600  TTACATAGGACATATTAACTCAATCTCATAATTCACTGATCTATCAACAGTAATCGAATG  15659

Query  181    CATATCACTTAGTCCAATAAGGGCTTAATCACCATGCCTCGAGAAAC  227
              |||||||||||||||||||||||||||||||||||||||||||||||
Sbjct  15660  CATATCACTTAGTCCAATAAGGGCTTAATCACCATGCCTCGAGAAAC  15706


>gb|KJ637054.1| Canis lupus familiaris haplotype Be19_10 mitochondrion, partial
genome
Length=16530

 Score = 420 bits (227),  Expect = 6e-114
```

```
 Identities = 227/227 (100%), Gaps = 0/227 (0%)
 Strand=Plus/Plus

Query  1      TTGAATCACCCCTACTGTGCTATGTCAGTATCTCCAGGTAAACCCTTCTCCCCTCCCCTA  60
              ||||||||||||||||||||||||||||||||||||||||||||||||||||||||||||
Sbjct  15480  TTGAATCACCCCTACTGTGCTATGTCAGTATCTCCAGGTAAACCCTTCTCCCCTCCCCTA  15539

Query  61     TGTACGTCGTGCATTAATGGTTTGCCCCATGCATATAAGCATGTACATAATATTATATCC  120
              ||||||||||||||||||||||||||||||||||||||||||||||||||||||||||||
Sbjct  15540  TGTACGTCGTGCATTAATGGTTTGCCCCATGCATATAAGCATGTACATAATATTATATCC  15599

Query  121    TTACATAGGACATATTAACTCAATCTCATAATTCACTGATCTATCAACAGTAATCGAATG  180
              ||||||||||||||||||||||||||||||||||||||||||||||||||||||||||||
Sbjct  15600  TTACATAGGACATATTAACTCAATCTCATAATTCACTGATCTATCAACAGTAATCGAATG  15659

Query  181    CATATCACTTAGTCCAATAAGGGCTTAATCACCATGCCTCGAGAAAC  227
              |||||||||||||||||||||||||||||||||||||||||||||||
Sbjct  15660  CATATCACTTAGTCCAATAAGGGCTTAATCACCATGCCTCGAGAAAC  15706


>gb|KJ637053.1| Canis lupus familiaris haplotype Be19_1 mitochondrion, partial
genome
Length=16530

 Score = 420 bits (227),  Expect = 6e-114
 Identities = 227/227 (100%), Gaps = 0/227 (0%)
 Strand=Plus/Plus

Query  1      TTGAATCACCCCTACTGTGCTATGTCAGTATCTCCAGGTAAACCCTTCTCCCCTCCCCTA  60
              ||||||||||||||||||||||||||||||||||||||||||||||||||||||||||||
Sbjct  15480  TTGAATCACCCCTACTGTGCTATGTCAGTATCTCCAGGTAAACCCTTCTCCCCTCCCCTA  15539

Query  61     TGTACGTCGTGCATTAATGGTTTGCCCCATGCATATAAGCATGTACATAATATTATATCC  120
              ||||||||||||||||||||||||||||||||||||||||||||||||||||||||||||
Sbjct  15540  TGTACGTCGTGCATTAATGGTTTGCCCCATGCATATAAGCATGTACATAATATTATATCC  15599

Query  121    TTACATAGGACATATTAACTCAATCTCATAATTCACTGATCTATCAACAGTAATCGAATG  180
              ||||||||||||||||||||||||||||||||||||||||||||||||||||||||||||
Sbjct  15600  TTACATAGGACATATTAACTCAATCTCATAATTCACTGATCTATCAACAGTAATCGAATG  15659

Query  181    CATATCACTTAGTCCAATAAGGGCTTAATCACCATGCCTCGAGAAAC  227
              |||||||||||||||||||||||||||||||||||||||||||||||
Sbjct  15660  CATATCACTTAGTCCAATAAGGGCTTAATCACCATGCCTCGAGAAAC  15706


>gb|KF002337.1| Canis lupus familiaris isolate Y22 mitochondrion, partial genome
Length=8018

 Score = 420 bits (227),  Expect = 6e-114
 Identities = 227/227 (100%), Gaps = 0/227 (0%)
 Strand=Plus/Plus

Query  1     TTGAATCACCCCTACTGTGCTATGTCAGTATCTCCAGGTAAACCCTTCTCCCCTCCCCTA  60
```

```
                ||||||||||||||||||||||||||||||||||||||||||||||||||||||||||||
Sbjct  7456   TTGAATCACCCCTACTGTGCTATGTCAGTATCTCCAGGTAAACCCTTCTCCCCTCCCCTA  7515

Query  61     TGTACGTCGTGCATTAATGGTTTGCCCCATGCATATAAGCATGTACATAATATTATATCC  120
              ||||||||||||||||||||||||||||||||||||||||||||||||||||||||||||
Sbjct  7516   TGTACGTCGTGCATTAATGGTTTGCCCCATGCATATAAGCATGTACATAATATTATATCC  7575

Query  121    TTACATAGGACATATTAACTCAATCTCATAATTCACTGATCTATCAACAGTAATCGAATG  180
              ||||||||||||||||||||||||||||||||||||||||||||||||||||||||||||
Sbjct  7576   TTACATAGGACATATTAACTCAATCTCATAATTCACTGATCTATCAACAGTAATCGAATG  7635

Query  181    CATATCACTTAGTCCAATAAGGGCTTAATCACCATGCCTCGAGAAAC  227
              |||||||||||||||||||||||||||||||||||||||||||||||
Sbjct  7636   CATATCACTTAGTCCAATAAGGGCTTAATCACCATGCCTCGAGAAAC  7682


>gb|KF002336.1| Canis lupus familiaris isolate Y20 mitochondrion, partial genome
Length=8018

 Score = 420 bits (227),  Expect = 6e-114
 Identities = 227/227 (100%), Gaps = 0/227 (0%)
 Strand=Plus/Plus

Query  1      TTGAATCACCCCTACTGTGCTATGTCAGTATCTCCAGGTAAACCCTTCTCCCCTCCCCTA  60
              ||||||||||||||||||||||||||||||||||||||||||||||||||||||||||||
Sbjct  7456   TTGAATCACCCCTACTGTGCTATGTCAGTATCTCCAGGTAAACCCTTCTCCCCTCCCCTA  7515

Query  61     TGTACGTCGTGCATTAATGGTTTGCCCCATGCATATAAGCATGTACATAATATTATATCC  120
              ||||||||||||||||||||||||||||||||||||||||||||||||||||||||||||
Sbjct  7516   TGTACGTCGTGCATTAATGGTTTGCCCCATGCATATAAGCATGTACATAATATTATATCC  7575

Query  121    TTACATAGGACATATTAACTCAATCTCATAATTCACTGATCTATCAACAGTAATCGAATG  180
              ||||||||||||||||||||||||||||||||||||||||||||||||||||||||||||
Sbjct  7576   TTACATAGGACATATTAACTCAATCTCATAATTCACTGATCTATCAACAGTAATCGAATG  7635

Query  181    CATATCACTTAGTCCAATAAGGGCTTAATCACCATGCCTCGAGAAAC  227
              |||||||||||||||||||||||||||||||||||||||||||||||
Sbjct  7636   CATATCACTTAGTCCAATAAGGGCTTAATCACCATGCCTCGAGAAAC  7682


>gb|KF002306.1| Canis lupus familiaris isolate m705 mitochondrion, partial genome
Length=8018

 Score = 420 bits (227),  Expect = 6e-114
 Identities = 227/227 (100%), Gaps = 0/227 (0%)
 Strand=Plus/Plus

Query  1      TTGAATCACCCCTACTGTGCTATGTCAGTATCTCCAGGTAAACCCTTCTCCCCTCCCCTA  60
              ||||||||||||||||||||||||||||||||||||||||||||||||||||||||||||
Sbjct  7456   TTGAATCACCCCTACTGTGCTATGTCAGTATCTCCAGGTAAACCCTTCTCCCCTCCCCTA  7515

Query  61     TGTACGTCGTGCATTAATGGTTTGCCCCATGCATATAAGCATGTACATAATATTATATCC  120
              ||||||||||||||||||||||||||||||||||||||||||||||||||||||||||||
```

```
Sbjct  7516  TGTACGTCGTGCATTAATGGTTTGCCCCATGCATATAAGCATGTACATAATATTATATCC  7575

Query  121   TTACATAGGACATATTAACTCAATCTCATAATTCACTGATCTATCAACAGTAATCGAATG  180
             ||||||||||||||||||||||||||||||||||||||||||||||||||||||||||||
Sbjct  7576  TTACATAGGACATATTAACTCAATCTCATAATTCACTGATCTATCAACAGTAATCGAATG  7635

Query  181   CATATCACTTAGTCCAATAAGGGCTTAATCACCATGCCTCGAGAAAC  227
             |||||||||||||||||||||||||||||||||||||||||||||||
Sbjct  7636  CATATCACTTAGTCCAATAAGGGCTTAATCACCATGCCTCGAGAAAC  7682


>gb|KF002291.1| Canis lupus familiaris isolate L8 mitochondrion, partial genome
Length=8018

 Score = 420 bits (227),  Expect = 6e-114
 Identities = 227/227 (100%), Gaps = 0/227 (0%)
 Strand=Plus/Plus

Query  1     TTGAATCACCCCTACTGTGCTATGTCAGTATCTCCAGGTAAACCCTTCTCCCCTCCCCTA  60
             ||||||||||||||||||||||||||||||||||||||||||||||||||||||||||||
Sbjct  7456  TTGAATCACCCCTACTGTGCTATGTCAGTATCTCCAGGTAAACCCTTCTCCCCTCCCCTA  7515

Query  61    TGTACGTCGTGCATTAATGGTTTGCCCCATGCATATAAGCATGTACATAATATTATATCC  120
             ||||||||||||||||||||||||||||||||||||||||||||||||||||||||||||
Sbjct  7516  TGTACGTCGTGCATTAATGGTTTGCCCCATGCATATAAGCATGTACATAATATTATATCC  7575

Query  121   TTACATAGGACATATTAACTCAATCTCATAATTCACTGATCTATCAACAGTAATCGAATG  180
             ||||||||||||||||||||||||||||||||||||||||||||||||||||||||||||
Sbjct  7576  TTACATAGGACATATTAACTCAATCTCATAATTCACTGATCTATCAACAGTAATCGAATG  7635

Query  181   CATATCACTTAGTCCAATAAGGGCTTAATCACCATGCCTCGAGAAAC  227
             |||||||||||||||||||||||||||||||||||||||||||||||
Sbjct  7636  CATATCACTTAGTCCAATAAGGGCTTAATCACCATGCCTCGAGAAAC  7682


>gb|KF002273.1| Canis lupus familiaris isolate H64 mitochondrion, partial genome
 gb|KF002289.1| Canis lupus familiaris isolate L43 mitochondrion, partial genome
Length=8018

 Score = 420 bits (227),  Expect = 6e-114
 Identities = 227/227 (100%), Gaps = 0/227 (0%)
 Strand=Plus/Plus

Query  1     TTGAATCACCCCTACTGTGCTATGTCAGTATCTCCAGGTAAACCCTTCTCCCCTCCCCTA  60
             ||||||||||||||||||||||||||||||||||||||||||||||||||||||||||||
Sbjct  7456  TTGAATCACCCCTACTGTGCTATGTCAGTATCTCCAGGTAAACCCTTCTCCCCTCCCCTA  7515

Query  61    TGTACGTCGTGCATTAATGGTTTGCCCCATGCATATAAGCATGTACATAATATTATATCC  120
             ||||||||||||||||||||||||||||||||||||||||||||||||||||||||||||
Sbjct  7516  TGTACGTCGTGCATTAATGGTTTGCCCCATGCATATAAGCATGTACATAATATTATATCC  7575

Query  121   TTACATAGGACATATTAACTCAATCTCATAATTCACTGATCTATCAACAGTAATCGAATG  180
             ||||||||||||||||||||||||||||||||||||||||||||||||||||||||||||
```

```
Sbjct  7576  TTACATAGGACATATTAACTCAATCTCATAATTCACTGATCTATCAACAGTAATCGAATG  7635

Query  181   CATATCACTTAGTCCAATAAGGGCTTAATCACCATGCCTCGAGAAAC  227
             |||||||||||||||||||||||||||||||||||||||||||||||
Sbjct  7636  CATATCACTTAGTCCAATAAGGGCTTAATCACCATGCCTCGAGAAAC  7682



>gb|KF002268.1| Canis lupus familiaris isolate H51 mitochondrion, partial genome
 gb|KF002277.1| Canis lupus familiaris isolate H85 mitochondrion, partial genome
 gb|KF002288.1| Canis lupus familiaris isolate L36 mitochondrion, partial genome
 gb|KF002297.1| Canis lupus familiaris isolate m432 mitochondrion, partial genome
 gb|KF002313.1| Canis lupus familiaris isolate m752 mitochondrion, partial genome
 gb|KF002326.1| Canis lupus familiaris isolate R41 mitochondrion, partial genome
 gb|KF002327.1| Canis lupus familiaris isolate R42 mitochondrion, partial genome
Length=8018

 Score = 420 bits (227),  Expect = 6e-114
 Identities = 227/227 (100%), Gaps = 0/227 (0%)
 Strand=Plus/Plus

Query  1     TTGAATCACCCCTACTGTGCTATGTCAGTATCTCCAGGTAAACCCTTCTCCCCTCCCCTA  60
             ||||||||||||||||||||||||||||||||||||||||||||||||||||||||||||
Sbjct  7456  TTGAATCACCCCTACTGTGCTATGTCAGTATCTCCAGGTAAACCCTTCTCCCCTCCCCTA  7515

Query  61    TGTACGTCGTGCATTAATGGTTTGCCCCATGCATATAAGCATGTACATAATATTATATCC  120
             ||||||||||||||||||||||||||||||||||||||||||||||||||||||||||||
Sbjct  7516  TGTACGTCGTGCATTAATGGTTTGCCCCATGCATATAAGCATGTACATAATATTATATCC  7575

Query  121   TTACATAGGACATATTAACTCAATCTCATAATTCACTGATCTATCAACAGTAATCGAATG  180
             ||||||||||||||||||||||||||||||||||||||||||||||||||||||||||||
Sbjct  7576  TTACATAGGACATATTAACTCAATCTCATAATTCACTGATCTATCAACAGTAATCGAATG  7635

Query  181   CATATCACTTAGTCCAATAAGGGCTTAATCACCATGCCTCGAGAAAC  227
             |||||||||||||||||||||||||||||||||||||||||||||||
Sbjct  7636  CATATCACTTAGTCCAATAAGGGCTTAATCACCATGCCTCGAGAAAC  7682



>gb|KF002266.1| Canis lupus familiaris isolate H44 mitochondrion, partial genome
 gb|KF002279.1| Canis lupus familiaris isolate H94 mitochondrion, partial genome
 gb|KF002285.1| Canis lupus familiaris isolate L22 mitochondrion, partial genome
 gb|KF002316.1| Canis lupus familiaris isolate Ny78 mitochondrion, partial genome
Length=8018

 Score = 420 bits (227),  Expect = 6e-114
 Identities = 227/227 (100%), Gaps = 0/227 (0%)
 Strand=Plus/Plus

Query  1     TTGAATCACCCCTACTGTGCTATGTCAGTATCTCCAGGTAAACCCTTCTCCCCTCCCCTA  60
             ||||||||||||||||||||||||||||||||||||||||||||||||||||||||||||
Sbjct  7456  TTGAATCACCCCTACTGTGCTATGTCAGTATCTCCAGGTAAACCCTTCTCCCCTCCCCTA  7515

Query  61    TGTACGTCGTGCATTAATGGTTTGCCCCATGCATATAAGCATGTACATAATATTATATCC  120
             ||||||||||||||||||||||||||||||||||||||||||||||||||||||||||||
```

```
Sbjct  7516  TGTACGTCGTGCATTAATGGTTTGCCCCATGCATATAAGCATGTACATAATATTATATCC  7575

Query  121   TTACATAGGACATATTAACTCAATCTCATAATTCACTGATCTATCAACAGTAATCGAATG  180
             ||||||||||||||||||||||||||||||||||||||||||||||||||||||||||||
Sbjct  7576  TTACATAGGACATATTAACTCAATCTCATAATTCACTGATCTATCAACAGTAATCGAATG  7635

Query  181   CATATCACTTAGTCCAATAAGGGCTTAATCACCATGCCTCGAGAAAC  227
             |||||||||||||||||||||||||||||||||||||||||||||||
Sbjct  7636  CATATCACTTAGTCCAATAAGGGCTTAATCACCATGCCTCGAGAAAC  7682


>gb|KF002263.1| Canis lupus familiaris isolate H3 mitochondrion, partial genome
Length=8018

 Score = 420 bits (227),  Expect = 6e-114
 Identities = 227/227 (100%), Gaps = 0/227 (0%)
 Strand=Plus/Plus

Query  1     TTGAATCACCCCTACTGTGCTATGTCAGTATCTCCAGGTAAACCCTTCTCCCCTCCCCTA  60
             ||||||||||||||||||||||||||||||||||||||||||||||||||||||||||||
Sbjct  7456  TTGAATCACCCCTACTGTGCTATGTCAGTATCTCCAGGTAAACCCTTCTCCCCTCCCCTA  7515

Query  61    TGTACGTCGTGCATTAATGGTTTGCCCCATGCATATAAGCATGTACATAATATTATATCC  120
             ||||||||||||||||||||||||||||||||||||||||||||||||||||||||||||
Sbjct  7516  TGTACGTCGTGCATTAATGGTTTGCCCCATGCATATAAGCATGTACATAATATTATATCC  7575

Query  121   TTACATAGGACATATTAACTCAATCTCATAATTCACTGATCTATCAACAGTAATCGAATG  180
             ||||||||||||||||||||||||||||||||||||||||||||||||||||||||||||
Sbjct  7576  TTACATAGGACATATTAACTCAATCTCATAATTCACTGATCTATCAACAGTAATCGAATG  7635

Query  181   CATATCACTTAGTCCAATAAGGGCTTAATCACCATGCCTCGAGAAAC  227
             |||||||||||||||||||||||||||||||||||||||||||||||
Sbjct  7636  CATATCACTTAGTCCAATAAGGGCTTAATCACCATGCCTCGAGAAAC  7682


>gb|KF002262.1| Canis lupus familiaris isolate H22 mitochondrion, partial genome
Length=8018

 Score = 420 bits (227),  Expect = 6e-114
 Identities = 227/227 (100%), Gaps = 0/227 (0%)
 Strand=Plus/Plus

Query  1     TTGAATCACCCCTACTGTGCTATGTCAGTATCTCCAGGTAAACCCTTCTCCCCTCCCCTA  60
             ||||||||||||||||||||||||||||||||||||||||||||||||||||||||||||
Sbjct  7456  TTGAATCACCCCTACTGTGCTATGTCAGTATCTCCAGGTAAACCCTTCTCCCCTCCCCTA  7515

Query  61    TGTACGTCGTGCATTAATGGTTTGCCCCATGCATATAAGCATGTACATAATATTATATCC  120
             ||||||||||||||||||||||||||||||||||||||||||||||||||||||||||||
Sbjct  7516  TGTACGTCGTGCATTAATGGTTTGCCCCATGCATATAAGCATGTACATAATATTATATCC  7575

Query  121   TTACATAGGACATATTAACTCAATCTCATAATTCACTGATCTATCAACAGTAATCGAATG  180
             ||||||||||||||||||||||||||||||||||||||||||||||||||||||||||||
Sbjct  7576  TTACATAGGACATATTAACTCAATCTCATAATTCACTGATCTATCAACAGTAATCGAATG  7635
```

```
Query  181   CATATCACTTAGTCCAATAAGGGCTTAATCACCATGCCTCGAGAAAC  227
             |||||||||||||||||||||||||||||||||||||||||||||||
Sbjct  7636  CATATCACTTAGTCCAATAAGGGCTTAATCACCATGCCTCGAGAAAC  7682


>gb|KJ472767.1| Canis lupus familiaris mitochondrion, complete genome
Length=16730

 Score = 420 bits (227),  Expect = 6e-114
 Identities = 227/227 (100%), Gaps = 0/227 (0%)
 Strand=Plus/Plus

Query  1      TTGAATCACCCCTACTGTGCTATGTCAGTATCTCCAGGTAAACCCTTCTCCCCTCCCCTA  60
              ||||||||||||||||||||||||||||||||||||||||||||||||||||||||||||
Sbjct  15480  TTGAATCACCCCTACTGTGCTATGTCAGTATCTCCAGGTAAACCCTTCTCCCCTCCCCTA  15539

Query  61     TGTACGTCGTGCATTAATGGTTTGCCCCATGCATATAAGCATGTACATAATATTATATCC  120
              ||||||||||||||||||||||||||||||||||||||||||||||||||||||||||||
Sbjct  15540  TGTACGTCGTGCATTAATGGTTTGCCCCATGCATATAAGCATGTACATAATATTATATCC  15599

Query  121    TTACATAGGACATATTAACTCAATCTCATAATTCACTGATCTATCAACAGTAATCGAATG  180
              ||||||||||||||||||||||||||||||||||||||||||||||||||||||||||||
Sbjct  15600  TTACATAGGACATATTAACTCAATCTCATAATTCACTGATCTATCAACAGTAATCGAATG  15659

Query  181    CATATCACTTAGTCCAATAAGGGCTTAATCACCATGCCTCGAGAAAC  227
              |||||||||||||||||||||||||||||||||||||||||||||||
Sbjct  15660  CATATCACTTAGTCCAATAAGGGCTTAATCACCATGCCTCGAGAAAC  15706


>gb|KF857179.1| Canis lupus mitochondrion, complete genome
Length=16729

 Score = 420 bits (227),  Expect = 6e-114
 Identities = 227/227 (100%), Gaps = 0/227 (0%)
 Strand=Plus/Plus

Query  1      TTGAATCACCCCTACTGTGCTATGTCAGTATCTCCAGGTAAACCCTTCTCCCCTCCCCTA  60
              ||||||||||||||||||||||||||||||||||||||||||||||||||||||||||||
Sbjct  15480  TTGAATCACCCCTACTGTGCTATGTCAGTATCTCCAGGTAAACCCTTCTCCCCTCCCCTA  15539

Query  61     TGTACGTCGTGCATTAATGGTTTGCCCCATGCATATAAGCATGTACATAATATTATATCC  120
              ||||||||||||||||||||||||||||||||||||||||||||||||||||||||||||
Sbjct  15540  TGTACGTCGTGCATTAATGGTTTGCCCCATGCATATAAGCATGTACATAATATTATATCC  15599

Query  121    TTACATAGGACATATTAACTCAATCTCATAATTCACTGATCTATCAACAGTAATCGAATG  180
              ||||||||||||||||||||||||||||||||||||||||||||||||||||||||||||
Sbjct  15600  TTACATAGGACATATTAACTCAATCTCATAATTCACTGATCTATCAACAGTAATCGAATG  15659

Query  181    CATATCACTTAGTCCAATAAGGGCTTAATCACCATGCCTCGAGAAAC  227
              |||||||||||||||||||||||||||||||||||||||||||||||
Sbjct  15660  CATATCACTTAGTCCAATAAGGGCTTAATCACCATGCCTCGAGAAAC  15706
```

```
>gb|KF757308.1| Canis lupus familiaris isolate PQ11 D-loop, partial sequence;
mitochondrial
Length=582

 Score = 420 bits (227),  Expect = 6e-114
 Identities = 227/227 (100%), Gaps = 0/227 (0%)
 Strand=Plus/Plus

Query  1    TTGAATCACCCCTACTGTGCTATGTCAGTATCTCCAGGTAAACCCTTCTCCCCTCCCCTA  60
            ||||||||||||||||||||||||||||||||||||||||||||||||||||||||||||
Sbjct  20   TTGAATCACCCCTACTGTGCTATGTCAGTATCTCCAGGTAAACCCTTCTCCCCTCCCCTA  79

Query  61   TGTACGTCGTGCATTAATGGTTTGCCCCATGCATATAAGCATGTACATAATATTATATCC  120
            ||||||||||||||||||||||||||||||||||||||||||||||||||||||||||||
Sbjct  80   TGTACGTCGTGCATTAATGGTTTGCCCCATGCATATAAGCATGTACATAATATTATATCC  139

Query  121  TTACATAGGACATATTAACTCAATCTCATAATTCACTGATCTATCAACAGTAATCGAATG  180
            ||||||||||||||||||||||||||||||||||||||||||||||||||||||||||||
Sbjct  140  TTACATAGGACATATTAACTCAATCTCATAATTCACTGATCTATCAACAGTAATCGAATG  199

Query  181  CATATCACTTAGTCCAATAAGGGCTTAATCACCATGCCTCGAGAAAC  227
            |||||||||||||||||||||||||||||||||||||||||||||||
Sbjct  200  CATATCACTTAGTCCAATAAGGGCTTAATCACCATGCCTCGAGAAAC  246


>gb|HQ261490.1| Canis lupus familiaris haplotype A15 control region, partial
sequence; mitochondrial
Length=610

 Score = 420 bits (227),  Expect = 6e-114
 Identities = 227/227 (100%), Gaps = 0/227 (0%)
 Strand=Plus/Plus

Query  1    TTGAATCACCCCTACTGTGCTATGTCAGTATCTCCAGGTAAACCCTTCTCCCCTCCCCTA  60
            ||||||||||||||||||||||||||||||||||||||||||||||||||||||||||||
Sbjct  48   TTGAATCACCCCTACTGTGCTATGTCAGTATCTCCAGGTAAACCCTTCTCCCCTCCCCTA  107

Query  61   TGTACGTCGTGCATTAATGGTTTGCCCCATGCATATAAGCATGTACATAATATTATATCC  120
            ||||||||||||||||||||||||||||||||||||||||||||||||||||||||||||
Sbjct  108  TGTACGTCGTGCATTAATGGTTTGCCCCATGCATATAAGCATGTACATAATATTATATCC  167

Query  121  TTACATAGGACATATTAACTCAATCTCATAATTCACTGATCTATCAACAGTAATCGAATG  180
            ||||||||||||||||||||||||||||||||||||||||||||||||||||||||||||
Sbjct  168  TTACATAGGACATATTAACTCAATCTCATAATTCACTGATCTATCAACAGTAATCGAATG  227

Query  181  CATATCACTTAGTCCAATAAGGGCTTAATCACCATGCCTCGAGAAAC  227
            |||||||||||||||||||||||||||||||||||||||||||||||
Sbjct  228  CATATCACTTAGTCCAATAAGGGCTTAATCACCATGCCTCGAGAAAC  274


>gb|HQ452429.1| Canis lupus familiaris haplotype A189 control region, partial
sequence; mitochondrial
```

```
Length=582

 Score = 420 bits (227),  Expect = 6e-114
 Identities = 227/227 (100%), Gaps = 0/227 (0%)
 Strand=Plus/Plus

Query  1    TTGAATCACCCCTACTGTGCTATGTCAGTATCTCCAGGTAAACCCTTCTCCCCTCCCCTA  60
            ||||||||||||||||||||||||||||||||||||||||||||||||||||||||||||
Sbjct  20   TTGAATCACCCCTACTGTGCTATGTCAGTATCTCCAGGTAAACCCTTCTCCCCTCCCCTA  79

Query  61   TGTACGTCGTGCATTAATGGTTTGCCCCATGCATATAAGCATGTACATAATATTATATCC  120
            ||||||||||||||||||||||||||||||||||||||||||||||||||||||||||||
Sbjct  80   TGTACGTCGTGCATTAATGGTTTGCCCCATGCATATAAGCATGTACATAATATTATATCC  139

Query  121  TTACATAGGACATATTAACTCAATCTCATAATTCACTGATCTATCAACAGTAATCGAATG  180
            ||||||||||||||||||||||||||||||||||||||||||||||||||||||||||||
Sbjct  140  TTACATAGGACATATTAACTCAATCTCATAATTCACTGATCTATCAACAGTAATCGAATG  199

Query  181  CATATCACTTAGTCCAATAAGGGCTTAATCACCATGCCTCGAGAAAC  227
            |||||||||||||||||||||||||||||||||||||||||||||||
Sbjct  200  CATATCACTTAGTCCAATAAGGGCTTAATCACCATGCCTCGAGAAAC  246


>gb|KC985190.1| Canis lupus familiaris D-loop, partial sequence; mitochondrial
Length=418

 Score = 420 bits (227),  Expect = 6e-114
 Identities = 227/227 (100%), Gaps = 0/227 (0%)
 Strand=Plus/Minus

Query  1    TTGAATCACCCCTACTGTGCTATGTCAGTATCTCCAGGTAAACCCTTCTCCCCTCCCCTA  60
            ||||||||||||||||||||||||||||||||||||||||||||||||||||||||||||
Sbjct  279  TTGAATCACCCCTACTGTGCTATGTCAGTATCTCCAGGTAAACCCTTCTCCCCTCCCCTA  220

Query  61   TGTACGTCGTGCATTAATGGTTTGCCCCATGCATATAAGCATGTACATAATATTATATCC  120
            ||||||||||||||||||||||||||||||||||||||||||||||||||||||||||||
Sbjct  219  TGTACGTCGTGCATTAATGGTTTGCCCCATGCATATAAGCATGTACATAATATTATATCC  160

Query  121  TTACATAGGACATATTAACTCAATCTCATAATTCACTGATCTATCAACAGTAATCGAATG  180
            ||||||||||||||||||||||||||||||||||||||||||||||||||||||||||||
Sbjct  159  TTACATAGGACATATTAACTCAATCTCATAATTCACTGATCTATCAACAGTAATCGAATG  100

Query  181  CATATCACTTAGTCCAATAAGGGCTTAATCACCATGCCTCGAGAAAC  227
            |||||||||||||||||||||||||||||||||||||||||||||||
Sbjct  99   CATATCACTTAGTCCAATAAGGGCTTAATCACCATGCCTCGAGAAAC  53


>gb|JX173682.1| Canis lupus familiaris isolate CAN1 D-loop, partial sequence;
mitochondrial
Length=413

 Score = 420 bits (227),  Expect = 6e-114
 Identities = 227/227 (100%), Gaps = 0/227 (0%)
```

```
 Strand=Plus/Plus

Query  1    TTGAATCACCCCTACTGTGCTATGTCAGTATCTCCAGGTAAACCCTTCTCCCCTCCCCTA  60
            ||||||||||||||||||||||||||||||||||||||||||||||||||||||||||||
Sbjct  76   TTGAATCACCCCTACTGTGCTATGTCAGTATCTCCAGGTAAACCCTTCTCCCCTCCCCTA  135

Query  61   TGTACGTCGTGCATTAATGGTTTGCCCCATGCATATAAGCATGTACATAATATTATATCC  120
            ||||||||||||||||||||||||||||||||||||||||||||||||||||||||||||
Sbjct  136  TGTACGTCGTGCATTAATGGTTTGCCCCATGCATATAAGCATGTACATAATATTATATCC  195

Query  121  TTACATAGGACATATTAACTCAATCTCATAATTCACTGATCTATCAACAGTAATCGAATG  180
            ||||||||||||||||||||||||||||||||||||||||||||||||||||||||||||
Sbjct  196  TTACATAGGACATATTAACTCAATCTCATAATTCACTGATCTATCAACAGTAATCGAATG  255

Query  181  CATATCACTTAGTCCAATAAGGGCTTAATCACCATGCCTCGAGAAAC  227
            |||||||||||||||||||||||||||||||||||||||||||||||
Sbjct  256  CATATCACTTAGTCCAATAAGGGCTTAATCACCATGCCTCGAGAAAC  302


>gb|JN182095.1| Canis lupus familiaris isolate greyhound3 cytochrome b gene,
partial cds; tRNA-Thr and tRNA-Pro genes, complete sequence;
and D-loop, partial sequence; mitochondrial
 gb|JN182129.1| Canis lupus x Canis lupus familiaris isolate phy1 cytochrome
b gene, partial cds; tRNA-Thr and tRNA-Pro genes, complete sequence;
and D-loop, partial sequence; mitochondrial
Length=1433

 Score = 420 bits (227),  Expect = 6e-114
 Identities = 227/227 (100%), Gaps = 0/227 (0%)
 Strand=Plus/Plus

Query  1    TTGAATCACCCCTACTGTGCTATGTCAGTATCTCCAGGTAAACCCTTCTCCCCTCCCCTA  60
            ||||||||||||||||||||||||||||||||||||||||||||||||||||||||||||
Sbjct  204  TTGAATCACCCCTACTGTGCTATGTCAGTATCTCCAGGTAAACCCTTCTCCCCTCCCCTA  263

Query  61   TGTACGTCGTGCATTAATGGTTTGCCCCATGCATATAAGCATGTACATAATATTATATCC  120
            ||||||||||||||||||||||||||||||||||||||||||||||||||||||||||||
Sbjct  264  TGTACGTCGTGCATTAATGGTTTGCCCCATGCATATAAGCATGTACATAATATTATATCC  323

Query  121  TTACATAGGACATATTAACTCAATCTCATAATTCACTGATCTATCAACAGTAATCGAATG  180
            ||||||||||||||||||||||||||||||||||||||||||||||||||||||||||||
Sbjct  324  TTACATAGGACATATTAACTCAATCTCATAATTCACTGATCTATCAACAGTAATCGAATG  383

Query  181  CATATCACTTAGTCCAATAAGGGCTTAATCACCATGCCTCGAGAAAC  227
            |||||||||||||||||||||||||||||||||||||||||||||||
Sbjct  384  CATATCACTTAGTCCAATAAGGGCTTAATCACCATGCCTCGAGAAAC  430


>gb|JN182116.1| Canis lupus familiaris isolate husky3 cytochrome b gene, partial
cds; tRNA-Thr and tRNA-Pro genes, complete sequence; and
D-loop, partial sequence; mitochondrial
Length=1373
```

```
 Score = 420 bits (227),  Expect = 6e-114
 Identities = 227/227 (100%), Gaps = 0/227 (0%)
 Strand=Plus/Plus

Query  1    TTGAATCACCCCTACTGTGCTATGTCAGTATCTCCAGGTAAACCCTTCTCCCCTCCCCTA  60
            ||||||||||||||||||||||||||||||||||||||||||||||||||||||||||||
Sbjct  204  TTGAATCACCCCTACTGTGCTATGTCAGTATCTCCAGGTAAACCCTTCTCCCCTCCCCTA  263

Query  61   TGTACGTCGTGCATTAATGGTTTGCCCCATGCATATAAGCATGTACATAATATTATATCC  120
            ||||||||||||||||||||||||||||||||||||||||||||||||||||||||||||
Sbjct  264  TGTACGTCGTGCATTAATGGTTTGCCCCATGCATATAAGCATGTACATAATATTATATCC  323

Query  121  TTACATAGGACATATTAACTCAATCTCATAATTCACTGATCTATCAACAGTAATCGAATG  180
            ||||||||||||||||||||||||||||||||||||||||||||||||||||||||||||
Sbjct  324  TTACATAGGACATATTAACTCAATCTCATAATTCACTGATCTATCAACAGTAATCGAATG  383

Query  181  CATATCACTTAGTCCAATAAGGGCTTAATCACCATGCCTCGAGAAAC  227
            |||||||||||||||||||||||||||||||||||||||||||||||
Sbjct  384  CATATCACTTAGTCCAATAAGGGCTTAATCACCATGCCTCGAGAAAC  430


>emb|HE687017.1| Canis lupus familiaris mitochondrial partial D-loop, breed: Bucovina
Shepherd Dog
Length=840

 Score = 420 bits (227),  Expect = 6e-114
 Identities = 227/227 (100%), Gaps = 0/227 (0%)
 Strand=Plus/Plus

Query  1    TTGAATCACCCCTACTGTGCTATGTCAGTATCTCCAGGTAAACCCTTCTCCCCTCCCCTA  60
            ||||||||||||||||||||||||||||||||||||||||||||||||||||||||||||
Sbjct  227  TTGAATCACCCCTACTGTGCTATGTCAGTATCTCCAGGTAAACCCTTCTCCCCTCCCCTA  286

Query  61   TGTACGTCGTGCATTAATGGTTTGCCCCATGCATATAAGCATGTACATAATATTATATCC  120
            ||||||||||||||||||||||||||||||||||||||||||||||||||||||||||||
Sbjct  287  TGTACGTCGTGCATTAATGGTTTGCCCCATGCATATAAGCATGTACATAATATTATATCC  346

Query  121  TTACATAGGACATATTAACTCAATCTCATAATTCACTGATCTATCAACAGTAATCGAATG  180
            ||||||||||||||||||||||||||||||||||||||||||||||||||||||||||||
Sbjct  347  TTACATAGGACATATTAACTCAATCTCATAATTCACTGATCTATCAACAGTAATCGAATG  406

Query  181  CATATCACTTAGTCCAATAAGGGCTTAATCACCATGCCTCGAGAAAC  227
            |||||||||||||||||||||||||||||||||||||||||||||||
Sbjct  407  CATATCACTTAGTCCAATAAGGGCTTAATCACCATGCCTCGAGAAAC  453


>gb|HQ452468.1| Canis lupus familiaris haplotype A171 control region, partial
sequence; mitochondrial
Length=582

 Score = 420 bits (227),  Expect = 6e-114
 Identities = 227/227 (100%), Gaps = 0/227 (0%)
 Strand=Plus/Plus
```

```
Query  1    TTGAATCACCCCTACTGTGCTATGTCAGTATCTCCAGGTAAACCCTTCTCCCCTCCCCTA  60
            ||||||||||||||||||||||||||||||||||||||||||||||||||||||||||||
Sbjct  20   TTGAATCACCCCTACTGTGCTATGTCAGTATCTCCAGGTAAACCCTTCTCCCCTCCCCTA  79

Query  61   TGTACGTCGTGCATTAATGGTTTGCCCCATGCATATAAGCATGTACATAATATTATATCC  120
            ||||||||||||||||||||||||||||||||||||||||||||||||||||||||||||
Sbjct  80   TGTACGTCGTGCATTAATGGTTTGCCCCATGCATATAAGCATGTACATAATATTATATCC  139

Query  121  TTACATAGGACATATTAACTCAATCTCATAATTCACTGATCTATCAACAGTAATCGAATG  180
            ||||||||||||||||||||||||||||||||||||||||||||||||||||||||||||
Sbjct  140  TTACATAGGACATATTAACTCAATCTCATAATTCACTGATCTATCAACAGTAATCGAATG  199

Query  181  CATATCACTTAGTCCAATAAGGGCTTAATCACCATGCCTCGAGAAAC  227
            |||||||||||||||||||||||||||||||||||||||||||||||
Sbjct  200  CATATCACTTAGTCCAATAAGGGCTTAATCACCATGCCTCGAGAAAC  246


>gb|HQ452422.1| Canis lupus familiaris haplotype A175 control region, partial
sequence; mitochondrial
Length=582

 Score = 420 bits (227),  Expect = 6e-114
 Identities = 227/227 (100%), Gaps = 0/227 (0%)
 Strand=Plus/Plus

Query  1    TTGAATCACCCCTACTGTGCTATGTCAGTATCTCCAGGTAAACCCTTCTCCCCTCCCCTA  60
            ||||||||||||||||||||||||||||||||||||||||||||||||||||||||||||
Sbjct  20   TTGAATCACCCCTACTGTGCTATGTCAGTATCTCCAGGTAAACCCTTCTCCCCTCCCCTA  79

Query  61   TGTACGTCGTGCATTAATGGTTTGCCCCATGCATATAAGCATGTACATAATATTATATCC  120
            ||||||||||||||||||||||||||||||||||||||||||||||||||||||||||||
Sbjct  80   TGTACGTCGTGCATTAATGGTTTGCCCCATGCATATAAGCATGTACATAATATTATATCC  139

Query  121  TTACATAGGACATATTAACTCAATCTCATAATTCACTGATCTATCAACAGTAATCGAATG  180
            ||||||||||||||||||||||||||||||||||||||||||||||||||||||||||||
Sbjct  140  TTACATAGGACATATTAACTCAATCTCATAATTCACTGATCTATCAACAGTAATCGAATG  199

Query  181  CATATCACTTAGTCCAATAAGGGCTTAATCACCATGCCTCGAGAAAC  227
            |||||||||||||||||||||||||||||||||||||||||||||||
Sbjct  200  CATATCACTTAGTCCAATAAGGGCTTAATCACCATGCCTCGAGAAAC  246


>gb|JF342891.1| Canis lupus familiaris haplotype HV1 A11 mitochondrion, partial
genome
Length=16736

 Score = 420 bits (227),  Expect = 6e-114
 Identities = 227/227 (100%), Gaps = 0/227 (0%)
 Strand=Plus/Plus

Query  1      TTGAATCACCCCTACTGTGCTATGTCAGTATCTCCAGGTAAACCCTTCTCCCCTCCCCTA  60
              ||||||||||||||||||||||||||||||||||||||||||||||||||||||||||||
```

```
Sbjct  15480  TTGAATCACCCCTACTGTGCTATGTCAGTATCTCCAGGTAAACCCTTCTCCCCTCCCCTA  15539

Query  61     TGTACGTCGTGCATTAATGGTTTGCCCCATGCATATAAGCATGTACATAATATTATATCC  120
              ||||||||||||||||||||||||||||||||||||||||||||||||||||||||||||
Sbjct  15540  TGTACGTCGTGCATTAATGGTTTGCCCCATGCATATAAGCATGTACATAATATTATATCC  15599

Query  121    TTACATAGGACATATTAACTCAATCTCATAATTCACTGATCTATCAACAGTAATCGAATG  180
              ||||||||||||||||||||||||||||||||||||||||||||||||||||||||||||
Sbjct  15600  TTACATAGGACATATTAACTCAATCTCATAATTCACTGATCTATCAACAGTAATCGAATG  15659

Query  181    CATATCACTTAGTCCAATAAGGGCTTAATCACCATGCCTCGAGAAAC  227
              |||||||||||||||||||||||||||||||||||||||||||||||
Sbjct  15660  CATATCACTTAGTCCAATAAGGGCTTAATCACCATGCCTCGAGAAAC  15706


>gb|JF342882.1| Canis lupus familiaris haplotype HV1 A11 mitochondrion, partial
genome
Length=16736

 Score = 420 bits (227),  Expect = 6e-114
 Identities = 227/227 (100%), Gaps = 0/227 (0%)
 Strand=Plus/Plus

Query  1      TTGAATCACCCCTACTGTGCTATGTCAGTATCTCCAGGTAAACCCTTCTCCCCTCCCCTA  60
              ||||||||||||||||||||||||||||||||||||||||||||||||||||||||||||
Sbjct  15480  TTGAATCACCCCTACTGTGCTATGTCAGTATCTCCAGGTAAACCCTTCTCCCCTCCCCTA  15539

Query  61     TGTACGTCGTGCATTAATGGTTTGCCCCATGCATATAAGCATGTACATAATATTATATCC  120
              ||||||||||||||||||||||||||||||||||||||||||||||||||||||||||||
Sbjct  15540  TGTACGTCGTGCATTAATGGTTTGCCCCATGCATATAAGCATGTACATAATATTATATCC  15599

Query  121    TTACATAGGACATATTAACTCAATCTCATAATTCACTGATCTATCAACAGTAATCGAATG  180
              ||||||||||||||||||||||||||||||||||||||||||||||||||||||||||||
Sbjct  15600  TTACATAGGACATATTAACTCAATCTCATAATTCACTGATCTATCAACAGTAATCGAATG  15659

Query  181    CATATCACTTAGTCCAATAAGGGCTTAATCACCATGCCTCGAGAAAC  227
              |||||||||||||||||||||||||||||||||||||||||||||||
Sbjct  15660  CATATCACTTAGTCCAATAAGGGCTTAATCACCATGCCTCGAGAAAC  15706


>gb|JF342858.1| Canis lupus familiaris haplotype HV1 A11 mitochondrion, partial
genome
Length=16736

 Score = 420 bits (227),  Expect = 6e-114
 Identities = 227/227 (100%), Gaps = 0/227 (0%)
 Strand=Plus/Plus

Query  1      TTGAATCACCCCTACTGTGCTATGTCAGTATCTCCAGGTAAACCCTTCTCCCCTCCCCTA  60
              ||||||||||||||||||||||||||||||||||||||||||||||||||||||||||||
Sbjct  15480  TTGAATCACCCCTACTGTGCTATGTCAGTATCTCCAGGTAAACCCTTCTCCCCTCCCCTA  15539

Query  61     TGTACGTCGTGCATTAATGGTTTGCCCCATGCATATAAGCATGTACATAATATTATATCC  120
```

```
             ||||||||||||||||||||||||||||||||||||||||||||||||||||||||||||
Sbjct  15540  TGTACGTCGTGCATTAATGGTTTGCCCCATGCATATAAGCATGTACATAATATTATATCC  15599

Query  121    TTACATAGGACATATTAACTCAATCTCATAATTCACTGATCTATCAACAGTAATCGAATG  180
              ||||||||||||||||||||||||||||||||||||||||||||||||||||||||||||
Sbjct  15600  TTACATAGGACATATTAACTCAATCTCATAATTCACTGATCTATCAACAGTAATCGAATG  15659

Query  181    CATATCACTTAGTCCAATAAGGGCTTAATCACCATGCCTCGAGAAAC  227
              |||||||||||||||||||||||||||||||||||||||||||||||
Sbjct  15660  CATATCACTTAGTCCAATAAGGGCTTAATCACCATGCCTCGAGAAAC  15706


>gb|JF342857.1| Canis lupus familiaris haplotype HV1 A11 mitochondrion, partial
genome
 gb|JF342865.1| Canis lupus familiaris haplotype HV1 A11 mitochondrion, partial
genome
 gb|JF342877.1| Canis lupus familiaris haplotype HV1 A11 mitochondrion, partial
genome
Length=16736

 Score = 420 bits (227),  Expect = 6e-114
 Identities = 227/227 (100%), Gaps = 0/227 (0%)
 Strand=Plus/Plus

Query  1      TTGAATCACCCCTACTGTGCTATGTCAGTATCTCCAGGTAAACCCTTCTCCCCTCCCCTA  60
              ||||||||||||||||||||||||||||||||||||||||||||||||||||||||||||
Sbjct  15480  TTGAATCACCCCTACTGTGCTATGTCAGTATCTCCAGGTAAACCCTTCTCCCCTCCCCTA  15539

Query  61     TGTACGTCGTGCATTAATGGTTTGCCCCATGCATATAAGCATGTACATAATATTATATCC  120
              ||||||||||||||||||||||||||||||||||||||||||||||||||||||||||||
Sbjct  15540  TGTACGTCGTGCATTAATGGTTTGCCCCATGCATATAAGCATGTACATAATATTATATCC  15599

Query  121    TTACATAGGACATATTAACTCAATCTCATAATTCACTGATCTATCAACAGTAATCGAATG  180
              ||||||||||||||||||||||||||||||||||||||||||||||||||||||||||||
Sbjct  15600  TTACATAGGACATATTAACTCAATCTCATAATTCACTGATCTATCAACAGTAATCGAATG  15659

Query  181    CATATCACTTAGTCCAATAAGGGCTTAATCACCATGCCTCGAGAAAC  227
              |||||||||||||||||||||||||||||||||||||||||||||||
Sbjct  15660  CATATCACTTAGTCCAATAAGGGCTTAATCACCATGCCTCGAGAAAC  15706


>gb|JF342835.1| Canis lupus familiaris haplotype HV1 A11 mitochondrion, partial
genome
Length=16735

 Score = 420 bits (227),  Expect = 6e-114
 Identities = 227/227 (100%), Gaps = 0/227 (0%)
 Strand=Plus/Plus

Query  1      TTGAATCACCCCTACTGTGCTATGTCAGTATCTCCAGGTAAACCCTTCTCCCCTCCCCTA  60
              ||||||||||||||||||||||||||||||||||||||||||||||||||||||||||||
Sbjct  15479  TTGAATCACCCCTACTGTGCTATGTCAGTATCTCCAGGTAAACCCTTCTCCCCTCCCCTA  15538
```

```
Query  61     TGTACGTCGTGCATTAATGGTTTGCCCCATGCATATAAGCATGTACATAATATTATATCC  120
              ||||||||||||||||||||||||||||||||||||||||||||||||||||||||||||
Sbjct  15539  TGTACGTCGTGCATTAATGGTTTGCCCCATGCATATAAGCATGTACATAATATTATATCC  15598

Query  121    TTACATAGGACATATTAACTCAATCTCATAATTCACTGATCTATCAACAGTAATCGAATG  180
              ||||||||||||||||||||||||||||||||||||||||||||||||||||||||||||
Sbjct  15599  TTACATAGGACATATTAACTCAATCTCATAATTCACTGATCTATCAACAGTAATCGAATG  15658

Query  181    CATATCACTTAGTCCAATAAGGGCTTAATCACCATGCCTCGAGAAAC  227
              |||||||||||||||||||||||||||||||||||||||||||||||
Sbjct  15659  CATATCACTTAGTCCAATAAGGGCTTAATCACCATGCCTCGAGAAAC  15705


>gb|JF342833.1| Canis lupus familiaris haplotype HV1 A11 mitochondrion, partial
genome
Length=16737

 Score = 420 bits (227),  Expect = 6e-114
 Identities = 227/227 (100%), Gaps = 0/227 (0%)
 Strand=Plus/Plus

Query  1      TTGAATCACCCCTACTGTGCTATGTCAGTATCTCCAGGTAAACCCTTCTCCCCTCCCCTA  60
              ||||||||||||||||||||||||||||||||||||||||||||||||||||||||||||
Sbjct  15480  TTGAATCACCCCTACTGTGCTATGTCAGTATCTCCAGGTAAACCCTTCTCCCCTCCCCTA  15539

Query  61     TGTACGTCGTGCATTAATGGTTTGCCCCATGCATATAAGCATGTACATAATATTATATCC  120
              ||||||||||||||||||||||||||||||||||||||||||||||||||||||||||||
Sbjct  15540  TGTACGTCGTGCATTAATGGTTTGCCCCATGCATATAAGCATGTACATAATATTATATCC  15599

Query  121    TTACATAGGACATATTAACTCAATCTCATAATTCACTGATCTATCAACAGTAATCGAATG  180
              ||||||||||||||||||||||||||||||||||||||||||||||||||||||||||||
Sbjct  15600  TTACATAGGACATATTAACTCAATCTCATAATTCACTGATCTATCAACAGTAATCGAATG  15659

Query  181    CATATCACTTAGTCCAATAAGGGCTTAATCACCATGCCTCGAGAAAC  227
              |||||||||||||||||||||||||||||||||||||||||||||||
Sbjct  15660  CATATCACTTAGTCCAATAAGGGCTTAATCACCATGCCTCGAGAAAC  15706


>gb|JF342827.1| Canis lupus familiaris haplotype HV1 A11 mitochondrion, partial
genome
Length=16736

 Score = 420 bits (227),  Expect = 6e-114
 Identities = 227/227 (100%), Gaps = 0/227 (0%)
 Strand=Plus/Plus

Query  1      TTGAATCACCCCTACTGTGCTATGTCAGTATCTCCAGGTAAACCCTTCTCCCCTCCCCTA  60
              ||||||||||||||||||||||||||||||||||||||||||||||||||||||||||||
Sbjct  15480  TTGAATCACCCCTACTGTGCTATGTCAGTATCTCCAGGTAAACCCTTCTCCCCTCCCCTA  15539

Query  61     TGTACGTCGTGCATTAATGGTTTGCCCCATGCATATAAGCATGTACATAATATTATATCC  120
              ||||||||||||||||||||||||||||||||||||||||||||||||||||||||||||
Sbjct  15540  TGTACGTCGTGCATTAATGGTTTGCCCCATGCATATAAGCATGTACATAATATTATATCC  15599
```

```
Query  121    TTACATAGGACATATTAACTCAATCTCATAATTCACTGATCTATCAACAGTAATCGAATG  180
              ||||||||||||||||||||||||||||||||||||||||||||||||||||||||||||
Sbjct  15600  TTACATAGGACATATTAACTCAATCTCATAATTCACTGATCTATCAACAGTAATCGAATG  15659

Query  181    CATATCACTTAGTCCAATAAGGGCTTAATCACCATGCCTCGAGAAAC  227
              |||||||||||||||||||||||||||||||||||||||||||||||
Sbjct  15660  CATATCACTTAGTCCAATAAGGGCTTAATCACCATGCCTCGAGAAAC  15706


>gb|JF342820.1| Canis lupus familiaris haplotype HV1 A11 mitochondrion, partial
genome
Length=16736

 Score = 420 bits (227),  Expect = 6e-114
 Identities = 227/227 (100%), Gaps = 0/227 (0%)
 Strand=Plus/Plus

Query  1      TTGAATCACCCCTACTGTGCTATGTCAGTATCTCCAGGTAAACCCTTCTCCCCTCCCCTA  60
              ||||||||||||||||||||||||||||||||||||||||||||||||||||||||||||
Sbjct  15480  TTGAATCACCCCTACTGTGCTATGTCAGTATCTCCAGGTAAACCCTTCTCCCCTCCCCTA  15539

Query  61     TGTACGTCGTGCATTAATGGTTTGCCCCATGCATATAAGCATGTACATAATATTATATCC  120
              ||||||||||||||||||||||||||||||||||||||||||||||||||||||||||||
Sbjct  15540  TGTACGTCGTGCATTAATGGTTTGCCCCATGCATATAAGCATGTACATAATATTATATCC  15599

Query  121    TTACATAGGACATATTAACTCAATCTCATAATTCACTGATCTATCAACAGTAATCGAATG  180
              ||||||||||||||||||||||||||||||||||||||||||||||||||||||||||||
Sbjct  15600  TTACATAGGACATATTAACTCAATCTCATAATTCACTGATCTATCAACAGTAATCGAATG  15659

Query  181    CATATCACTTAGTCCAATAAGGGCTTAATCACCATGCCTCGAGAAAC  227
              |||||||||||||||||||||||||||||||||||||||||||||||
Sbjct  15660  CATATCACTTAGTCCAATAAGGGCTTAATCACCATGCCTCGAGAAAC  15706


>gb|JF342814.1| Canis lupus familiaris haplotype HV1 A169* mitochondrion, partial
genome
Length=16736

 Score = 420 bits (227),  Expect = 6e-114
 Identities = 227/227 (100%), Gaps = 0/227 (0%)
 Strand=Plus/Plus

Query  1      TTGAATCACCCCTACTGTGCTATGTCAGTATCTCCAGGTAAACCCTTCTCCCCTCCCCTA  60
              ||||||||||||||||||||||||||||||||||||||||||||||||||||||||||||
Sbjct  15480  TTGAATCACCCCTACTGTGCTATGTCAGTATCTCCAGGTAAACCCTTCTCCCCTCCCCTA  15539

Query  61     TGTACGTCGTGCATTAATGGTTTGCCCCATGCATATAAGCATGTACATAATATTATATCC  120
              ||||||||||||||||||||||||||||||||||||||||||||||||||||||||||||
Sbjct  15540  TGTACGTCGTGCATTAATGGTTTGCCCCATGCATATAAGCATGTACATAATATTATATCC  15599

Query  121    TTACATAGGACATATTAACTCAATCTCATAATTCACTGATCTATCAACAGTAATCGAATG  180
              ||||||||||||||||||||||||||||||||||||||||||||||||||||||||||||
```

```
Sbjct  15600  TTACATAGGACATATTAACTCAATCTCATAATTCACTGATCTATCAACAGTAATCGAATG  15659

Query  181    CATATCACTTAGTCCAATAAGGGCTTAATCACCATGCCTCGAGAAAC  227
              |||||||||||||||||||||||||||||||||||||||||||||||
Sbjct  15660  CATATCACTTAGTCCAATAAGGGCTTAATCACCATGCCTCGAGAAAC  15706


>gb|JF342808.1| Canis lupus familiaris haplotype HV1 A11 mitochondrion, partial
genome
Length=16736

 Score = 420 bits (227),  Expect = 6e-114
 Identities = 227/227 (100%), Gaps = 0/227 (0%)
 Strand=Plus/Plus

Query  1      TTGAATCACCCCTACTGTGCTATGTCAGTATCTCCAGGTAAACCCTTCTCCCCTCCCCTA  60
              ||||||||||||||||||||||||||||||||||||||||||||||||||||||||||||
Sbjct  15480  TTGAATCACCCCTACTGTGCTATGTCAGTATCTCCAGGTAAACCCTTCTCCCCTCCCCTA  15539

Query  61     TGTACGTCGTGCATTAATGGTTTGCCCCATGCATATAAGCATGTACATAATATTATATCC  120
              ||||||||||||||||||||||||||||||||||||||||||||||||||||||||||||
Sbjct  15540  TGTACGTCGTGCATTAATGGTTTGCCCCATGCATATAAGCATGTACATAATATTATATCC  15599

Query  121    TTACATAGGACATATTAACTCAATCTCATAATTCACTGATCTATCAACAGTAATCGAATG  180
              ||||||||||||||||||||||||||||||||||||||||||||||||||||||||||||
Sbjct  15600  TTACATAGGACATATTAACTCAATCTCATAATTCACTGATCTATCAACAGTAATCGAATG  15659

Query  181    CATATCACTTAGTCCAATAAGGGCTTAATCACCATGCCTCGAGAAAC  227
              |||||||||||||||||||||||||||||||||||||||||||||||
Sbjct  15660  CATATCACTTAGTCCAATAAGGGCTTAATCACCATGCCTCGAGAAAC  15706


>dbj|AB605594.1| Canis lupus familiaris mitochondrial gene for tRNA-Pro, D-loop,
complete and partial sequence, isolate: Cf106
Length=710

 Score = 420 bits (227),  Expect = 6e-114
 Identities = 227/227 (100%), Gaps = 0/227 (0%)
 Strand=Plus/Plus

Query  1    TTGAATCACCCCTACTGTGCTATGTCAGTATCTCCAGGTAAACCCTTCTCCCCTCCCCTA  60
            ||||||||||||||||||||||||||||||||||||||||||||||||||||||||||||
Sbjct  86   TTGAATCACCCCTACTGTGCTATGTCAGTATCTCCAGGTAAACCCTTCTCCCCTCCCCTA  145

Query  61   TGTACGTCGTGCATTAATGGTTTGCCCCATGCATATAAGCATGTACATAATATTATATCC  120
            ||||||||||||||||||||||||||||||||||||||||||||||||||||||||||||
Sbjct  146  TGTACGTCGTGCATTAATGGTTTGCCCCATGCATATAAGCATGTACATAATATTATATCC  205

Query  121  TTACATAGGACATATTAACTCAATCTCATAATTCACTGATCTATCAACAGTAATCGAATG  180
            ||||||||||||||||||||||||||||||||||||||||||||||||||||||||||||
Sbjct  206  TTACATAGGACATATTAACTCAATCTCATAATTCACTGATCTATCAACAGTAATCGAATG  265

Query  181  CATATCACTTAGTCCAATAAGGGCTTAATCACCATGCCTCGAGAAAC  227
```

```
            |||||||||||||||||||||||||||||||||||||||||||||||
Sbjct  266  CATATCACTTAGTCCAATAAGGGCTTAATCACCATGCCTCGAGAAAC  312


>dbj|AB605582.1| Canis lupus familiaris mitochondrial gene for tRNA-Pro, D-loop,
complete and partial sequence, isolate: Cf94
Length=710

 Score = 420 bits (227),  Expect = 6e-114
 Identities = 227/227 (100%), Gaps = 0/227 (0%)
 Strand=Plus/Plus

Query  1    TTGAATCACCCCTACTGTGCTATGTCAGTATCTCCAGGTAAACCCTTCTCCCCTCCCCTA  60
            ||||||||||||||||||||||||||||||||||||||||||||||||||||||||||||
Sbjct  86   TTGAATCACCCCTACTGTGCTATGTCAGTATCTCCAGGTAAACCCTTCTCCCCTCCCCTA  145

Query  61   TGTACGTCGTGCATTAATGGTTTGCCCCATGCATATAAGCATGTACATAATATTATATCC  120
            ||||||||||||||||||||||||||||||||||||||||||||||||||||||||||||
Sbjct  146  TGTACGTCGTGCATTAATGGTTTGCCCCATGCATATAAGCATGTACATAATATTATATCC  205

Query  121  TTACATAGGACATATTAACTCAATCTCATAATTCACTGATCTATCAACAGTAATCGAATG  180
            ||||||||||||||||||||||||||||||||||||||||||||||||||||||||||||
Sbjct  206  TTACATAGGACATATTAACTCAATCTCATAATTCACTGATCTATCAACAGTAATCGAATG  265

Query  181  CATATCACTTAGTCCAATAAGGGCTTAATCACCATGCCTCGAGAAAC  227
            |||||||||||||||||||||||||||||||||||||||||||||||
Sbjct  266  CATATCACTTAGTCCAATAAGGGCTTAATCACCATGCCTCGAGAAAC  312


>dbj|AB605581.1| Canis lupus familiaris mitochondrial gene for tRNA-Pro, D-loop,
complete and partial sequence, isolate: Cf93
Length=710

 Score = 420 bits (227),  Expect = 6e-114
 Identities = 227/227 (100%), Gaps = 0/227 (0%)
 Strand=Plus/Plus

Query  1    TTGAATCACCCCTACTGTGCTATGTCAGTATCTCCAGGTAAACCCTTCTCCCCTCCCCTA  60
            ||||||||||||||||||||||||||||||||||||||||||||||||||||||||||||
Sbjct  86   TTGAATCACCCCTACTGTGCTATGTCAGTATCTCCAGGTAAACCCTTCTCCCCTCCCCTA  145

Query  61   TGTACGTCGTGCATTAATGGTTTGCCCCATGCATATAAGCATGTACATAATATTATATCC  120
            ||||||||||||||||||||||||||||||||||||||||||||||||||||||||||||
Sbjct  146  TGTACGTCGTGCATTAATGGTTTGCCCCATGCATATAAGCATGTACATAATATTATATCC  205

Query  121  TTACATAGGACATATTAACTCAATCTCATAATTCACTGATCTATCAACAGTAATCGAATG  180
            ||||||||||||||||||||||||||||||||||||||||||||||||||||||||||||
Sbjct  206  TTACATAGGACATATTAACTCAATCTCATAATTCACTGATCTATCAACAGTAATCGAATG  265

Query  181  CATATCACTTAGTCCAATAAGGGCTTAATCACCATGCCTCGAGAAAC  227
            |||||||||||||||||||||||||||||||||||||||||||||||
Sbjct  266  CATATCACTTAGTCCAATAAGGGCTTAATCACCATGCCTCGAGAAAC  312
```

```
>dbj|AB605575.1| Canis lupus familiaris mitochondrial gene for tRNA-Pro, D-loop,
complete and partial sequence, isolate: Cf87
Length=710

 Score = 420 bits (227),  Expect = 6e-114
 Identities = 227/227 (100%), Gaps = 0/227 (0%)
 Strand=Plus/Plus

Query  1    TTGAATCACCCCTACTGTGCTATGTCAGTATCTCCAGGTAAACCCTTCTCCCCTCCCCTA  60
            ||||||||||||||||||||||||||||||||||||||||||||||||||||||||||||
Sbjct  86   TTGAATCACCCCTACTGTGCTATGTCAGTATCTCCAGGTAAACCCTTCTCCCCTCCCCTA  145

Query  61   TGTACGTCGTGCATTAATGGTTTGCCCCATGCATATAAGCATGTACATAATATTATATCC  120
            ||||||||||||||||||||||||||||||||||||||||||||||||||||||||||||
Sbjct  146  TGTACGTCGTGCATTAATGGTTTGCCCCATGCATATAAGCATGTACATAATATTATATCC  205

Query  121  TTACATAGGACATATTAACTCAATCTCATAATTCACTGATCTATCAACAGTAATCGAATG  180
            ||||||||||||||||||||||||||||||||||||||||||||||||||||||||||||
Sbjct  206  TTACATAGGACATATTAACTCAATCTCATAATTCACTGATCTATCAACAGTAATCGAATG  265

Query  181  CATATCACTTAGTCCAATAAGGGCTTAATCACCATGCCTCGAGAAAC  227
            |||||||||||||||||||||||||||||||||||||||||||||||
Sbjct  266  CATATCACTTAGTCCAATAAGGGCTTAATCACCATGCCTCGAGAAAC  312


>dbj|AB605570.1| Canis lupus familiaris mitochondrial gene for tRNA-Pro, D-loop,
complete and partial sequence, isolate: Cf82
Length=710

 Score = 420 bits (227),  Expect = 6e-114
 Identities = 227/227 (100%), Gaps = 0/227 (0%)
 Strand=Plus/Plus

Query  1    TTGAATCACCCCTACTGTGCTATGTCAGTATCTCCAGGTAAACCCTTCTCCCCTCCCCTA  60
            ||||||||||||||||||||||||||||||||||||||||||||||||||||||||||||
Sbjct  86   TTGAATCACCCCTACTGTGCTATGTCAGTATCTCCAGGTAAACCCTTCTCCCCTCCCCTA  145

Query  61   TGTACGTCGTGCATTAATGGTTTGCCCCATGCATATAAGCATGTACATAATATTATATCC  120
            ||||||||||||||||||||||||||||||||||||||||||||||||||||||||||||
Sbjct  146  TGTACGTCGTGCATTAATGGTTTGCCCCATGCATATAAGCATGTACATAATATTATATCC  205

Query  121  TTACATAGGACATATTAACTCAATCTCATAATTCACTGATCTATCAACAGTAATCGAATG  180
            ||||||||||||||||||||||||||||||||||||||||||||||||||||||||||||
Sbjct  206  TTACATAGGACATATTAACTCAATCTCATAATTCACTGATCTATCAACAGTAATCGAATG  265

Query  181  CATATCACTTAGTCCAATAAGGGCTTAATCACCATGCCTCGAGAAAC  227
            |||||||||||||||||||||||||||||||||||||||||||||||
Sbjct  266  CATATCACTTAGTCCAATAAGGGCTTAATCACCATGCCTCGAGAAAC  312


>dbj|AB605568.1| Canis lupus familiaris mitochondrial gene for tRNA-Pro, D-loop,
complete and partial sequence, isolate: Cf80
```

```
Length=710

 Score = 420 bits (227),  Expect = 6e-114
 Identities = 227/227 (100%), Gaps = 0/227 (0%)
 Strand=Plus/Plus

Query  1    TTGAATCACCCCTACTGTGCTATGTCAGTATCTCCAGGTAAACCCTTCTCCCCTCCCCTA  60
            ||||||||||||||||||||||||||||||||||||||||||||||||||||||||||||
Sbjct  86   TTGAATCACCCCTACTGTGCTATGTCAGTATCTCCAGGTAAACCCTTCTCCCCTCCCCTA  145

Query  61   TGTACGTCGTGCATTAATGGTTTGCCCCATGCATATAAGCATGTACATAATATTATATCC  120
            ||||||||||||||||||||||||||||||||||||||||||||||||||||||||||||
Sbjct  146  TGTACGTCGTGCATTAATGGTTTGCCCCATGCATATAAGCATGTACATAATATTATATCC  205

Query  121  TTACATAGGACATATTAACTCAATCTCATAATTCACTGATCTATCAACAGTAATCGAATG  180
            ||||||||||||||||||||||||||||||||||||||||||||||||||||||||||||
Sbjct  206  TTACATAGGACATATTAACTCAATCTCATAATTCACTGATCTATCAACAGTAATCGAATG  265

Query  181  CATATCACTTAGTCCAATAAGGGCTTAATCACCATGCCTCGAGAAAC  227
            |||||||||||||||||||||||||||||||||||||||||||||||
Sbjct  266  CATATCACTTAGTCCAATAAGGGCTTAATCACCATGCCTCGAGAAAC  312


>dbj|AB605558.1| Canis lupus familiaris mitochondrial gene for tRNA-Pro, D-loop,
complete and partial sequence, isolate: Cf70
Length=710

 Score = 420 bits (227),  Expect = 6e-114
 Identities = 227/227 (100%), Gaps = 0/227 (0%)
 Strand=Plus/Plus

Query  1    TTGAATCACCCCTACTGTGCTATGTCAGTATCTCCAGGTAAACCCTTCTCCCCTCCCCTA  60
            ||||||||||||||||||||||||||||||||||||||||||||||||||||||||||||
Sbjct  86   TTGAATCACCCCTACTGTGCTATGTCAGTATCTCCAGGTAAACCCTTCTCCCCTCCCCTA  145

Query  61   TGTACGTCGTGCATTAATGGTTTGCCCCATGCATATAAGCATGTACATAATATTATATCC  120
            ||||||||||||||||||||||||||||||||||||||||||||||||||||||||||||
Sbjct  146  TGTACGTCGTGCATTAATGGTTTGCCCCATGCATATAAGCATGTACATAATATTATATCC  205

Query  121  TTACATAGGACATATTAACTCAATCTCATAATTCACTGATCTATCAACAGTAATCGAATG  180
            ||||||||||||||||||||||||||||||||||||||||||||||||||||||||||||
Sbjct  206  TTACATAGGACATATTAACTCAATCTCATAATTCACTGATCTATCAACAGTAATCGAATG  265

Query  181  CATATCACTTAGTCCAATAAGGGCTTAATCACCATGCCTCGAGAAAC  227
            |||||||||||||||||||||||||||||||||||||||||||||||
Sbjct  266  CATATCACTTAGTCCAATAAGGGCTTAATCACCATGCCTCGAGAAAC  312


>dbj|AB605516.1| Canis lupus familiaris mitochondrial gene for tRNA-Pro, D-loop,
complete and partial sequence, isolate: Cf28
Length=710

 Score = 420 bits (227),  Expect = 6e-114
```

```
 Identities = 227/227 (100%), Gaps = 0/227 (0%)
 Strand=Plus/Plus

Query  1    TTGAATCACCCCTACTGTGCTATGTCAGTATCTCCAGGTAAACCCTTCTCCCCTCCCCTA  60
            ||||||||||||||||||||||||||||||||||||||||||||||||||||||||||||
Sbjct  86   TTGAATCACCCCTACTGTGCTATGTCAGTATCTCCAGGTAAACCCTTCTCCCCTCCCCTA  145

Query  61   TGTACGTCGTGCATTAATGGTTTGCCCCATGCATATAAGCATGTACATAATATTATATCC  120
            ||||||||||||||||||||||||||||||||||||||||||||||||||||||||||||
Sbjct  146  TGTACGTCGTGCATTAATGGTTTGCCCCATGCATATAAGCATGTACATAATATTATATCC  205

Query  121  TTACATAGGACATATTAACTCAATCTCATAATTCACTGATCTATCAACAGTAATCGAATG  180
            ||||||||||||||||||||||||||||||||||||||||||||||||||||||||||||
Sbjct  206  TTACATAGGACATATTAACTCAATCTCATAATTCACTGATCTATCAACAGTAATCGAATG  265

Query  181  CATATCACTTAGTCCAATAAGGGCTTAATCACCATGCCTCGAGAAAC  227
            |||||||||||||||||||||||||||||||||||||||||||||||
Sbjct  266  CATATCACTTAGTCCAATAAGGGCTTAATCACCATGCCTCGAGAAAC  312


>dbj|AB605492.1| Canis lupus familiaris mitochondrial gene for tRNA-Pro, D-loop,
complete and partial sequence, isolate: Cf04
Length=710

 Score = 420 bits (227),  Expect = 6e-114
 Identities = 227/227 (100%), Gaps = 0/227 (0%)
 Strand=Plus/Plus

Query  1    TTGAATCACCCCTACTGTGCTATGTCAGTATCTCCAGGTAAACCCTTCTCCCCTCCCCTA  60
            ||||||||||||||||||||||||||||||||||||||||||||||||||||||||||||
Sbjct  86   TTGAATCACCCCTACTGTGCTATGTCAGTATCTCCAGGTAAACCCTTCTCCCCTCCCCTA  145

Query  61   TGTACGTCGTGCATTAATGGTTTGCCCCATGCATATAAGCATGTACATAATATTATATCC  120
            ||||||||||||||||||||||||||||||||||||||||||||||||||||||||||||
Sbjct  146  TGTACGTCGTGCATTAATGGTTTGCCCCATGCATATAAGCATGTACATAATATTATATCC  205

Query  121  TTACATAGGACATATTAACTCAATCTCATAATTCACTGATCTATCAACAGTAATCGAATG  180
            ||||||||||||||||||||||||||||||||||||||||||||||||||||||||||||
Sbjct  206  TTACATAGGACATATTAACTCAATCTCATAATTCACTGATCTATCAACAGTAATCGAATG  265

Query  181  CATATCACTTAGTCCAATAAGGGCTTAATCACCATGCCTCGAGAAAC  227
            |||||||||||||||||||||||||||||||||||||||||||||||
Sbjct  266  CATATCACTTAGTCCAATAAGGGCTTAATCACCATGCCTCGAGAAAC  312


>gb|HQ452457.1| Canis lupus familiaris haplotype A226 control region, partial
sequence; mitochondrial
 gb|KJ139135.1| Canis lupus familiaris isolate 1810_MIN3 D-loop, partial sequence;
mitochondrial
 gb|KJ139136.1| Canis lupus familiaris isolate 1707_CAL3 D-loop, partial sequence;
mitochondrial
Length=582
```

```
 Score = 420 bits (227),  Expect = 6e-114
 Identities = 227/227 (100%), Gaps = 0/227 (0%)
 Strand=Plus/Plus

Query  1    TTGAATCACCCCTACTGTGCTATGTCAGTATCTCCAGGTAAACCCTTCTCCCCTCCCCTA  60
            ||||||||||||||||||||||||||||||||||||||||||||||||||||||||||||
Sbjct  20   TTGAATCACCCCTACTGTGCTATGTCAGTATCTCCAGGTAAACCCTTCTCCCCTCCCCTA  79

Query  61   TGTACGTCGTGCATTAATGGTTTGCCCCATGCATATAAGCATGTACATAATATTATATCC  120
            ||||||||||||||||||||||||||||||||||||||||||||||||||||||||||||
Sbjct  80   TGTACGTCGTGCATTAATGGTTTGCCCCATGCATATAAGCATGTACATAATATTATATCC  139

Query  121  TTACATAGGACATATTAACTCAATCTCATAATTCACTGATCTATCAACAGTAATCGAATG  180
            ||||||||||||||||||||||||||||||||||||||||||||||||||||||||||||
Sbjct  140  TTACATAGGACATATTAACTCAATCTCATAATTCACTGATCTATCAACAGTAATCGAATG  199

Query  181  CATATCACTTAGTCCAATAAGGGCTTAATCACCATGCCTCGAGAAAC  227
            |||||||||||||||||||||||||||||||||||||||||||||||
Sbjct  200  CATATCACTTAGTCCAATAAGGGCTTAATCACCATGCCTCGAGAAAC  246


>dbj|AB622564.1| Canis lupus familiaris DNA, hypervariable region 1 (control region),
partial sequence, haplotype: NVLU052
Length=660

 Score = 420 bits (227),  Expect = 6e-114
 Identities = 227/227 (100%), Gaps = 0/227 (0%)
 Strand=Plus/Plus

Query  1    TTGAATCACCCCTACTGTGCTATGTCAGTATCTCCAGGTAAACCCTTCTCCCCTCCCCTA  60
            ||||||||||||||||||||||||||||||||||||||||||||||||||||||||||||
Sbjct  25   TTGAATCACCCCTACTGTGCTATGTCAGTATCTCCAGGTAAACCCTTCTCCCCTCCCCTA  84

Query  61   TGTACGTCGTGCATTAATGGTTTGCCCCATGCATATAAGCATGTACATAATATTATATCC  120
            ||||||||||||||||||||||||||||||||||||||||||||||||||||||||||||
Sbjct  85   TGTACGTCGTGCATTAATGGTTTGCCCCATGCATATAAGCATGTACATAATATTATATCC  144

Query  121  TTACATAGGACATATTAACTCAATCTCATAATTCACTGATCTATCAACAGTAATCGAATG  180
            ||||||||||||||||||||||||||||||||||||||||||||||||||||||||||||
Sbjct  145  TTACATAGGACATATTAACTCAATCTCATAATTCACTGATCTATCAACAGTAATCGAATG  204

Query  181  CATATCACTTAGTCCAATAAGGGCTTAATCACCATGCCTCGAGAAAC  227
            |||||||||||||||||||||||||||||||||||||||||||||||
Sbjct  205  CATATCACTTAGTCCAATAAGGGCTTAATCACCATGCCTCGAGAAAC  251


>dbj|AB622563.1| Canis lupus familiaris DNA, hypervariable region 1 (control region),
partial sequence, haplotype: NVLU051
Length=660

 Score = 420 bits (227),  Expect = 6e-114
 Identities = 227/227 (100%), Gaps = 0/227 (0%)
 Strand=Plus/Plus
```

```
Query  1    TTGAATCACCCCTACTGTGCTATGTCAGTATCTCCAGGTAAACCCTTCTCCCCTCCCCTA  60
            ||||||||||||||||||||||||||||||||||||||||||||||||||||||||||||
Sbjct  25   TTGAATCACCCCTACTGTGCTATGTCAGTATCTCCAGGTAAACCCTTCTCCCCTCCCCTA  84

Query  61   TGTACGTCGTGCATTAATGGTTTGCCCCATGCATATAAGCATGTACATAATATTATATCC  120
            ||||||||||||||||||||||||||||||||||||||||||||||||||||||||||||
Sbjct  85   TGTACGTCGTGCATTAATGGTTTGCCCCATGCATATAAGCATGTACATAATATTATATCC  144

Query  121  TTACATAGGACATATTAACTCAATCTCATAATTCACTGATCTATCAACAGTAATCGAATG  180
            ||||||||||||||||||||||||||||||||||||||||||||||||||||||||||||
Sbjct  145  TTACATAGGACATATTAACTCAATCTCATAATTCACTGATCTATCAACAGTAATCGAATG  204

Query  181  CATATCACTTAGTCCAATAAGGGCTTAATCACCATGCCTCGAGAAAC  227
            |||||||||||||||||||||||||||||||||||||||||||||||
Sbjct  205  CATATCACTTAGTCCAATAAGGGCTTAATCACCATGCCTCGAGAAAC  251


>dbj|AB622562.1| Canis lupus familiaris DNA, hypervariable region 1 (control region),
partial sequence, haplotype: NVLU050
Length=660

 Score = 420 bits (227),  Expect = 6e-114
 Identities = 227/227 (100%), Gaps = 0/227 (0%)
 Strand=Plus/Plus

Query  1    TTGAATCACCCCTACTGTGCTATGTCAGTATCTCCAGGTAAACCCTTCTCCCCTCCCCTA  60
            ||||||||||||||||||||||||||||||||||||||||||||||||||||||||||||
Sbjct  25   TTGAATCACCCCTACTGTGCTATGTCAGTATCTCCAGGTAAACCCTTCTCCCCTCCCCTA  84

Query  61   TGTACGTCGTGCATTAATGGTTTGCCCCATGCATATAAGCATGTACATAATATTATATCC  120
            ||||||||||||||||||||||||||||||||||||||||||||||||||||||||||||
Sbjct  85   TGTACGTCGTGCATTAATGGTTTGCCCCATGCATATAAGCATGTACATAATATTATATCC  144

Query  121  TTACATAGGACATATTAACTCAATCTCATAATTCACTGATCTATCAACAGTAATCGAATG  180
            ||||||||||||||||||||||||||||||||||||||||||||||||||||||||||||
Sbjct  145  TTACATAGGACATATTAACTCAATCTCATAATTCACTGATCTATCAACAGTAATCGAATG  204

Query  181  CATATCACTTAGTCCAATAAGGGCTTAATCACCATGCCTCGAGAAAC  227
            |||||||||||||||||||||||||||||||||||||||||||||||
Sbjct  205  CATATCACTTAGTCCAATAAGGGCTTAATCACCATGCCTCGAGAAAC  251


>dbj|AB622561.1| Canis lupus familiaris DNA, hypervariable region 1 (control region),
partial sequence, haplotype: NVLU049
Length=660

 Score = 420 bits (227),  Expect = 6e-114
 Identities = 227/227 (100%), Gaps = 0/227 (0%)
 Strand=Plus/Plus

Query  1    TTGAATCACCCCTACTGTGCTATGTCAGTATCTCCAGGTAAACCCTTCTCCCCTCCCCTA  60
            ||||||||||||||||||||||||||||||||||||||||||||||||||||||||||||
```

```
Sbjct  25   TTGAATCACCCCTACTGTGCTATGTCAGTATCTCCAGGTAAACCCTTCTCCCCTCCCCTA  84

Query  61   TGTACGTCGTGCATTAATGGTTTGCCCCATGCATATAAGCATGTACATAATATTATATCC  120
            ||||||||||||||||||||||||||||||||||||||||||||||||||||||||||||
Sbjct  85   TGTACGTCGTGCATTAATGGTTTGCCCCATGCATATAAGCATGTACATAATATTATATCC  144

Query  121  TTACATAGGACATATTAACTCAATCTCATAATTCACTGATCTATCAACAGTAATCGAATG  180
            ||||||||||||||||||||||||||||||||||||||||||||||||||||||||||||
Sbjct  145  TTACATAGGACATATTAACTCAATCTCATAATTCACTGATCTATCAACAGTAATCGAATG  204

Query  181  CATATCACTTAGTCCAATAAGGGCTTAATCACCATGCCTCGAGAAAC  227
            |||||||||||||||||||||||||||||||||||||||||||||||
Sbjct  205  CATATCACTTAGTCCAATAAGGGCTTAATCACCATGCCTCGAGAAAC  251


>gb|HQ997560.1| Canis lupus familiaris isolate 231 D-loop, partial sequence;
mitochondrial
Length=673

 Score = 420 bits (227),  Expect = 6e-114
 Identities = 227/227 (100%), Gaps = 0/227 (0%)
 Strand=Plus/Plus

Query  1    TTGAATCACCCCTACTGTGCTATGTCAGTATCTCCAGGTAAACCCTTCTCCCCTCCCCTA  60
            ||||||||||||||||||||||||||||||||||||||||||||||||||||||||||||
Sbjct  20   TTGAATCACCCCTACTGTGCTATGTCAGTATCTCCAGGTAAACCCTTCTCCCCTCCCCTA  79

Query  61   TGTACGTCGTGCATTAATGGTTTGCCCCATGCATATAAGCATGTACATAATATTATATCC  120
            ||||||||||||||||||||||||||||||||||||||||||||||||||||||||||||
Sbjct  80   TGTACGTCGTGCATTAATGGTTTGCCCCATGCATATAAGCATGTACATAATATTATATCC  139

Query  121  TTACATAGGACATATTAACTCAATCTCATAATTCACTGATCTATCAACAGTAATCGAATG  180
            ||||||||||||||||||||||||||||||||||||||||||||||||||||||||||||
Sbjct  140  TTACATAGGACATATTAACTCAATCTCATAATTCACTGATCTATCAACAGTAATCGAATG  199

Query  181  CATATCACTTAGTCCAATAAGGGCTTAATCACCATGCCTCGAGAAAC  227
            |||||||||||||||||||||||||||||||||||||||||||||||
Sbjct  200  CATATCACTTAGTCCAATAAGGGCTTAATCACCATGCCTCGAGAAAC  246


>gb|HQ997531.1| Canis lupus familiaris isolate 201 D-loop, partial sequence;
mitochondrial
Length=673

 Score = 420 bits (227),  Expect = 6e-114
 Identities = 227/227 (100%), Gaps = 0/227 (0%)
 Strand=Plus/Plus

Query  1    TTGAATCACCCCTACTGTGCTATGTCAGTATCTCCAGGTAAACCCTTCTCCCCTCCCCTA  60
            ||||||||||||||||||||||||||||||||||||||||||||||||||||||||||||
Sbjct  20   TTGAATCACCCCTACTGTGCTATGTCAGTATCTCCAGGTAAACCCTTCTCCCCTCCCCTA  79

Query  61   TGTACGTCGTGCATTAATGGTTTGCCCCATGCATATAAGCATGTACATAATATTATATCC  120
```

```
             ||||||||||||||||||||||||||||||||||||||||||||||||||||||||||||
Sbjct  80    TGTACGTCGTGCATTAATGGTTTGCCCCATGCATATAAGCATGTACATAATATTATATCC  139

Query  121   TTACATAGGACATATTAACTCAATCTCATAATTCACTGATCTATCAACAGTAATCGAATG  180
             ||||||||||||||||||||||||||||||||||||||||||||||||||||||||||||
Sbjct  140   TTACATAGGACATATTAACTCAATCTCATAATTCACTGATCTATCAACAGTAATCGAATG  199

Query  181   CATATCACTTAGTCCAATAAGGGCTTAATCACCATGCCTCGAGAAAC  227
             |||||||||||||||||||||||||||||||||||||||||||||||
Sbjct  200   CATATCACTTAGTCCAATAAGGGCTTAATCACCATGCCTCGAGAAAC  246


>gb|HQ997493.1| Canis lupus familiaris isolate 121 D-loop, partial sequence;
mitochondrial
Length=673

 Score = 420 bits (227),  Expect = 6e-114
 Identities = 227/227 (100%), Gaps = 0/227 (0%)
 Strand=Plus/Plus

Query  1     TTGAATCACCCCTACTGTGCTATGTCAGTATCTCCAGGTAAACCCTTCTCCCCTCCCCTA  60
             ||||||||||||||||||||||||||||||||||||||||||||||||||||||||||||
Sbjct  20    TTGAATCACCCCTACTGTGCTATGTCAGTATCTCCAGGTAAACCCTTCTCCCCTCCCCTA  79

Query  61    TGTACGTCGTGCATTAATGGTTTGCCCCATGCATATAAGCATGTACATAATATTATATCC  120
             ||||||||||||||||||||||||||||||||||||||||||||||||||||||||||||
Sbjct  80    TGTACGTCGTGCATTAATGGTTTGCCCCATGCATATAAGCATGTACATAATATTATATCC  139

Query  121   TTACATAGGACATATTAACTCAATCTCATAATTCACTGATCTATCAACAGTAATCGAATG  180
             ||||||||||||||||||||||||||||||||||||||||||||||||||||||||||||
Sbjct  140   TTACATAGGACATATTAACTCAATCTCATAATTCACTGATCTATCAACAGTAATCGAATG  199

Query  181   CATATCACTTAGTCCAATAAGGGCTTAATCACCATGCCTCGAGAAAC  227
             |||||||||||||||||||||||||||||||||||||||||||||||
Sbjct  200   CATATCACTTAGTCCAATAAGGGCTTAATCACCATGCCTCGAGAAAC  246


>gb|HQ997454.1| Canis lupus familiaris isolate 052 D-loop, partial sequence;
mitochondrial
 gb|HQ997468.1| Canis lupus familiaris isolate 087 D-loop, partial sequence;
mitochondrial
Length=672

 Score = 420 bits (227),  Expect = 6e-114
 Identities = 227/227 (100%), Gaps = 0/227 (0%)
 Strand=Plus/Plus

Query  1     TTGAATCACCCCTACTGTGCTATGTCAGTATCTCCAGGTAAACCCTTCTCCCCTCCCCTA  60
             ||||||||||||||||||||||||||||||||||||||||||||||||||||||||||||
Sbjct  20    TTGAATCACCCCTACTGTGCTATGTCAGTATCTCCAGGTAAACCCTTCTCCCCTCCCCTA  79

Query  61    TGTACGTCGTGCATTAATGGTTTGCCCCATGCATATAAGCATGTACATAATATTATATCC  120
             ||||||||||||||||||||||||||||||||||||||||||||||||||||||||||||
```

```
Sbjct  80   TGTACGTCGTGCATTAATGGTTTGCCCCATGCATATAAGCATGTACATAATATTATATCC  139

Query  121  TTACATAGGACATATTAACTCAATCTCATAATTCACTGATCTATCAACAGTAATCGAATG  180
            ||||||||||||||||||||||||||||||||||||||||||||||||||||||||||||
Sbjct  140  TTACATAGGACATATTAACTCAATCTCATAATTCACTGATCTATCAACAGTAATCGAATG  199

Query  181  CATATCACTTAGTCCAATAAGGGCTTAATCACCATGCCTCGAGAAAC  227
            |||||||||||||||||||||||||||||||||||||||||||||||
Sbjct  200  CATATCACTTAGTCCAATAAGGGCTTAATCACCATGCCTCGAGAAAC  246


>gb|HQ997424.1| Canis lupus familiaris isolate 004 D-loop, partial sequence;
mitochondrial
 gb|HQ997592.1| Canis lupus familiaris isolate 442 D-loop, partial sequence;
mitochondrial
Length=673

 Score = 420 bits (227),  Expect = 6e-114
 Identities = 227/227 (100%), Gaps = 0/227 (0%)
 Strand=Plus/Plus

Query  1    TTGAATCACCCCTACTGTGCTATGTCAGTATCTCCAGGTAAACCCTTCTCCCCTCCCCTA  60
            ||||||||||||||||||||||||||||||||||||||||||||||||||||||||||||
Sbjct  20   TTGAATCACCCCTACTGTGCTATGTCAGTATCTCCAGGTAAACCCTTCTCCCCTCCCCTA  79

Query  61   TGTACGTCGTGCATTAATGGTTTGCCCCATGCATATAAGCATGTACATAATATTATATCC  120
            ||||||||||||||||||||||||||||||||||||||||||||||||||||||||||||
Sbjct  80   TGTACGTCGTGCATTAATGGTTTGCCCCATGCATATAAGCATGTACATAATATTATATCC  139

Query  121  TTACATAGGACATATTAACTCAATCTCATAATTCACTGATCTATCAACAGTAATCGAATG  180
            ||||||||||||||||||||||||||||||||||||||||||||||||||||||||||||
Sbjct  140  TTACATAGGACATATTAACTCAATCTCATAATTCACTGATCTATCAACAGTAATCGAATG  199

Query  181  CATATCACTTAGTCCAATAAGGGCTTAATCACCATGCCTCGAGAAAC  227
            |||||||||||||||||||||||||||||||||||||||||||||||
Sbjct  200  CATATCACTTAGTCCAATAAGGGCTTAATCACCATGCCTCGAGAAAC  246


>gb|HM560932.1| Canis lupus familiaris haplotype Be61 D-loop, partial sequence;
mitochondrial
Length=1070

 Score = 420 bits (227),  Expect = 6e-114
 Identities = 227/227 (100%), Gaps = 0/227 (0%)
 Strand=Plus/Plus

Query  1    TTGAATCACCCCTACTGTGCTATGTCAGTATCTCCAGGTAAACCCTTCTCCCCTCCCCTA  60
            ||||||||||||||||||||||||||||||||||||||||||||||||||||||||||||
Sbjct  20   TTGAATCACCCCTACTGTGCTATGTCAGTATCTCCAGGTAAACCCTTCTCCCCTCCCCTA  79

Query  61   TGTACGTCGTGCATTAATGGTTTGCCCCATGCATATAAGCATGTACATAATATTATATCC  120
            ||||||||||||||||||||||||||||||||||||||||||||||||||||||||||||
Sbjct  80   TGTACGTCGTGCATTAATGGTTTGCCCCATGCATATAAGCATGTACATAATATTATATCC  139
```

```
Query  121  TTACATAGGACATATTAACTCAATCTCATAATTCACTGATCTATCAACAGTAATCGAATG  180
            ||||||||||||||||||||||||||||||||||||||||||||||||||||||||||||
Sbjct  140  TTACATAGGACATATTAACTCAATCTCATAATTCACTGATCTATCAACAGTAATCGAATG  199

Query  181  CATATCACTTAGTCCAATAAGGGCTTAATCACCATGCCTCGAGAAAC  227
            |||||||||||||||||||||||||||||||||||||||||||||||
Sbjct  200  CATATCACTTAGTCCAATAAGGGCTTAATCACCATGCCTCGAGAAAC  246


>gb|HQ845274.1| Canis lupus familiaris isolate K_38 breed Dwarf Schnauzer control
region, partial sequence; mitochondrial
Length=1272

 Score = 420 bits (227),  Expect = 6e-114
 Identities = 227/227 (100%), Gaps = 0/227 (0%)
 Strand=Plus/Plus

Query  1    TTGAATCACCCCTACTGTGCTATGTCAGTATCTCCAGGTAAACCCTTCTCCCCTCCCCTA  60
            ||||||||||||||||||||||||||||||||||||||||||||||||||||||||||||
Sbjct  20   TTGAATCACCCCTACTGTGCTATGTCAGTATCTCCAGGTAAACCCTTCTCCCCTCCCCTA  79

Query  61   TGTACGTCGTGCATTAATGGTTTGCCCCATGCATATAAGCATGTACATAATATTATATCC  120
            ||||||||||||||||||||||||||||||||||||||||||||||||||||||||||||
Sbjct  80   TGTACGTCGTGCATTAATGGTTTGCCCCATGCATATAAGCATGTACATAATATTATATCC  139

Query  121  TTACATAGGACATATTAACTCAATCTCATAATTCACTGATCTATCAACAGTAATCGAATG  180
            ||||||||||||||||||||||||||||||||||||||||||||||||||||||||||||
Sbjct  140  TTACATAGGACATATTAACTCAATCTCATAATTCACTGATCTATCAACAGTAATCGAATG  199

Query  181  CATATCACTTAGTCCAATAAGGGCTTAATCACCATGCCTCGAGAAAC  227
            |||||||||||||||||||||||||||||||||||||||||||||||
Sbjct  200  CATATCACTTAGTCCAATAAGGGCTTAATCACCATGCCTCGAGAAAC  246


>gb|HQ845266.1| Canis lupus familiaris isolate K_30 breed Chihuahua control region,
partial sequence; mitochondrial
Length=1248

 Score = 420 bits (227),  Expect = 6e-114
 Identities = 227/227 (100%), Gaps = 0/227 (0%)
 Strand=Plus/Plus

Query  1    TTGAATCACCCCTACTGTGCTATGTCAGTATCTCCAGGTAAACCCTTCTCCCCTCCCCTA  60
            ||||||||||||||||||||||||||||||||||||||||||||||||||||||||||||
Sbjct  20   TTGAATCACCCCTACTGTGCTATGTCAGTATCTCCAGGTAAACCCTTCTCCCCTCCCCTA  79

Query  61   TGTACGTCGTGCATTAATGGTTTGCCCCATGCATATAAGCATGTACATAATATTATATCC  120
            ||||||||||||||||||||||||||||||||||||||||||||||||||||||||||||
Sbjct  80   TGTACGTCGTGCATTAATGGTTTGCCCCATGCATATAAGCATGTACATAATATTATATCC  139

Query  121  TTACATAGGACATATTAACTCAATCTCATAATTCACTGATCTATCAACAGTAATCGAATG  180
            ||||||||||||||||||||||||||||||||||||||||||||||||||||||||||||
```

```
Sbjct  140  TTACATAGGACATATTAACTCAATCTCATAATTCACTGATCTATCAACAGTAATCGAATG  199

Query  181  CATATCACTTAGTCCAATAAGGGCTTAATCACCATGCCTCGAGAAAC  227
            |||||||||||||||||||||||||||||||||||||||||||||||
Sbjct  200  CATATCACTTAGTCCAATAAGGGCTTAATCACCATGCCTCGAGAAAC  246



>gb|HQ644192.1| Canis lupus familiaris isolate 87G ATP synthase F0 subunit 6
(ATP6), cytochrome c oxidase subunit III (COX3), and NADH dehydrogenase
subunit 4 (ND4) genes, partial cds; tRNA-His, tRNA-Ser,
and tRNA-Leu genes, complete sequence; NADH dehydrogenase
subunit 5 (ND5) gene, partial cds; and D-loop, partial
sequence; mitochondrial
Length=3228

 Score = 420 bits (227),  Expect = 6e-114
 Identities = 227/227 (100%), Gaps = 0/227 (0%)
 Strand=Plus/Plus

Query  1     TTGAATCACCCCTACTGTGCTATGTCAGTATCTCCAGGTAAACCCTTCTCCCCTCCCCTA  60
             ||||||||||||||||||||||||||||||||||||||||||||||||||||||||||||
Sbjct  2610  TTGAATCACCCCTACTGTGCTATGTCAGTATCTCCAGGTAAACCCTTCTCCCCTCCCCTA  2669

Query  61    TGTACGTCGTGCATTAATGGTTTGCCCCATGCATATAAGCATGTACATAATATTATATCC  120
             ||||||||||||||||||||||||||||||||||||||||||||||||||||||||||||
Sbjct  2670  TGTACGTCGTGCATTAATGGTTTGCCCCATGCATATAAGCATGTACATAATATTATATCC  2729

Query  121   TTACATAGGACATATTAACTCAATCTCATAATTCACTGATCTATCAACAGTAATCGAATG  180
             ||||||||||||||||||||||||||||||||||||||||||||||||||||||||||||
Sbjct  2730  TTACATAGGACATATTAACTCAATCTCATAATTCACTGATCTATCAACAGTAATCGAATG  2789

Query  181   CATATCACTTAGTCCAATAAGGGCTTAATCACCATGCCTCGAGAAAC  227
             |||||||||||||||||||||||||||||||||||||||||||||||
Sbjct  2790  CATATCACTTAGTCCAATAAGGGCTTAATCACCATGCCTCGAGAAAC  2836



>gb|HQ644148.1| Canis lupus familiaris isolate h21M ATP synthase F0 subunit 6
(ATP6), cytochrome c oxidase subunit III (COX3), and NADH dehydrogenase
subunit 4 (ND4) genes, partial cds; tRNA-His, tRNA-Ser,
and tRNA-Leu genes, complete sequence; NADH dehydrogenase
subunit 5 (ND5) gene, partial cds; and D-loop, partial
sequence; mitochondrial
Length=3228

 Score = 420 bits (227),  Expect = 6e-114
 Identities = 227/227 (100%), Gaps = 0/227 (0%)
 Strand=Plus/Plus

Query  1     TTGAATCACCCCTACTGTGCTATGTCAGTATCTCCAGGTAAACCCTTCTCCCCTCCCCTA  60
             ||||||||||||||||||||||||||||||||||||||||||||||||||||||||||||
Sbjct  2609  TTGAATCACCCCTACTGTGCTATGTCAGTATCTCCAGGTAAACCCTTCTCCCCTCCCCTA  2668

Query  61    TGTACGTCGTGCATTAATGGTTTGCCCCATGCATATAAGCATGTACATAATATTATATCC  120
```

```
              ||||||||||||||||||||||||||||||||||||||||||||||||||||||||||||
Sbjct  2669  TGTACGTCGTGCATTAATGGTTTGCCCCATGCATATAAGCATGTACATAATATTATATCC  2728

Query  121   TTACATAGGACATATTAACTCAATCTCATAATTCACTGATCTATCAACAGTAATCGAATG  180
              ||||||||||||||||||||||||||||||||||||||||||||||||||||||||||||
Sbjct  2729  TTACATAGGACATATTAACTCAATCTCATAATTCACTGATCTATCAACAGTAATCGAATG  2788

Query  181   CATATCACTTAGTCCAATAAGGGCTTAATCACCATGCCTCGAGAAAC  227
              |||||||||||||||||||||||||||||||||||||||||||||||
Sbjct  2789  CATATCACTTAGTCCAATAAGGGCTTAATCACCATGCCTCGAGAAAC  2835


>gb|HM561541.1| Canis lupus familiaris isolate K9_18 breed Fox Terrier control
region, partial sequence; mitochondrial
Length=1591

 Score = 420 bits (227),  Expect = 6e-114
 Identities = 227/227 (100%), Gaps = 0/227 (0%)
 Strand=Plus/Plus

Query  1    TTGAATCACCCCTACTGTGCTATGTCAGTATCTCCAGGTAAACCCTTCTCCCCTCCCCTA  60
            ||||||||||||||||||||||||||||||||||||||||||||||||||||||||||||
Sbjct  117  TTGAATCACCCCTACTGTGCTATGTCAGTATCTCCAGGTAAACCCTTCTCCCCTCCCCTA  176

Query  61   TGTACGTCGTGCATTAATGGTTTGCCCCATGCATATAAGCATGTACATAATATTATATCC  120
            ||||||||||||||||||||||||||||||||||||||||||||||||||||||||||||
Sbjct  177  TGTACGTCGTGCATTAATGGTTTGCCCCATGCATATAAGCATGTACATAATATTATATCC  236

Query  121  TTACATAGGACATATTAACTCAATCTCATAATTCACTGATCTATCAACAGTAATCGAATG  180
            ||||||||||||||||||||||||||||||||||||||||||||||||||||||||||||
Sbjct  237  TTACATAGGACATATTAACTCAATCTCATAATTCACTGATCTATCAACAGTAATCGAATG  296

Query  181  CATATCACTTAGTCCAATAAGGGCTTAATCACCATGCCTCGAGAAAC  227
            |||||||||||||||||||||||||||||||||||||||||||||||
Sbjct  297  CATATCACTTAGTCCAATAAGGGCTTAATCACCATGCCTCGAGAAAC  343


>gb|HM561539.1| Canis lupus familiaris isolate K9_16 breed South African Boerboele
control region, partial sequence; mitochondrial
Length=1591

 Score = 420 bits (227),  Expect = 6e-114
 Identities = 227/227 (100%), Gaps = 0/227 (0%)
 Strand=Plus/Plus

Query  1    TTGAATCACCCCTACTGTGCTATGTCAGTATCTCCAGGTAAACCCTTCTCCCCTCCCCTA  60
            ||||||||||||||||||||||||||||||||||||||||||||||||||||||||||||
Sbjct  117  TTGAATCACCCCTACTGTGCTATGTCAGTATCTCCAGGTAAACCCTTCTCCCCTCCCCTA  176

Query  61   TGTACGTCGTGCATTAATGGTTTGCCCCATGCATATAAGCATGTACATAATATTATATCC  120
            ||||||||||||||||||||||||||||||||||||||||||||||||||||||||||||
Sbjct  177  TGTACGTCGTGCATTAATGGTTTGCCCCATGCATATAAGCATGTACATAATATTATATCC  236
```

```
Query  121   TTACATAGGACATATTAACTCAATCTCATAATTCACTGATCTATCAACAGTAATCGAATG  180
             ||||||||||||||||||||||||||||||||||||||||||||||||||||||||||||
Sbjct  237   TTACATAGGACATATTAACTCAATCTCATAATTCACTGATCTATCAACAGTAATCGAATG  296

Query  181   CATATCACTTAGTCCAATAAGGGCTTAATCACCATGCCTCGAGAAAC  227
             |||||||||||||||||||||||||||||||||||||||||||||||
Sbjct  297   CATATCACTTAGTCCAATAAGGGCTTAATCACCATGCCTCGAGAAAC  343


>gb|AF531667.2| Canis familiaris isolate A14 control region, partial sequence
Length=609

 Score = 420 bits (227),  Expect = 6e-114
 Identities = 227/227 (100%), Gaps = 0/227 (0%)
 Strand=Plus/Plus

Query  1     TTGAATCACCCCTACTGTGCTATGTCAGTATCTCCAGGTAAACCCTTCTCCCCTCCCCTA  60
             ||||||||||||||||||||||||||||||||||||||||||||||||||||||||||||
Sbjct  47    TTGAATCACCCCTACTGTGCTATGTCAGTATCTCCAGGTAAACCCTTCTCCCCTCCCCTA  106

Query  61    TGTACGTCGTGCATTAATGGTTTGCCCCATGCATATAAGCATGTACATAATATTATATCC  120
             ||||||||||||||||||||||||||||||||||||||||||||||||||||||||||||
Sbjct  107   TGTACGTCGTGCATTAATGGTTTGCCCCATGCATATAAGCATGTACATAATATTATATCC  166

Query  121   TTACATAGGACATATTAACTCAATCTCATAATTCACTGATCTATCAACAGTAATCGAATG  180
             ||||||||||||||||||||||||||||||||||||||||||||||||||||||||||||
Sbjct  167   TTACATAGGACATATTAACTCAATCTCATAATTCACTGATCTATCAACAGTAATCGAATG  226

Query  181   CATATCACTTAGTCCAATAAGGGCTTAATCACCATGCCTCGAGAAAC  227
             |||||||||||||||||||||||||||||||||||||||||||||||
Sbjct  227   CATATCACTTAGTCCAATAAGGGCTTAATCACCATGCCTCGAGAAAC  273


>gb|HM560892.1| Canis lupus familiaris haplotype Be21 D-loop, partial sequence;
mitochondrial
Length=1070

 Score = 416 bits (225),  Expect = 7e-113
 Identities = 226/227 (99%), Gaps = 0/227 (0%)
 Strand=Plus/Plus

Query  1     TTGAATCACCCCTACTGTGCTATGTCAGTATCTCCAGGTAAACCCTTCTCCCCTCCCCTA  60
             ||||||||||||||||||||||||||||||||||||||||||||||||||||||||||||
Sbjct  20    TTGAATCACCCCTACTGTGCTATGTCAGTATCTCCAGGTAAACCCTTCTCCCCTCCCCTA  79

Query  61    TGTACGTCGTGCATTAATGGTTTGCCCCATGCATATAAGCATGTACATAATATTATATCC  120
             ||||||||||||||||||||||||||||||||||||||||||||||||||||||||||||
Sbjct  80    TGTACGTCGTGCATTAATGGTTTGCCCCATGCATATAAGCATGTACATAATATTATATCC  139

Query  121   TTACATAGGACATATTAACTCAATCTCATAATTCACTGATCTATCAACAGTAATCGAATG  180
             ||||||||||||||||||||||||||||||||||||| ||||||||||||||||||||||
Sbjct  140   TTACATAGGACATATTAACTCAATCTCATAATTCAYTGATCTATCAACAGTAATCGAATG  199
```

```
Query  181  CATATCACTTAGTCCAATAAGGGCTTAATCACCATGCCTCGAGAAAC  227
            |||||||||||||||||||||||||||||||||||||||||||||||
Sbjct  200  CATATCACTTAGTCCAATAAGGGCTTAATCACCATGCCTCGAGAAAC  246



  Database: Nucleotide collection (nt)
    Posted date:  Jul 5, 2015  11:00 AM
  Number of letters in database: 1,358,888,053
  Number of sequences in database:  31,070,346

Lambda      K        H
    1.33    0.621     1.12
Gapped
Lambda      K        H
    1.28    0.460    0.850
Matrix: blastn matrix:1 -2
Gap Penalties: Existence: 0, Extension: 0
Number of Sequences: 31070346
Number of Hits to DB: 1316254
Number of extensions: 589
Number of successful extensions: 589
Number of sequences better than 10: 494
Number of HSP's better than 10 without gapping: 0
Number of HSP's gapped: 494
Number of HSP's successfully gapped: 494
Length of query: 227
Length of database: 100143135861
Length adjustment: 32
Effective length of query: 195
Effective length of database: 99148884789
Effective search space: 19334032533855
Effective search space used: 19334032533855
A: 0
X1: 13 (25.0 bits)
X2: 32 (59.1 bits)
X3: 54 (99.7 bits)
S1: 13 (25.1 bits)
S2: 22 (41.7 bits)
```
